# Tropical fishes can benefit more from novel than familiar species interactions at their cold‐range edges

**DOI:** 10.1111/1365-2656.70100

**Published:** 2025-07-23

**Authors:** Angus Mitchell, Chloe Hayes, Ericka O. C. Coni, David J. Booth, Ivan Nagelkerken

**Affiliations:** ^1^ Southern Seas Ecology Laboratories, School of Biological Sciences The University of Adelaide Adelaide South Australia Australia; ^2^ School of the Life Sciences University of Technology Sydney Ultimo New South Wales Australia

**Keywords:** anti‐predator behaviour, competition, neophobia, ocean warming, phenotypic plasticity, range shifts, species interactions, tropical fish

## Abstract

1. Animals extending their biogeographic ranges poleward under global warming often interact with local species for limited resources such as food and shelter. Whether such novel species interactions facilitate or inhibit range extensions remains largely unknown.
2. We evaluated how range‐extending tropical and co‐shoaling temperate fishes modify their behaviours (aggression, foraging and anti‐predator) along a 2000‐km latitudinal gradient encapsulating tropical, subtropical and temperate reefs in a global ocean warming hotspot.
3. All five tropical fish species showed increased anti‐predator behaviours and decreased bite rate at their novel temperate range compared to their native tropical and subtropical ranges. However, when shoaling with temperate fishes, three of five tropical fish species had higher bite rates and all five tropical fish species spent less time sheltering compared to tropical‐only shoals, irrespective of biogeographic region.
4. In their subtropical ranges, tropical fish were more aggressive towards co‐shoaling temperate fish compared to their poleward novel cold ranges. This increased tropical fish aggression resulted in increased fleeing responses by the two temperate fishes at their subtropical warm trailing edges compared to their warm‐ and cold‐temperate core ranges.
5. Our findings suggest that tropical fish species trade‐off foraging efficiency for anti‐predator behaviour in their novel warm‐ and cold‐temperate ranges, independent of shoaling interactions.
6. However, shoaling with temperate species can increase the foraging efficiency of tropical fishes, which may be a mechanism (phenotypic plasticity) that enhances their performance at their leading temperate range edges.
7. Since novel species interactions enhanced the behavioural performance of some tropical fishes, we conclude that behavioural interactions between range‐extending and local species can facilitate successful range extensions of some species into novel environments.

## Graphical abstract

We reveal that shoaling with temperate fish enhances foraging efficiency in range‐extending tropical fish species, suggesting novel species interactions can aid poleward expansions in marine ecosystems. Our work highlights behavioural plasticity as a mechanism that can facilitate tropical fishes to adapt to novel temperate ecosystems under climate change.

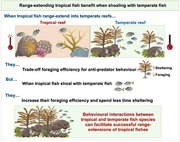

## 1 INTRODUCTION

Species redistributions are rapidly increasing under climate change (Burrows et al., 2011; Chen et al., 2011; Pecl et al., 2017). Global warming is driving species poleward to escape thermally unsuitable environments, allowing organisms to enter higher latitude ecosystems that have become thermally accessible due to anthropogenic warming (Pecl et al., 2017). Climate‐induced range shifts have facilitated novel interactions between local and range‐shifting species which can determine range shift and biodiversity outcomes in recipient ecosystems (Alexander et al., 2015; Twiname et al., 2022). Range extension success is often limited through environmental novelty posed by temperate ecosystems (e.g. lethal winter temperatures and novel predators and prey availability; Figueira et al., 2009; Booth et al., 2011) or by local species which can outcompete range extenders for resources (Coni et al., 2021). Hence, range‐extending species must overcome biotic resistance to establish at higher latitudes. Range‐shifting species may counter biotic resistance by modifying their behaviour to adapt to local conditions (Coni et al., 2022; Smith et al., 2018). Nevertheless, how range‐shifting species alter their behaviour to overcome biotic resistance remains largely untested, particularly for gregarious species which can show strong interactions with other individuals to determine group dynamics.

Range‐shifting species that show high phenotypic plasticity have a greater chance of establishing in new environments than species exhibiting limited plasticity (Donelson et al., 2019). Behavioural plasticity can allow range‐shifting species to modify their behaviour and increase persistence at their novel range edges (Coni et al., 2021; Smith et al., 2018). Species of recipient communities must also possess some degree of behavioural plasticity to compete against or limit the establishment of invading competitors (Coni et al., 2021). Behavioural plasticity can also allow resident species to adjust their behaviour at their trailing range edge, where the introduction of novel competitors is likely to occur (Coni et al., 2021). Hence, understanding whether behavioural shifts mediated by range shifts are beneficial to either resident or range‐shifting species is necessary to assess if species can adapt to novel ecological conditions under climate change.

Ocean warming and strengthening poleward boundary currents have facilitated the rapid poleward redistribution of tropical fishes into subtropical and temperate marine ecosystems globally (Booth et al., 2011; Vergés, Steinberg, et al., 2014). In eastern Australia, recipient temperate ecosystems experience above‐average warming rates (~3–4 times the global average; Lough, 2008), along with a strengthening of the East Australian Current (EAC; Suthers et al., 2011), which has facilitated the dispersal of >150 tropical fish species poleward into south‐eastern Australian marine ecosystems (Booth et al., 2011; Feary et al., 2014). These tropical species do not have established breeding populations at higher latitudes; instead, adults reside in warmer tropical reefs; and their pelagic larvae are transported southward by the EAC before settling and recruiting into temperate reef habitats. Tropical herbivores entering temperate marine ecosystems can alter ecosystem functioning by overgrazing kelp forests (Vergés, Tomas, et al., 2014), shifting ecosystems towards turf‐dominated habitats (Vergés, Steinberg, et al., 2014). These examples highlight how range‐extending tropical fishes could severely modify temperate ecosystems. Yet, whether and how biotic interactions among range‐extending and local fishes mediate range shift outcomes remains largely unknown.

Lethal winter temperatures in high latitude temperate regions can limit the range extension success of species entering from warmer lower latitudes (Figueira et al., 2009). Winter sea surface temperatures in temperate ecosystems can be detrimental to warm‐adapted tropical species through physiological (e.g. increased cellular stress and reduced growth; Mitchell et al., 2023a; Hayes et al., 2024) and behavioural impacts (e.g. decreased feeding, Mitchell et al., 2023b; slower predator escape responses, Djurichkovic et al., 2019; Figueira et al., 2019). Novel species interactions can enhance tropical fish fitness, alter physiology (Sasaki et al., 2024) and temper lethal winter temperature effects (Smith et al., 2018). However, when combined with the unknown risks posed by temperate ecosystems, sub‐optimal winter temperatures can degrade the fitness of range‐shifting species (Figueira et al., 2009), slowing down tropical fish range extensions at their cold range limits.

Predation risk and competition levels in novel environments are often unknown and unpredictable to range‐shifting species (Brown et al., 2022). Range‐extending species can escape historical predators and competitors under climate change but often gain new ones at their poleward range edges (Figueira et al., 2019). Thus, vulnerability to novel predators and competitors in new environments can increase due to a lack of eco‐evolutionary experiences shared between range‐extending and local species (Saul & Jeschke, 2015). Range‐extending individuals, which ignore or fail to identify risks, can experience increased predation (Figueira et al., 2019). Selection of differential behaviours may emerge through predation and competition pressures of certain behavioural responses (e.g. risk‐averse behaviours, Coni et al., 2022) and therefore constrain range extension success of some individuals but facilitate the success of others of the same species into novel environments (Canestrelli et al., 2016). Additionally, to reduce the risk of novel biotic factors (e.g. predators and competitors), tropical fishes can learn environmental and social cues at their new ranges through social interactions with local temperate species (Smith et al., 2018). Social learning can inform range‐shifting species about novel predators and enhance foraging opportunities (e.g. through olfactory and visual cues, Paijmans et al., 2020). Shoaling behaviour enhances anti‐predator performance in general, but also in novel environmental contexts (i.e. confusion and dilution effects, Paijmans et al., 2020). Shoaling species also have advantages over solitary species when entering novel environments, as shoaling behaviour can increase intra‐shoal information transfer, reduce individual predation risk and improve social learning (Smith et al., 2018). Shoaling tropical fishes are commonly observed at the forefront of range extensions in temperate south‐eastern Australia (Booth et al., 2007, 2011). Gregarious tropical damselfish species (e.g. the genus *Abudefduf*) are the most abundant tropical fishes observed range‐shifting into temperate ecosystems in Eastern Australia and often form shoals with morphologically similar local temperate species (Smith et al., 2018). Such evidence suggests that shoaling behaviour and novel interactions might benefit species range extensions. However, it remains largely unclear how co‐shoaling tropical and local temperate fishes alter their behaviour, and to what degree they benefit, or not, from shoaling interactions.

Shifts in foraging and anti‐predator behaviour (e.g. predator vigilance) are common in animals exposed to novel environmental contexts (Brown et al., 2022). Behavioural trade‐offs can arise from elevated competition and uncertain risk or foraging opportunities (Brown et al., 2022), all of which are present in temperate ecosystems for range‐extending tropical fishes (Coni et al., 2021). Risk‐averse behaviour, which range‐extending tropical fishes often show in temperate ecosystems, can either slow their establishment by reducing foraging opportunities (Coni et al., 2021) or provide fitness‐enhancing benefits by minimizing predation risk (Ferrari et al., 2015). Trade‐offs in behaviour can be expressed through modifications in shoaling (Bisazza & Dadda, 2005), anti‐predator (Coni et al., 2022) and foraging behaviour (Coni et al., 2021). Studies assessing behavioural responses of range‐shifting tropical fishes focus on individual‐level responses to single stressors (i.e. temperature, Djurichkovic et al., 2019). However, the true complexity and uncertainty of novel cold‐temperate range edges could facilitate differences in behavioural responses that shoal‐forming tropical fish experience under isolated climatic stressors. Therefore, shoal‐level responses of range‐shifting tropical fishes exposed to novel environments may reveal how behavioural modifications modulate species range shifts.

Here, we investigate how range‐extending tropical and co‐shoaling temperate fishes modify their foraging, sheltering behaviour and predator vigilance along a 2000‐km latitudinal gradient (latitude: 19.1°–36.9° S). While this gradient encapsulates the five focal tropical species' core ranges and novel leading edges, the two temperate species are absent from the tropical reefs and overlap across approximately 1000 km, from Tweed Heads (28.2° S) to Merimbula (36.9° S), where they co‐occur with tropical species in subtropical and temperate ecosystems. The cold‐range edge of tropical fishes in temperate ecosystems is a novel environment with new competitors, habitats, predators and lethal winter temperatures, potentially limiting the permanent establishment of tropical species (Coni et al., 2022). In response to these abiotic and biotic challenges, we hypothesise (1) that range‐extending tropical fishes will show increased anti‐predator behaviours (e.g. increased sheltering behaviour, flight initiation distances and lateralization) in their cold‐temperate ranges compared to their historical subtropical and tropical ranges, which may increase their ability to better persist in novel temperate ecosystems. Additionally, we hypothesise (2) that tropical fishes will modify their behaviours based on shoal composition, with mixed‐species shoaling (tropical‐temperate shoals) enhancing foraging efficiency (bite rate) through a combination of social learning and reduced predation risk at their cold‐range edge, compared to their historical home range. This study examines how these behavioural shifts interact with both geographic variation and shoal type to shape the ecological success of range‐extending fish at their leading cold‐range edges.

## 2 MATERIALS AND METHODS

### 2.1 Hypotheses and data collection

Hypothesis 1 Range‐extending tropical fishes show increased anti‐predator behaviours (increased sheltering, flight initiation distance and lateralization) in their novel warm‐ and cold‐temperate ranges compared to their historical subtropical and tropical ranges.

Hypothesis 2 Mixed‐species shoaling (tropical‐temperate shoals) can benefit tropical fishes by increasing foraging efficiency (bite rate) through social learning and may lower predation risk compared to tropical‐only shoals.

#### 2.1.1 Data collection for Hypothesis 1

To test hypothesis 1, we video recorded foraging, sheltering behaviour and flight initiation distance for five tropical and two temperate fish species and lateralization (relative and absolute lateralization) for one tropical and one temperate fish species across tropical, subtropical, warm‐temperate and cold‐temperate regions. Videos were used to capture foraging and anti‐predator behaviours, and a standard detour test was used to assess lateralization.

#### 2.1.2 Data collection for Hypothesis 2

To test hypothesis 2, we measured bite rates and aggressive interactions (chasing and fleeing) in mixed‐species, tropical‐only and temperate‐only shoals across sampling regions. We did so to assess whether shoal type (mixed, tropical or temperate shoals) modifies foraging efficiency, aggressive interactions and sheltering behaviours of co‐shoaling tropical and temperate fishes, and whether these behavioural modifications provide benefits (e.g. increased foraging efficiency).

### 2.2 Biogeographic regions and locations

**FIGURE 1:**
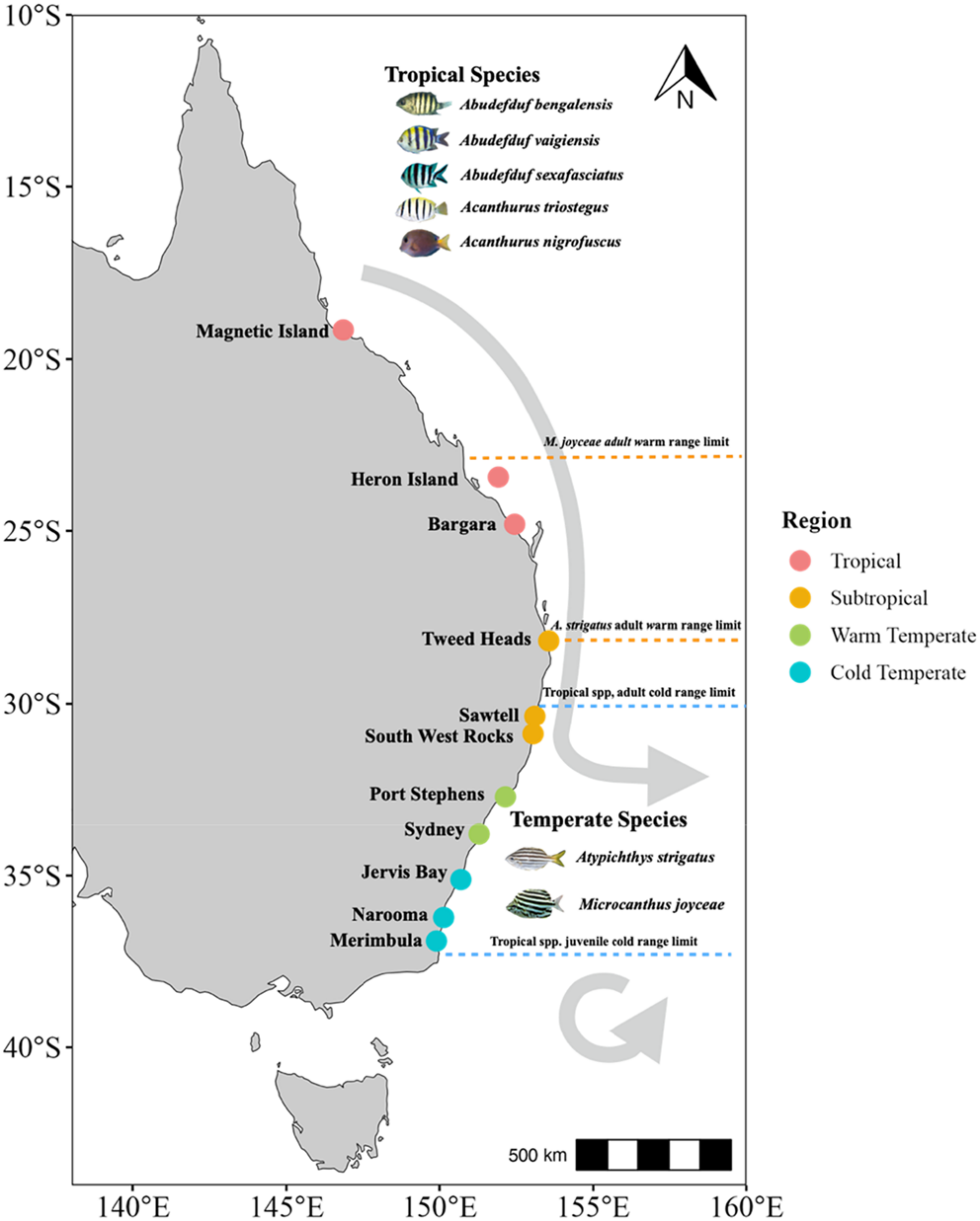
Map of the study regions and locations along the east Australian coastline. The East Australian Current disperses tropical fish larvae poleward from the tropical and subtropical regions into warm‐ and cold‐temperate reefs annually. Settling tropical juveniles often form shoals with juveniles of local temperate fishes at temperate reef locations. Coral coloured markers indicate locations in the tropical region: Magnetic Island (~19.1° S), Heron Island (~23.4° S) and Bargara (~24.8° S). Orange markers indicate locations in the subtropical region: Tweed Heads (~28.2° S), Sawtell (~30.4° S) and South West Rocks (30.9° S). Green markers indicate locations in the warm temperate region: Port Stephens (32.7° S) and four locations in northern Sydney (Little Manly, Freshwater Beach, Shelly Beach (~33.8° S) and Narrabeen lagoon (~33.7° S)). Blue markers show locations in the cold temperate region: Currawong Beach, Jervis Bay (~35° S), Narooma (~36.2° S) and Merimbula (~36.9° S). Dashed lines indicate the approximate warm range limit of the temperate species (*Microcanthus joyceae* and *Atypichthys strigatus*) and the cold range limit of adult and juvenile tropical species. The tropical species (*Abudefduf bengalensis*, *Abudefduf vaigiensis*, *Abudefduf sexfasciatus*, *Acanthurus triostegus* and *Acanthurus nigrofuscus*) occur primarily in the tropical and subtropical regions, but some juveniles settle further south in temperate reefs. The temperate species (*Atypichthys strigatus* and *Microcanthus joyceae*) are primarily found in temperate regions but have a northern range limit in the subtropical zone. All regions were sampled in 2017, 2018 and 2021, except the tropical region which was only sampled in 2021.

Biogeographic regions (Figure 1) were selected along a 2000‐km latitudinal gradient encapsulating the historical distributions of five range‐extending tropical fishes: (1) tropical region (latitude: 19.1–24.9° S), (2) subtropical region (latitude: 28.2–30.9° S) which includes the most southern breeding population of the tropical species (Booth et al., 2007), (3) warm‐temperate region (latitude: 33.8° S) where tropical fishes have been observed overwintering but not producing breeding populations (Booth et al., 2011) and (4) cold‐temperate region (latitude: 36.2–36.9° S), where juveniles of tropical fishes reside during summer months but fail to overwinter (Booth et al., 2011). The subtropical and temperate study sites were selected based on known locations of tropical fish recruitment at these locations in previous related field and survey studies (Booth et al., 2011; Coni et al., 2022). We collected behaviour and species interaction data at depths of 0.2–3 m during peak tropical recruitment in temperate ecosystems (Booth et al., 2007). Temperature data collected during video observation periods for locations are reported in Table S2 and in Hayes et al. (2025) and Coni et al. (2022). Data collection began in the cold‐temperate region and proceeded towards the tropical region each year, in 2017, 2018 and 2021.

### 2.3 Study species

We selected five commonly observed range‐extending fish species entering temperate marine ecosystems annually (Booth et al., 2011): three omnivorous fishes, the Indo‐Pacific sergeant (*Abudefduf vaigiensis*), the scissortail sergeant (*Abudefduf sexfasciatus*), the Bengal sergeant (*Abudefduf bengalensis*) and two herbivores, the dusky surgeonfish (*Acanthurus nigrofuscus*) and the convict surgeonfish (*Acanthurus triostegus*). At their cold‐range edge in temperate ecosystems, the tropical fish species often shoal with resident temperate fish species and form mixed‐species shoals (Smith et al., 2018). We chose the two most common co‐shoaling temperate species, the Australian mado (*Atypichthys strigatus*) and the East‐Australian stripey (*Microcanthus joyceae*), both of which are omnivorous. These seven focal species have coexisted seasonally during austral summer and autumn months in temperate ecosystems for at least the past 20 years but coexist in their subtropical ranges throughout the year (Coni et al., 2021). Because adults of the focal tropical species have not established breeding populations at the warm‐ and cold‐temperate regions (Booth et al., 2011), we focussed only on juveniles of the focal tropical and temperate fish species across the regions sampled in this study.

### 2.4 Sheltering, foraging behaviour and aggressive interactions in situ

To test hypotheses 1 and 2, we quantified sheltering, foraging behaviour and aggressive interactions between focal tropical and temperate fishes in situ. GoPro Hero 7 Silver cameras were placed in front of juvenile fish shoals (mean shoal size = 10.74 fish per shoal; shoal types: tropical only, mixed‐species (tropical and temperate) or temperate only; Figure S4) at a distance which encapsulated the entire shoal (~ 0.5–1.5 m) and recorded for 5–10 min (frame rate: 30 fps). Tropical shoals consisted of at least one of the following focal tropical species *Abudefduf vaigiensis*, *Abudefduf sexfasciatus*, *Abudefduf bengalensis*, *Acanthurus nigrofuscus*, *Acanthurus triostegus* and other tropical species (e.g. *Abudefduf whitleyi*). Temperate shoals consisted of at least one focal temperate species (*Atypichthys strigatus*, *Microcanthus joyceae*) and other shoaling temperate species (e.g. *Scorpis lineolata*), and mixed‐species shoals comprised of both focal tropical and temperate shoaling species. We recorded videos at depths of 0.2–3 m across the sampling regions (19.1° S–36.9° S; Figure 1). The first and last 1.5 min of video recordings were discarded during video shoal analysis to avoid recording unnatural behaviour caused by the presence of a snorkeller. Juveniles of the study species show high site attachment; thus, to minimise potential pseudoreplication, snorkellers spaced videos >15 m apart and recorded behavioural videos in reef locations only once before moving to other areas of sampling locations.

Using video recordings, we quantified foraging (bite rate) and aggressive interactions (chasing conspecifics, heterospecific tropical or temperate fishes, fleeing conspecifics, heterospecific tropical or temperate fishes; Table S1) for 20 s to 3‐min periods and converted each interaction and foraging behaviour into a rate (interactions.sec^−1^) for our two focal temperate fish species (*A. strigatus*, *M. joyceae*) and five tropical fish species (*A. vaigiensis*, *A. sexfasciatus*, *A. bengalensis*, *A. nigrofuscus*, *A. triostegus*) across tropical‐only, temperate‐only and mixed‐species shoals. We only analysed the behaviour of one fish per species per shoal to minimise potential pseudoreplication. Mean shoal size and mean proportion of conspecifics of the focal fish's species within the shoal (%) were quantified across 10 still frames over a 100‐s sequence.

### 2.5 Fish collection for lateralization tests

Lateralization, defined as the preference for certain behaviours to be performed by one side of the body (Rogers, 2021), is an important aspect of cognition observed across a wide range of animal species (Rogers, 2021). In shoaling fishes, lateralization at the individual and population level can influence anti‐predator behaviour, foraging efficiency and social interactions within shoals (Chivers et al., 2016). Lateralized individuals may perform better when escaping from predators compared to non‐lateralized individuals, but lateralization biases can reduce intra‐ and inter‐specific competitive abilities in shoaling fishes (Chivers et al., 2016), which may reduce the ability of fishes to persist in rapidly changing environments. Additionally, lateralized individuals often form more cohesive shoals (Bisazza & Dadda, 2005) which show superior escape performance than non‐cohesive shoals (Chivers et al., 2016), which could improve tropical fish survival in novel temperate ecosystems.

For tropical species (e.g. *Abudefduf vaigiensis*) that undergo range extensions and co‐shoaling temperate species (*Atypichthys strigatus*) studied here, lateralization may also play a key role in their ability to adapt to changing environments and interact with unfamiliar species. This is relevant in scenarios where these fishes encounter novel predators or competitors in temperate ecosystems under current ocean warming. Thus, we assessed lateralization for a tropical species (*Abudefduf vaigiensis*) and co‐shoaling temperate species (*Atypichthys strigatus*) across different biogeographic regions as a proxy for anti‐predator performance and to anticipate how shifts in their distributions could affect mixed‐species shoaling interactions in rapidly warming temperate ecosystems.

We selected one model species for each temperature affinity (tropical vs. temperate): (1) the most prevalent range‐extending tropical fish *Abudefduf vaigiensis* (Booth et al., 2011) and the morphologically similar and most common co‐shoaling temperate species *Atypichthys strigatus* (Smith et al., 2018). The distribution of the temperate species occurs from subtropical (southern Queensland) to temperate (southern New South Wales) Australian reefs. These two species have coexisted for longer periods at the subtropical region (during all seasons; Coni et al., 2021) compared to the warm and cold temperate regions where they only coexist during the summer months. The temperate species *Atypichthys strigatus* is absent from the tropical region.

*A. vaigiensis* (mean ± SE wet weight: 1.05 ± 0.14 g; length: 23.8 ± 0.8 mm) and *A. strigatus* (mean wet weight: 2.98 ± 0.24 g, length: 40.1 ± 1.5 mm) were collected using a 9:1 ethanol:clove oil spray and hand nets between 15 April and 12 July 2021. Fish were collected to infer how biogeographic region (tropical, subtropical, warm‐temperate or cold‐temperate) and shoal type (tropical‐only, mixed‐species or temperate‐only) affected their lateralization (a measure of cognition and anti‐predator behaviour; Bisazza & Dadda, 2005). Fish were collected haphazardly from tropical‐only, temperate‐only and mixed‐species shoals across all sampling regions. Collected fish were held in an aerated 50‐L plastic bucket filled with fresh seawater until lateralization testing was performed for each fish.

### 2.6 Motor lateralization test

We tested motor lateralization across sampling regions for 169 tropical (*A. vaigiensis*) and 150 temperate (*A. strigatus*) fishes from 12 to 4 pm between 15 April 2021 and 12 July 2021 (Table 1) using a standard detour test (*t*‐test; Bisazza et al., 1998). The arena used in this study consists of a two‐way T‐shape runway that allows scoring the direction of each individual's turn (e.g. left or right) over 12 consecutive trials (Figure S1). The arena consisted of a transparent plastic tank (IKEA product number: 198.856.46; 39 × 28 × 14 cm, length×width×height) with a runway in the middle (20 × 6 cm, length × width). At both ends of the runway, white acrylic barriers (12 × 12 × 1 cm) were positioned perpendicular to the runway. The runway was created by placing acrylic inserts onto each side of the arena to form the runway (20 × 6 × 15 cm). The water depth in the arena was 12 cm. The arena was placed on a shaded, level surface at the collection site.

**TABLE 1:** Replication for the anti‐predator and foraging behaviours, and antagonistic interactions assessed across regions for focal tropical and temperate fishes in tropical‐only, mixed‐species or temperate‐only shoals.

Proxy	Region			
	Tropical	Subtropical	Warm temperate	Cold temperate
Relative and absolute lateralization				
*Abudefduf vaigiensis*	Tropical‐only: 5 Mixed‐species: —	Tropical‐only: 23 Mixed‐species: 16	Tropical‐only: 40 Mixed‐species: 14	Tropical‐only: 32 Mixed‐species: 36
*Atypichthys strigatus*	—	Temperate‐only: — Mixed‐species: 6	Temperate‐only: 36 Mixed‐species: 28	Temperate‐only: 62 Mixed‐species: 18
Behaviour (foraging, sheltering and antagonistic interactions)				
*Abudefduf vaigiensis*	Tropical‐only: 27 Mixed‐species: —	Tropical‐only: 50 Mixed‐species: 11	Tropical‐only: 31 Mixed‐species: 40	Tropical‐only: 35 Mixed‐species: 41
*Abudefduf sexfasciatus*	Tropical‐only: 5 Mixed‐species: —	Tropical‐only: 11 Mixed‐species: 3	Tropical‐only: 5 Mixed‐species: 11	Tropical‐only: 4 Mixed‐species: 6
*Abudefduf bengalensis*	Tropical‐only: 26 Mixed‐species: —	Tropical‐only: 28 Mixed‐species: 4	Tropical‐only: 5 Mixed‐species: 9	Tropical‐only: 8 Mixed‐species: 10
*Acanthurus nigrofuscus*	Tropical‐only: 3 Mixed‐species: —	Tropical‐only: 20 Mixed‐species: 7	Tropical‐only: 19 Mixed‐species: 7	—
*Acanthurus triostegus*	—	Tropical‐only: 20 Mixed‐species: 4	Tropical‐only: 15 Mixed‐species: 6	—
*Atypichthys strigatus*	—	Temperate‐only: — Mixed‐species: 8	Temperate‐only: 17 Mixed‐species: 37	Temperate‐only: 34 Mixed‐species: 40
*Microcanthus joyceae*	—	Temperate‐only: 17 Mixed‐species: 6	Temperate‐only: 10 Mixed‐species: 28	Temperate‐only: 10 Mixed‐species: 17
Flight initiation distance				
*Abudefduf vaigiensis*	*N*: 10	*N*: 38	*N*: 35	*N*: 41
*Acanthurus nigrofuscus*	—	*N*: 9	*N*: 12	—
*Acanthurus triostegus*	—	*N*: 14	*N*: 11	—
*Atypichthys strigatus*	—	—	*N*: 37	*N*: 48
*Microcanthus joyceae*	—	*N*: 18	*N*: 21	*N*: 19

*Note*: ‘—’ = not assessed for the focal species in the specified region, as the species was either not present or found in low numbers (*n* < 3) and excluded from our analysis. We did not consider shoal type for flight initiation distance sampling due to low shoal type replication of either level (*n* < 3) for all species across at least one region sampled.

At the start of each trial, a single fish was introduced from the 50‐L bucket into the arena and allowed to acclimate to the arena environment for 2 min. We conducted 12 consecutive turning trials for each individual. During each trial, we chased the focal fish with a small handheld net along the runway to force a left or right turning choice at the end of the runway (see Mitchell et al., 2022). The primary observer was always positioned at the same end of the arena to minimize side bias. A secondary observer stood behind the primary observer to validate each fish turning decision. After each trial, the experimental arena was emptied and refilled with fresh seawater until all collected fish were scored.

We calculated mean relative lateralization index (L_R_) and absolute lateralization (L_A_) from the 12 turning decisions (per fish) and used to identify the strength of population‐level laterality and turn preference (e.g. bias in left or right turns). For the L_R_ index, individuals were classified between the extreme values of ‘+100’ (fish that turned right on all 12 turning decisions) and ‘−100’ (fish that turned left on all 12 turning decisions). An individual L_R_ close to zero indicates that a fish is neither left‐ nor right‐biased in its turning tendency (Bisazza et al., 1998). The L_A_ index corresponds to the absolute value of L_R_, thus ranging from 0 (an individual that turned in equal proportion to the right and the left) to 100 (an individual that turned right or left on all 12 trials).

### 2.7 Flight initiation distance in situ

We defined the flight initiation distance ‘as the distance at which a fish initiates an escape response towards an artificial threat’. We tested the flight initiation distance of tropical fish species (species: *A. vaigiensis*, *A. nigrofuscus*, *A. triostegus*) and temperate fish species (species: *A. strigatus*; *M. joyceae*). *A. bengalensis* and *A. sexfasciatus* were excluded from flight initiation distance trials due to low sample size (*n* < 3) for each species across regions.

An artificial threat‐eliciting stimulus was used to mimic a predator attack. The predator threat was created using a cubical PVC frame connected to a 60‐cm iron rod, which supported a 30‐cm metal ruler at its most distal end (following methods in Nagelkerken et al., 2017). A GoPro Hero Silver 7 camera was fixed to the cubical frame and positioned towards the ruler. Once a focal juvenile fish was found, the snorkeler slowly approached the fish and moved the end of the ruler from above the fish towards its head at a constant speed while the camera was recording its escape behaviour (frame rate: 30fps; Figure S2). To avoid pseudoreplication, we sampled flight initiation distance of one individual fish per shoal and sampled each location over a 1‐day period. Flight initiation distances were calibrated according to the size of the attached metal ruler and quantified on the software VLC media player. Flight initiation distance data presented in this study combine new data collected in 2021 and published data (Coni et al., 2022).

### 2.8 Statistical analysis

We used generalized linear mixed models (GLMMs) to quantify the relationships between bite rate, sheltering behaviour, lateralization, flight initiation distance and aggressive interactions of co‐shoaling tropical and temperate fishes across different shoal types and biogeographic regions. Tropical and temperate species were analysed separately due to ecological and behavioural differences. Tropical and temperate species were sampled in different regions, introducing distinct environmental and ecological pressures that could influence their behaviours and interactions. Additionally, we expected behavioural differences between warm‐trailing temperate species and cold‐leading tropical species, which could drive variation in lateralization, foraging efficiency and predator avoidance strategies. Analysing tropical and temperate species separately allowed us to explore these differences without assuming uniform behavioural responses across species.

For behaviour and interaction models, we first considered models that included the fixed factors shoal type, region and species. Shoal type was classified as mixed‐species and tropical‐only shoals for tropical species and mixed‐species and temperate‐only shoals for temperate species. Region included four levels for tropical species (tropical, subtropical, warm‐temperate and cold‐temperate) and three levels for temperate species (subtropical, warm‐temperate and cold‐temperate). Species was modelled as a fixed factor, including five tropical species (*Abudefduf vaigiensis*, *Abudefduf sexfasciatus*, *Abudefduf bengalensis*, *Acanthurus nigrofuscus* and *Acanthurus triostegus*) and two temperate species (*Atypichthys strigatus* and *Microcanthus joyceae*). To account for variation in shoal structure across regions, we included shoal size and proportion of conspecifics in the shoal as continuous predictor variables. Location was modelled as a random intercept nested within region to account for site‐specific effects on fish behaviour.

GLMMs were fitted using the *glmmTMB* package (Brooks et al., 2017), and model selection was based on the Akaike information criterion (AIC), with final models retained based on AIC rankings, model assumptions and fit diagnostics. If the best‐ranked model violated GLMM assumptions, the next‐best model was selected for analysis. Given the zero‐inflated nature of bite rate, sheltering behaviour and interaction metrics, models were fitted using Tweedie, Gamma or Gaussian distributions, selected based on data structure, overdispersion and model diagnostics. Log transformations (log(*X* + 1)) or Box–Cox transformations were applied to improve residual normality, except for temperate species' relative lateralization, which met model assumptions in its raw form. A constant of +100 was added before log‐transforming the tropical species' relative lateralization values to accommodate negative values. Pseudo‐marginal and conditional *R* ^2^ values were calculated for all models.

GLMM assumptions were assessed using *DHARMa* residual diagnostics (Hartig, 2024), which included Q‐Q plots to assess normality, residual versus fitted plots to check for homoscedasticity, Kolmogorov–Smirnov (KS) tests to evaluate deviations from expected residual distributions and dispersion and outlier tests to detect potential model misspecifications. All 22 models met GLMM assumptions except for the tropical fish flight initiation distance model, which had significant heterogeneity of variance (*p* < 0.05; Figure S3i). However, for the latter model, no extreme departures from model assumptions were observed (Figures S3 and S4), and GLMMs are well suited for ecological data where minor deviations from assumptions are expected (Bolker et al., 2009). Hierarchical ecological datasets often contain structured variance due to nested dependencies, and GLMMs help account for this variance through random effects, making them robust to assumption violations (Bolker et al., 2009; Harrison et al., 2018). Previous studies have shown that minor violations of homoscedasticity in GLMMs do not necessarily bias estimates, particularly when model selection is guided by AIC (Schielzeth et al., 2020). Given the robustness of GLMMs, we retained the best‐fitting tropical fish flight initiation distance model to ensure that residual distributions remained appropriate for valid inference while preserving ecologically meaningful patterns in risk perception. Bite rate models for tropical fish were zero‐inflated Tweedie models due to frequent zero‐valued observations. Sheltering behaviour, flight initiation distance and aggressive interactions (chasing and fleeing) were fitted using zero‐inflated Tweedie, Gamma or Gaussian models to account for zero inflation and overdispersion. The interaction models for ‘tropical fish fleeing heterospecific tropical fish’ and ‘temperate fish fleeing heterospecific temperate fish’ retained the null model based on AIC rankings and are reported in the Supporting Information (Tables S11 and S23). Flight initiation distance models did not include shoal type as a factor due to low replication (*n* < 3 per shoal type level) across species at multiple regions.

To determine significant predictors in GLMMs, Type III Wald chi‐squared tests were conducted using the *car* package (Fox & Weisberg, 2019). Where main effects or interactions were significant (*p* < 0.05), Tukey post hoc tests were performed using the *emmeans* package (Lenth, 2024). All statistical analyses were conducted in R version 4.4.0 (R Core Team, 2024).

## 3 RESULTS

### 3.1 Tropical fish behaviour across regions and shoal types

**FIGURE 2:**
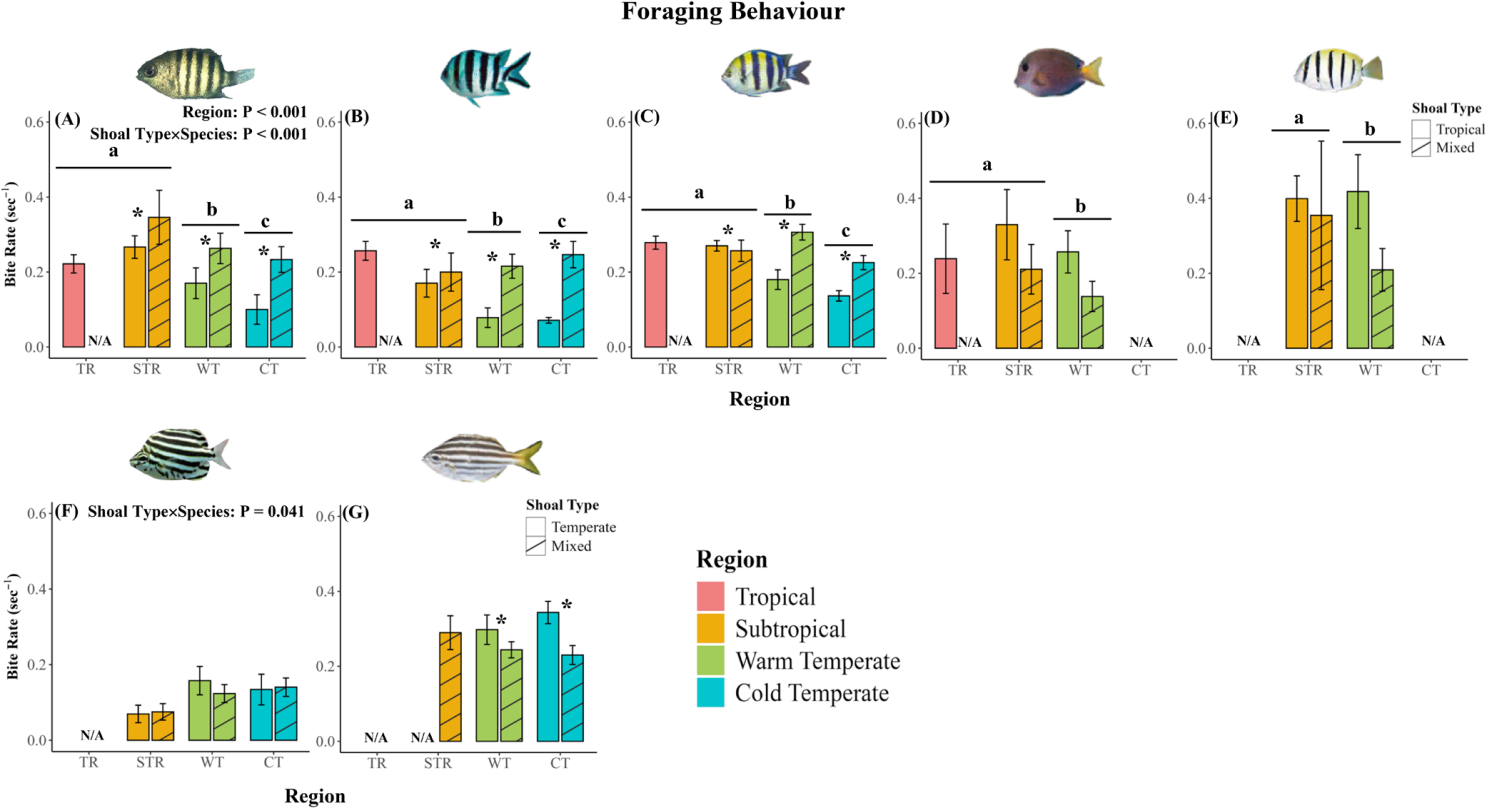
In situ foraging behaviour (bites.sec^−1^) of the tropical fish species (A) *Abudefduf bengalensis*, (B) *Abudefduf sexfasciatus*, (C) *Abudefduf vaigiensis*, (D) *Acanthurus nigrofuscus* and (E) *Acanthurus triostegus* in tropical‐only shoals (solid bars) and in mixed‐species shoals (hatched bars), and bite rates (bites.sec^−1^) of the focal temperate fish species (F) *Microcanthus joyceae* and (G) *Atypichthys strigatus* in temperate‐only shoals (solid bars) and in mixed‐species shoals (hatched) across regions (TR = Tropical; STR = Subtropical; WT = Warm temperate, CT = Cold temperate). Different letters above bars indicate significant differences among regions (*p* < 0.05; Tables S3 and S15). Error bars represent standard errors. N/A denotes that fish species was not sampled in the specified shoal type within a region or that focal species was not sampled within specific region. * denotes significant difference (*p* < 0.05) between shoal types.

**FIGURE 3:**
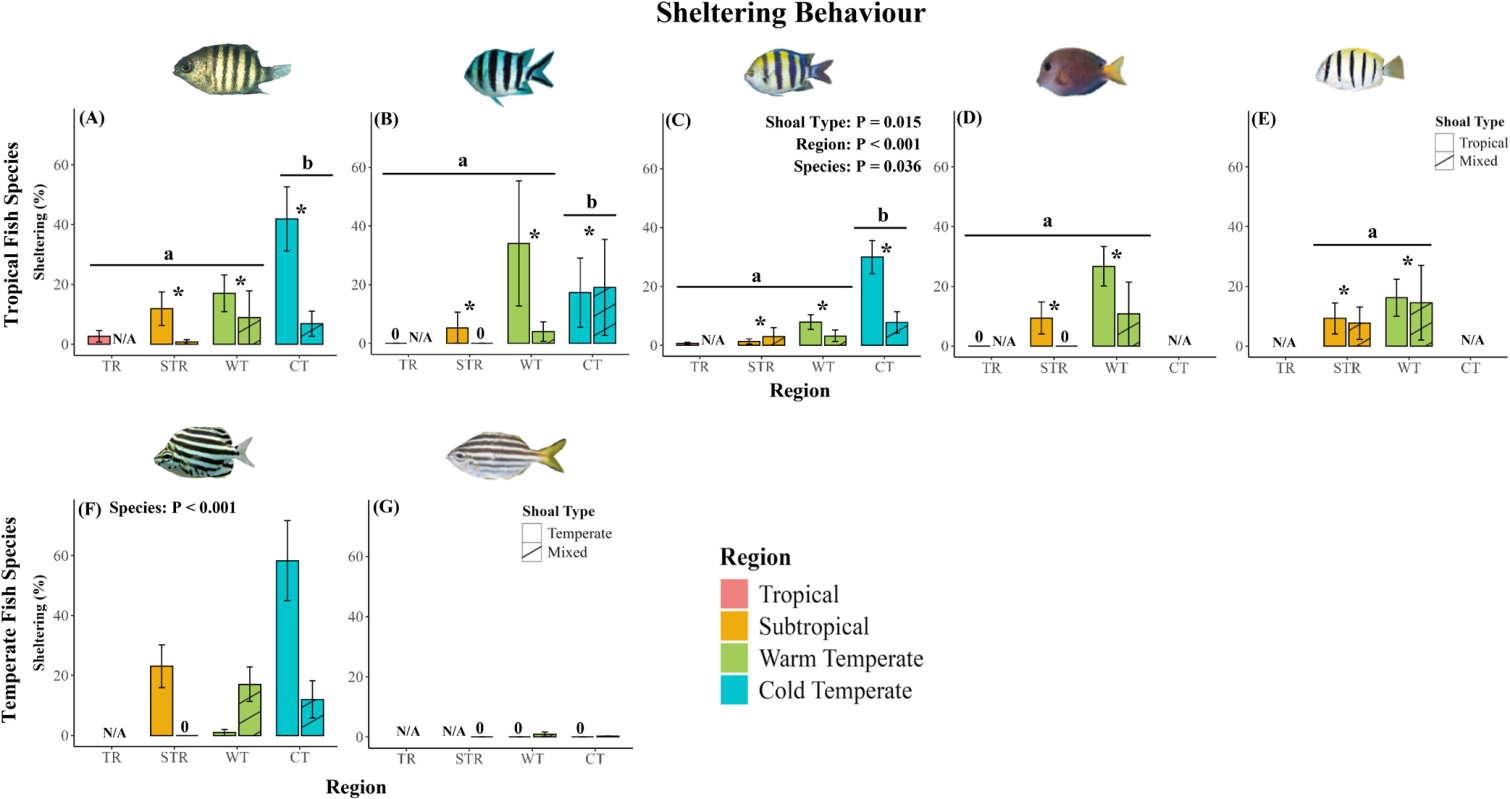
In situ sheltering behaviour (% time spent sheltering) of the tropical fish species (A) *Abudefduf bengalensis*, (B) *Abudefduf sexfasciatus*, (C) *Abudefduf vaigiensis*, (D) *Acanthurus nigrofuscus* and (E) *Acanthurus triostegus* in tropical‐only shoals (solid bars) and in mixed‐species shoals (hatched bars), and sheltering behaviour (% time spent sheltering) of the temperate fish species (F) *Microcanthus joyceae* and (G) *Atypichthys strigatus* in temperate‐only shoals (solid bars) and in mixed‐species shoals (hatched) across regions (TR = Tropical; STR = Subtropical; WT = Warm temperate, CT = Cold temperate). Different letters above bars indicate significant differences among regions (*p* < 0.05; Tables S4 and S17). Error bars represent standard errors. N/A denotes that fish species was not sampled in the specified shoal type within a region or that focal species was not sampled within specific region. * denotes significant difference (*p* < 0.05) between shoal types.

**FIGURE 4:**
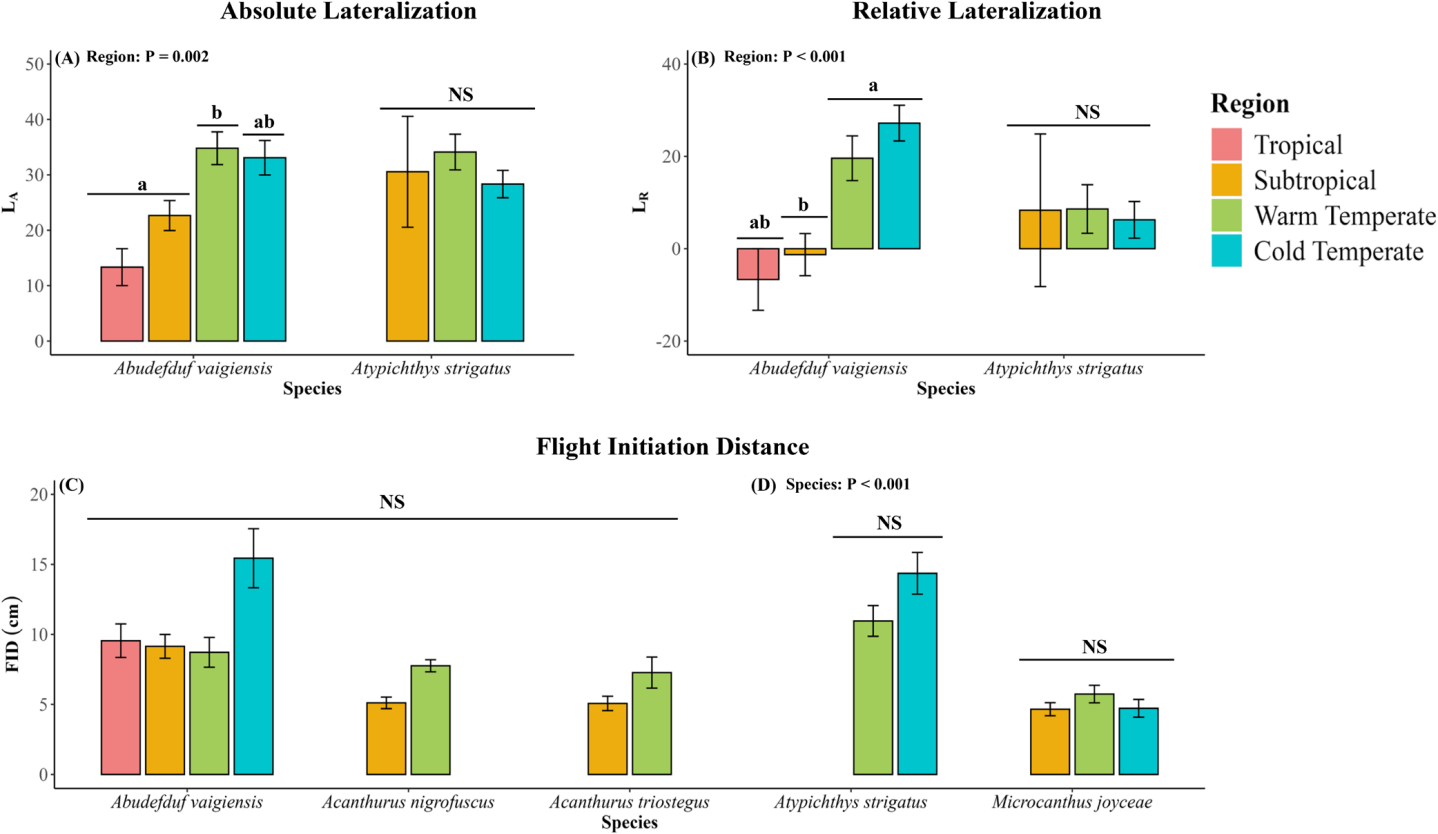
Absolute lateralization (A; L_A_; a measure of individual lateralization) and relative lateralization (B; L_R_; a measure of population‐level lateralization) of the tropical fish species *Abudefduf vaigiensis* and of the temperate fish *Atypichthys strigatus* across sampled regions (TR = Tropical; STR = Subtropical; WT = Warm temperate, CT = Cold temperate). (C, D) The flight initiation distance of three focal tropical fishes (C): *Abudefduf vaigiensis*, *Acanthurus nigrofuscus* and *Acanthurus triostegus* and two focal temperate fishes (D): *Atypichthys strigatus* and *Microcanthus joyceae* sampled across regions where focal fish species were present. Different letters above bars indicate significant differences among regions (*p* < 0.05; Tables S6–S8 and S18–S20). Error bars represent standard errors. NS = not significant (*p* > 0.05). Factor ‘shoal type’ was pooled for visualization.

In their novel warm‐ and cold‐temperate ranges, all tropical species showed lower foraging rates (Figure 2A−E; *p* ≤ 0.024; Table S3), spent more time sheltering (Figure 3A−E; *p* < 0.001; Table S4) and chased heterospecific temperate fishes less often (Figure S6A–E; *p* = 0.015; Table S5) compared to their core tropical and subtropical ranges. Additionally, the tropical fish species (*A. vaigiensis*) tested for lateralization had higher relative lateralization (L_R_) and absolute lateralization (L_A_) than in the subtropical region (Figure 4A,B; *p* = 0.023; Tables S6 and S7). Irrespective of region, all five tropical fishes chased and fled from heterospecific temperate fish more often in mixed‐species shoals than tropical‐only shoals (Figures S6A–E and S8A–E; *p* ≤ 0.017; Tables S5 and S12), while three of the five tropical fishes (*A. bengalensis*, *A. sexfasciatus* and *A. vaigiensis*) showed higher foraging in mixed‐species shoals than in tropical‐only shoals (Figure 2A−C; *p* ≤ 0.013; Table S3). The two herbivorous tropical fish species (*A. nigrofuscus* and *A. triostegus*) showed no differences in foraging behaviour between tropical‐only and mixed‐species shoals (Figure 2D,E; *p* ≥ 0.102; Table S3). All tropical fish species showed lower sheltering (Figure 3A–E; *p* = 0.015; Table S4) in mixed‐species shoals compared to tropical‐only shoals. Flight initiation distance and the rates of chasing and fleeing by focal tropical fish species in response to heterospecific tropical fishes were unaffected by region or shoal type. Similarly, the rates of conspecific antagonistic interactions (chasing and fleeing) among focal tropical species were also unaffected across regions and shoal types studied (Figure 4A–E; Figures S5A–E, S9A–E and S10A–E; *p* ≥ 0.076; Tables S8–S11 and S13).

### 3.2 Temperate fish behaviour across regions and shoal types

In their warm‐trailing range at the subtropical region, both temperate fish species fled from nearby tropical fishes more often compared to their warm‐ and cold‐temperate ranges (Figure S7; *p* < 0.001; Table S14). When co‐shoaling with tropical fishes, one temperate fish species (*A. strigatus*) had lower foraging behaviour (Figure 2F; *p* = 0.002; Table S15), while both temperate fish species chased conspecifics more often in mixed‐species shoals compared to temperate‐only shoals (Figure S9; *p* = 0.023; Table S16), irrespective of region. Sheltering behaviour, flight initiation distance, lateralization proxies (L_R_ and L_A_), chasing of heterospecific tropical and temperate fishes by focal temperate fishes and fleeing from heterospecific and conspecific temperate fishes by focal temperate fishes remained unaffected across regions and shoal types for both temperate species studied (Figure 3F–G; Figures S6F–G, S8F–G and S10F–G; *p* ≥ 0.196; Tables S17–S24).

## 4 DISCUSSION

Here, we reveal that novel species interactions (tropical‐temperate species co‐shoaling) resulting from climate‐driven range extensions by tropical species can affect a wide suite of behaviours that could benefit some tropical fishes in their novel ranges. First, while the bite rate of all five tropical fishes tested was lower in their novel cold‐ and warm‐temperate ranges compared to their native subtropical and tropical ranges, three tropical species showed increased bite rates in mixed‐species shoals compared to tropical‐only shoals irrespective of biogeographic region. This suggests that mixed‐species shoaling can enhance foraging efficiency in both their native subtropical and novel temperate ranges and allows tropical fishes to obtain larger body sizes compared to tropical‐only shoals (Smith et al., 2018). Although mixed‐species shoaling enhances foraging efficiency in range‐extending tropical fishes, temperature‐driven physiological constraints may still limit bite rates in colder temperate regions compared to the (sub)tropical regions (Table S2). Suboptimal temperatures can suppress metabolic activity and foraging rates of range‐extending tropical fish (Figueira et al., 2009), contributing to observed reductions in bite rate, independent of shoal composition. However, under future ocean warming, cold‐temperature constraints may lessen, altering interactions between temperature‐driven physiological limitations and shoaling‐driven foraging benefits in their novel temperate ranges. As metabolic rates increase under future ocean warming, range‐extending species may show higher foraging efficiencies in temperate ecosystems, further facilitating their persistence in temperate regions (Mitchell et al., 2023a). Tropical fishes can also be outcompeted for food items by local competitors in temperate ecosystems (Coni et al., 2021). Yet, the formation of same‐species subgroups within mixed‐species shoals and trophic niche segregation (Kingsbury et al., 2020) may allow tropical fishes to reduce aggressive interactions and inter‐species competition and minimise predation‐mortality risk (e.g. novel predator detection; Paijmans et al., 2020, 2021; dilution and confusion effects; Paijmans et al., 2020), while enhancing social learning from temperate shoal mates (Smith et al., 2018). These benefits suggest that tropical fishes can possess some degree of behavioural plasticity and the ability to learn directly from local temperate species, which allows for performance‐enhancing behavioural modifications, which in turn, may facilitate increased foraging (this study) and extended persistence at their cold temperate range limits (Smith et al., 2018). Thus, mixed‐species shoaling may enhance tropical fish performance in novel temperate environments and facilitate their range extensions. Our findings reveal the potential underlying mechanism of how mixed‐species shoaling can boost the performance of range‐extending tropical fish compared to tropical‐only shoals in novel temperate ecosystems. We conclude that novel species interactions may strengthen range extension success by increasing the foraging efficiency of range‐extending tropical fishes in novel temperate environments.

While novel mixed‐species shoaling can provide foraging benefits to tropical fishes, in tropical‐only shoals, tropical fishes trade off foraging efficiency for collective predator vigilance at their warm‐ and cold‐temperate range limit compared to their native subtropical and tropical ranges. Increased sheltering behaviour and increased population‐level lateralization and reductions in bite rate at all studied tropical species' respective cold‐range edges in tropical‐only shoals suggest that tropical fishes increase anti‐predator behaviour and reduce foraging compared to their subtropical and tropical native ranges. Shifts in behavioural trade‐offs can prove costly (e.g. reduced foraging efficiency (this study)), but this might be outweighed by enhanced survival for inexperienced juvenile shoaling tropical fish in novel contexts (e.g. minimize novel predation risk; Coni et al., 2022). Hence, shifts in trade‐offs between foraging and sheltering behaviour may be a plastic behavioural response that tropical fishes express towards environmental uncertainty and suboptimal temperatures experienced at their novel poleward range limits. In addition to trade‐offs, increased population‐level lateralization and sheltering by tropical fishes may act as a compensatory mechanism, whereby tropical fishes experiencing suboptimal physiological functioning (Hayes et al., 2024) increase anti‐predator behaviours to compensate for reductions in burst swim speed (Djurichkovic et al., 2019), physiological condition (Hayes et al., 2024, 2025), growth (Figueira et al., 2009) and foraging behaviour experienced under suboptimal cold temperatures in temperate ecosystems (Coni et al., 2021). Alternatively, selective pressure posed by novel temperate environments may facilitate the emergence of lateralized and risk‐averse (Coni et al., 2022) tropical fish populations in their cold‐range edges, resulting in behavioural polymorphisms between cold‐range and core (sub)tropical range populations (Canestrelli et al., 2016). Nevertheless, our findings suggest that elevated population‐level and risk‐averse sheltering behaviours expressed by tropical fishes may boost anti‐predator performance (e.g. increased predator avoidance) but at the cost of foraging efficiency at their novel warm‐ and cold‐temperate range limits. We conclude that range‐extending tropical fish trade‐off foraging efficiency for predator vigilance in their warm‐ and cold‐temperate regions in response to the uncertain risk and suboptimal temperatures posed by these novel temperate ecosystems, although this appears to be partly alleviated by shoaling with temperate species.

Two of the five tropical fish species studied here, *A. nigrofuscus* and *A. triostegus*, did not benefit from shoaling interactions with temperate species across their ranges tested. Both *A. nigrofuscus* and *A. triostegus* maintained foraging behaviour across shoal types, but had lower sheltering in mixed‐species shoals, which could increase predation risk in novel temperate ecosystems (Coni et al., 2022). We suggest that the specialised herbivorous feeding strategies of *A. nigrofuscus* and *A. triostegus* may limit their ability to learn from or adopt the behaviours of co‐shoaling omnivorous temperate fishes (Kingsbury et al., 2020). Thus, we conclude that a mismatch in feeding modes between the two tropical herbivores and their omnivorous temperate counterparts may constrain range‐extending herbivorous fishes to benefit from tropical‐temperate shoaling interactions under current ocean warming.

The two temperate fish species studied were negatively affected by mixed‐species shoaling interactions across subtropical and temperate ranges. Both species showed increased fleeing responses towards co‐shoaling tropical fishes in their trailing subtropical ranges, while one species (*A. strigatus*) had lower foraging behaviour when co‐shoaling with tropical fishes. Indeed, the formation of temperate intra‐species groups within mixed‐species shoals may have minimised some negative species interactions between tropical and temperate fishes (Paijmans et al., 2021), which could influence temperate fish behaviour. However, increased subordinate behaviour (fleeing responses) can limit both temperate species resource acquisition and their ability to meet elevated metabolic demands at their warm range edges. Under ocean warming, increasing tropical fish aggression (Mitchell et al., 2023a), degraded temperate shoaling abilities (Mitchell et al., 2022) and low plasticity in anti‐predator and foraging behaviours of the temperate fishes studied (Coni et al., 2022) may further limit their ability to respond to novel tropical predators and aggressive tropical competitors, hindering their persistence at their warm trailing range edges in a future ocean.

## 5 CONCLUSIONS

We reveal that prevalent range‐extending tropical fishes trade‐off foraging efficiency for predator vigilance (i.e. higher population‐level lateralization and sheltering behaviour) at their novel warm‐ and cold‐temperate range region compared to their native (sub)tropical ranges, independent of tropical‐temperate shoaling interactions. However, tropical‐temperate shoaling can increase the foraging efficiency of tropical fishes, which may allow them to persist longer or perform better at their leading cold‐water range edges. Both temperate fish species showed negative behavioural adjustments (lower foraging and higher fleeing responses) in response to tropical fishes across their temperate and subtropical ranges, which may limit their future persistence at their warm trailing ranges under ocean warming. We find that novel species interactions (mixed‐species shoaling) can provide greater benefit than familiar interactions and behavioural trade‐offs expressed by tropical fishes at their cold‐range edges and, therefore, may increase tropical fish range extension success by enhancing foraging behaviour in novel temperate environments.

## AUTHOR CONTRIBUTIONS

Ivan Nagelkerken, David J. Booth and Angus Mitchell conceptualized the project. Angus Mitchell, Ericka O. C. Coni and Chloe Hayes conducted fieldwork. Angus Mitchell, Chloe Hayes and Ericka O. C. Coni collected and analysed the data. Angus Mitchell wrote the manuscript. All authors reviewed and provided feedback on the manuscript.

## CONFLICT OF INTEREST STATEMENT

The authors have no conflicts of interest to declare.

## ETHICS STATEMENT

This research was conducted according to The University of Adelaide Animal Ethics and University of Technology Sydney permits: S‐2020‐13, S‐2015‐222A, S‐2017‐002 2017–1117, ETH17‐1117 and under NSW DPI Scientific Collection Permit: F94/696(A)‐9.0, Queensland General Fisheries Permit: 212884 and Great Barrier Reef Marine Park Permits: G20/43958.1 and G12/45557.1.

## Supporting information


**Table S1:** Ethogram of behaviours and aggressive interactions measured from video recordings record across regions and shoal types sampled.
**Table S2:** Mean summer seawater temperatures (°C) collected using HOBO temperature recorders at the same time as the recording of fish behaviours during 2017 and 2018.
**Table S3:** GLMM, Type III Wald Chi‐Square tests and resulting Tukey post hoc tests of all tropical fish species bite rate.
**Table S4:** GLMM, Type III Wald Chi‐Square tests and resulting Tukey post hocs of all tropical fish species' sheltering behaviour.
**Table S5:** GLMM, Type III Wald Chi‐Square tests and resulting Tukey post hoc tests of all tropical fish species' chasing behaviour towards heterospecific temperate fish responses.
**Table S6:** GLMM, Type III Wald Chi‐Square tests and resulting Tukey post hoc tests of the focal tropical fish's (*Abudefduf vaigiensis*) relative lateralization (L_R_) responses.
**Table S7:** GLMM, Type III Wald Chi‐Square tests and resulting Tukey post hoc tests of the focal tropical fish's (*Abudefduf vaigiensis*) absolute lateralization (L_A_) responses.
**Table S8:** GLMM, Type III Wald Chi‐Square tests and resulting Tukey post hocs of all tropical fish species' flight initiation distance responses.
**Table S9:** GLMM, Type III Wald Chi‐Square tests and resulting Tukey post hoc tests of all tropical fish species' chasing behaviour towards heterospecific tropical fish responses.
**Table S10:** GLMM and Type III Wald Chi‐Square tests of all tropical fish species' chasing behaviour towards conspecific fish responses.
**Table S11:** GLMM, Type III Wald Chi‐Square tests and resulting Tukey post hocs of all tropical fish species' fleeing behaviour from heterospecific tropical fish.
**Table S12:** GLMM and Type III Wald Chi‐Square tests of all tropical fish species' fleeing behaviour from heterospecific temperate fish.
**Table S13:** GLMM, Type III Wald Chi‐Square tests and resulting Tukey post hocs of all tropical fish species' fleeing behaviour from conspecific tropical fish.
**Table S14:** GLMM, Type III Wald Chi‐Square tests and resulting Tukey post hocs of all temperate fish species fleeing behaviour from heterospecific tropical fish.
**Table S15:** GLMM, Type III Wald Chi‐Square tests and resulting Tukey post hocs of all temperate fish species bite rate.
**Table S16:** GLMM and Type III Wald Chi‐Square tests of all temperate fish species' chasing behaviour towards conspecific fish responses.
**Table S17:** GLMM, Type III Wald Chi‐Square tests and resulting Tukey post hocs of all temperate fish species sheltering behaviour.
**Table S18:** GLMM, Type III Wald Chi‐Square tests and resulting Tukey post hocs of all temperate fish species' flight initiation distance responses.
**Table S19:** GLMM and Type III Wald Chi‐Square tests of the focal temperate fish's (*Atypichthys strigatus*) relative lateralization (L_R_) responses.
**Table S20:** GLMM, and Type III Wald Chi‐Square tests of the focal temperate fish's (*Atypichthys strigatus*) absolute lateralization (L_A_) responses.
**Table S21:** GLMM and Type III Wald Chi‐Square tests of all temperate fish species' chasing behaviour towards heterospecific tropical fish responses.
**Table S22:** GLMM, Type III Wald Chi‐Square tests and resulting Tukey post hoc tests of all temperate fish species' chasing behaviour towards heterospecific temperate fish responses.
**Table S23:** GLMM and Type III Wald Chi‐Square tests of all temperate fish species fleeing behaviour from heterospecific temperate fish. Final model selected model presented below uses log (X+1) transformed temperate fish species fleeing behaviour from heterospecific temperate fish data.
**Table S24:** GLMM and Type III Wald Chi‐Square tests of all temperate fish species fleeing behaviour from conspecific temperate fish.
**Figure S1:** Diagram (top view) of tank used for standardised detour test for focal tropical (*Abudefduf vaigiensis*) and focal temperate (*Atypichthys strigatus*) species.
**Figure S2:** Sequence of light initiation distance (FID) test performed on juvenile *A. vaigiensis* in depths of 1.5 metres.
**Figure S3:** DHARMa residual diagnostics for generalized linear mixed models (GLMMs) assessing bite rate (a, b), sheltering behaviour (c, d), relative lateralisation (e, f), absolute lateralisation (g, h), and flight initiation distance (i, j).
**Figure S4:** *DHARMa* residual diagnostics for generalized linear mixed models (GLMMs) assessing chasing heterospecific tropical fishes (a, b), chasing heterospecific temperate fishes (c, d), chasing conspecifics (e, f), fleeing from heterospecific tropical fishes (g; only assessed for the temperate species), fleeing from heterospecific temperate fishes (h; only assessed for the tropical species), and fleeing from conspecifics (i, j).
**Figure S5:** Chasing heterospecific tropical species fish behaviour (chases.sec^−1^) of the focal tropical fish species (A) *Abudefduf bengalensis*, (B) *Abudefduf sexfasciatus*, (C) *Abudefduf vaigiensis*, (D) *Acanthurus nigrofuscus* and (E) *Acanthurus triostegus* in tropical‐only shoals (solid bars) and in mixed‐species shoals (hatched bars), and chasing heterospecific tropical species shoal mate behaviour (chases.sec^−1^) of the focal temperate fish species (F) *Microcanthus joyceae* and (G) *Atypichthys strigatus* in temperate‐only shoals (solid bars) and in mixed‐species shoals (hatched) across regions (TR = Tropical; STR = Subtropical; WT = Warm Temperate, CT = Cold Temperate).
**Figure S6:** Chasing heterospecific temperate species fish behaviour (chases.sec^−1^) of the focal tropical fish species (A) *Abudefduf bengalensis*, (B) *Abudefduf sexfasciatus*, (C) *Abudefduf vaigiensis*, (D) *Acanthurus nigrofuscus* and (E) *Acanthurus triostegus* in tropical‐only shoals (solid bars) and in mixed‐species shoals (hatched bars), and chasing heterospecific temperate species shoal mate behaviour (chases.sec^−1^) of the focal temperate fish species (F) *Microcanthus joyceae* and (G) *Atypichthys strigatus* in temperate‐only shoals (solid bars) and in mixed‐species shoals (hatched) across regions (TR = Tropical; STR = Subtropical; WT = Warm Temperate, CT = Cold Temperate).
**Figure S7:** Fleeing from heterospecific tropical species fish behaviour (flees.sec^−1^) of the focal tropical fish species (A) *Abudefduf bengalensis*, (B) *Abudefduf sexfasciatus*, (C) *Abudefduf vaigiensis*, (D) *Acanthurus nigrofuscus* and (E) *Acanthurus triostegus* in tropical‐only shoals (solid bars) and in mixed‐species shoals (hatched bars), and fleeing from heterospecific tropical species shoal mate behaviour (flees.sec^−1^) of the focal temperate fish species (F) *Microcanthus joyceae* and (G) *Atypichthys strigatus* in temperate‐only shoals (solid bars) and in mixed‐species shoals (hatched) across regions (TR = Tropical; STR = Subtropical; WT = Warm Temperate, CT = Cold Temperate).
**Figure S8:** Fleeing from heterospecific temperate species fish behaviour (flees.sec^−1^) of the focal tropical fish species (A) *Abudefduf bengalensis*, (B) *Abudefduf sexfasciatus*, (C) *Abudefduf vaigiensis*, (D) *Acanthurus nigrofuscus* and (E) *Acanthurus triostegus* in tropical‐only shoals (solid bars) and in mixed‐species shoals (hatched bars), and fleeing from heterospecific temperate species shoal mate behaviour (flees.sec^−1^) of the focal temperate fish species (F) *Microcanthus joyceae* and (G) *Atypichthys strigatus* in temperate‐only shoals (solid bars) and in mixed‐species shoals (hatched) across regions (TR = Tropical; STR = Subtropical; WT = Warm Temperate, CT = Cold Temperate).
**Figure S9:** Antagonistic chasing conspecific shoal fish behaviour (chase.sec^−1^) of the focal tropical fish species (A) *Abudefduf bengalensis*, (B) *Abudefduf sexfasciatus*, (C) *Abudefduf vaigiensis*, (D) *Acanthurus nigrofuscus* and (E) *Acanthurus triostegus* in tropical‐only shoals (solid bars) and in mixed‐species shoals (hatched bars), and antagonistic chasing conspecific shoal mate behaviour (chase.sec^−1^) of the focal temperate fish species (F) *Microcanthus joyceae* and (G) *Atypichthys strigatus* in temperate‐only shoals (solid bars) and in mixed‐species shoals (hatched) across regions (TR = Tropical; STR = Subtropical; WT = Warm Temperate, CT = Cold Temperate).
**Figure S10:** Fleeing from conspecific fish behaviour (flees.sec^−1^) of the focal tropical fish species (A) *Abudefduf bengalensis*, (B) *Abudefduf sexfasciatus*, (C) *Abudefduf vaigiensis*, (D) *Acanthurus nigrofuscus* and (E) *Acanthurus triostegus* in tropical‐only shoals (solid bars) and in mixed‐species shoals (hatched bars), and fleeing from conspecific shoal mate behaviour (flees.sec^−1^) of the focal temperate fish species (F) *Microcanthus joyceae* and (G) *Atypichthys strigatus* in temperate‐only shoals (solid bars) and in mixed‐species shoals (hatched) across regions (TR = Tropical; STR = Subtropical; WT = Warm Temperate, CT = Cold Temperate).

## Data Availability

Data are available from Figshare data repository https://figshare.com/s/02562cf2ee876b9a6745 (Mitchell et al., 2025).
