# Supplementary material for: Tropical fishes can benefit more from novel than familiar species interactions at their cold‐range edges
Source: J Anim Ecol. 2025 Jul 23;94(10):1997–2010. doi: 10.1111/1365-2656.70100 (PMC12484409; doi:10.1111/1365-2656.70100)
Supplement: Supplementary file 1 — Table S1: Ethogram of behaviours and aggressive interactions measured from video recordings record across regions and shoal types sampled. Table S2: Mean summer seawater temperatures (°C) collected using HOBO temperature recorders at the same time as the recording of fish behaviours during 2017 and 2018. Table S3: GLMM, Type III Wald Chi‐Square tests and resulting Tukey post hoc tests of all tropical fish species bite rate. Table S4: GLMM, Type III Wald Chi‐Square tests and resulting Tukey post hocs of all tropical fish species' sheltering behaviour. Table S5: GLMM, Type III Wald Chi‐Square tests and resulting Tukey post hoc tests of all tropical fish species' chasing behaviour towards heterospecific temperate fish responses. Table S6: GLMM, Type III Wald Chi‐Square tests and resulting Tukey post hoc tests of the focal tropical fish's (Abudefduf vaigiensis) relative lateralization (LR) responses. Table S7: GLMM, Type III Wald Chi‐Square tests and resulting Tukey post hoc tests of the focal tropical fish's (Abudefduf vaigiensis) absolute lateralization (LA) responses. Table S8: GLMM, Type III Wald Chi‐Square tests and resulting Tukey post hocs of all tropical fish species' flight initiation distance responses. Table S9: GLMM, Type III Wald Chi‐Square tests and resulting Tukey post hoc tests of all tropical fish species' chasing behaviour towards heterospecific tropical fish responses. Table S10: GLMM and Type III Wald Chi‐Square tests of all tropical fish species' chasing behaviour towards conspecific fish responses. Table S11: GLMM, Type III Wald Chi‐Square tests and resulting Tukey post hocs of all tropical fish species' fleeing behaviour from heterospecific tropical fish. Table S12: GLMM and Type III Wald Chi‐Square tests of all tropical fish species' fleeing behaviour from heterospecific temperate fish. Table S13: GLMM, Type III Wald Chi‐Square tests and resulting Tukey post hocs of all tropical fish species' fleeing behaviour from conspecific tropical fish. Tabl [file JANE-94-1997-s001.docx]

**Supplementary Information for: Tropical fishes can benefit more from novel than familiar species interactions at their cold-range edges**

36 cm

6 cm

20 cm

24 cm

**Figure S1:**  Diagram (top view) of tank used for standardised detour test for focal tropical (Abudefduf vaigiensis) and focal temperate (Atypichthys strigatus) species. Not to scale. The hatched areas represent runway barriers, and the arrows represent the directional change a fish may take when fleeing from the perceived threat.


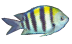


**Table S1:** Ethogram of behaviours and aggressive interactions measured from video recordings record across regions and shoal types sampled.

| **Behaviours** | **Measure** | **Definition** |
| --- | --- | --- |
| Foraging | Bite Rate (Bites.sec^-1^) | Focal fish bites unsuccessfully or successfully at substrate or in the water column at potential food item. |
| Sheltering | Time spent sheltering (%) | Focal fish spends time in sheltering habitat (e.g. overhang, ledge, between rocky substrate) unexposed and not in the water column. Focal fish was consider sheltering when at least some part of their body was covered by sheltering habitat |
| Flight Initiation Distance | Flight initiation distance (cm) | The distance at which a fish initiates an escape response towards an artificial threat. |
| **Aggressive interactions** | **Measure** | **Definition** |
| Chasing | Interactions.sec^-1^ | Focal fish chases, bites, or flares of fins towards shoal mate (can be either heterospecific tropical, heterospecific temperate fish or a conspecific shoal mate) |
| Fleeing | Interactions.sec^-1^ | Either a heterospecific tropical, heterospecific temperate or conspecific shoal mate chases, bites, or flares fins towards focal fish |


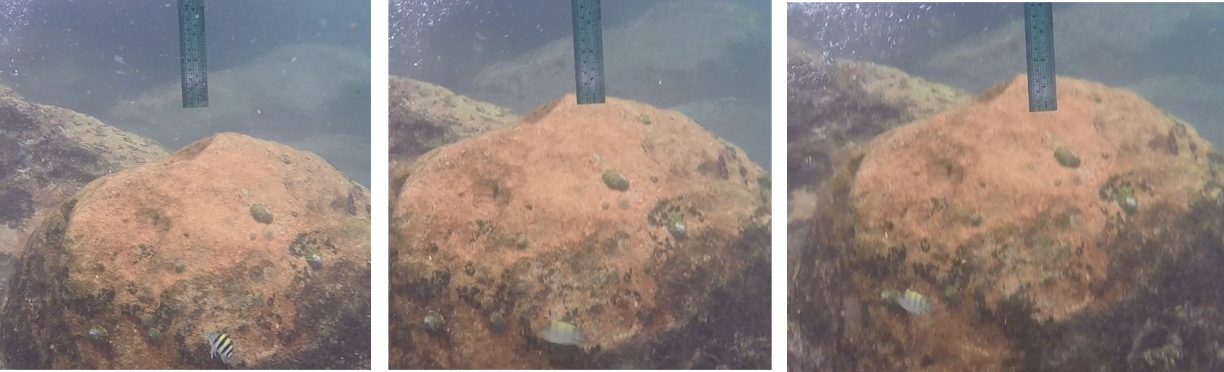


**(A)**

**(B)**

**(C)**

**Figure S2:** Sequence of light initiation distance (FID) test performed on juvenile *A. vaigiensis* in depths of 1.5 metres. Panel (A) shows the ruler over the head of the juvenile fish*.* Panel (B) shows the flight initiation distance of the juvenile fish and panel (C) shows the elicited escape responses of the juvenile fish.

**Table S2:** Mean summer seawater temperatures (°C) collected using HOBO temperature recorders at the same time as the recording of fish behaviours during 2017 and 2018. Behaviour data from the tropical locations was only collected in 2021 in April (Heron Island) and June (Magnetic Island), with water temperatures recorded using a dive watch for all sampling regions in 2021.

| Region | Location | 2017 | 2018 | 2021 |
| --- | --- | --- | --- | --- |
| Tropical | Heron Island | - | - | 25.8 |
|  | Magnetic Island | - | - | 22.4 |
| Subtropical | Tweed Heads | - | - | 22.6 |
|  | South West Rocks | 25.4 | 25.7 | 22.8 |
| Warm Temperate | Sydney | 23.0 | 23.0 | 20.6 |
| Cold Temperate | Narooma | 21.9 | 21.4 | 21.6 |
|  | Merimbula | 21.2 | 20.1 | 21.6 |

**Flight Initiation Distance**

**Absolute Lateralization**

**Relative Lateralization**

**Sheltering Behaviour**

**Bite Rate**


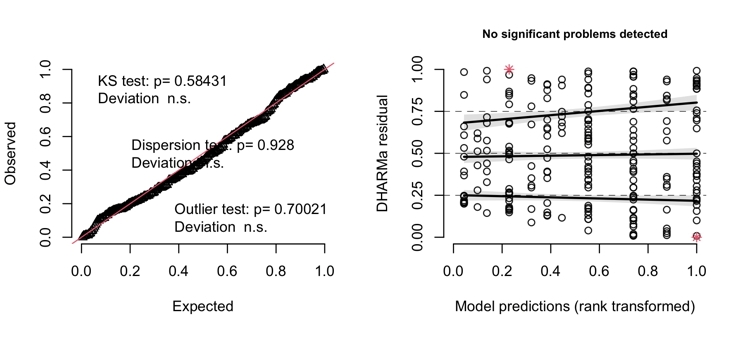


**Tropical Fish Species**

**Temperate Fish Species**


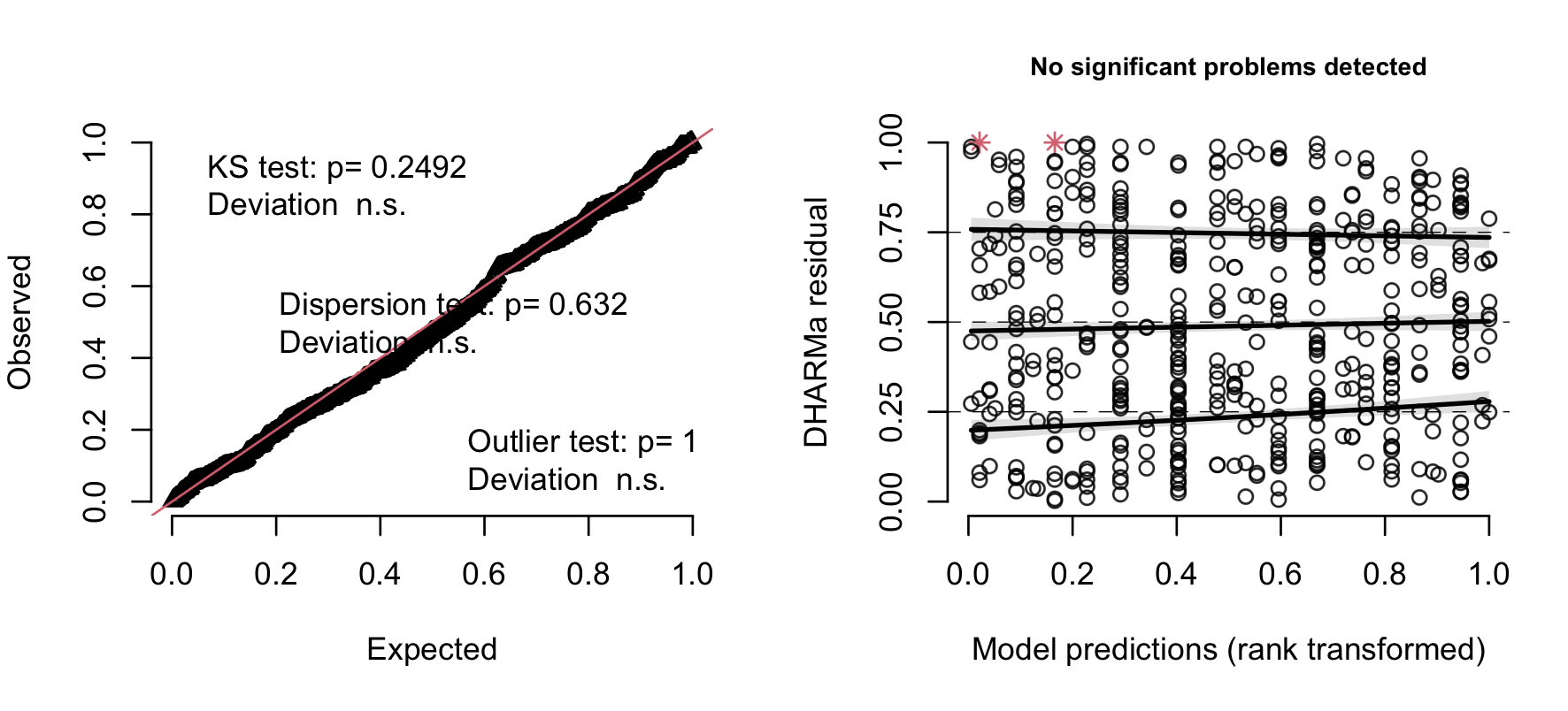

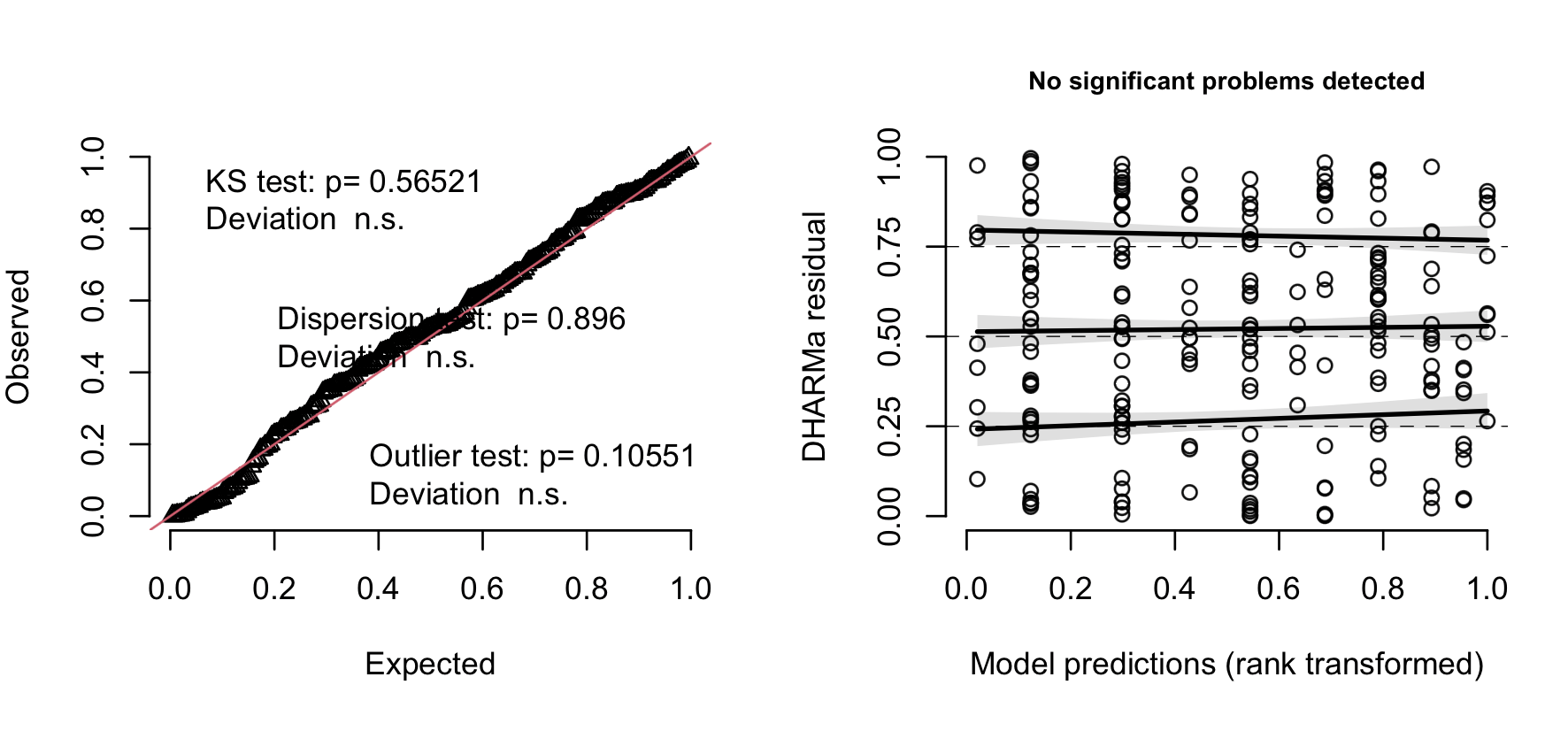

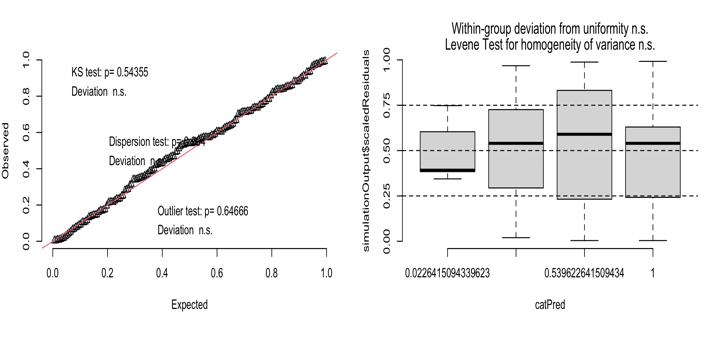

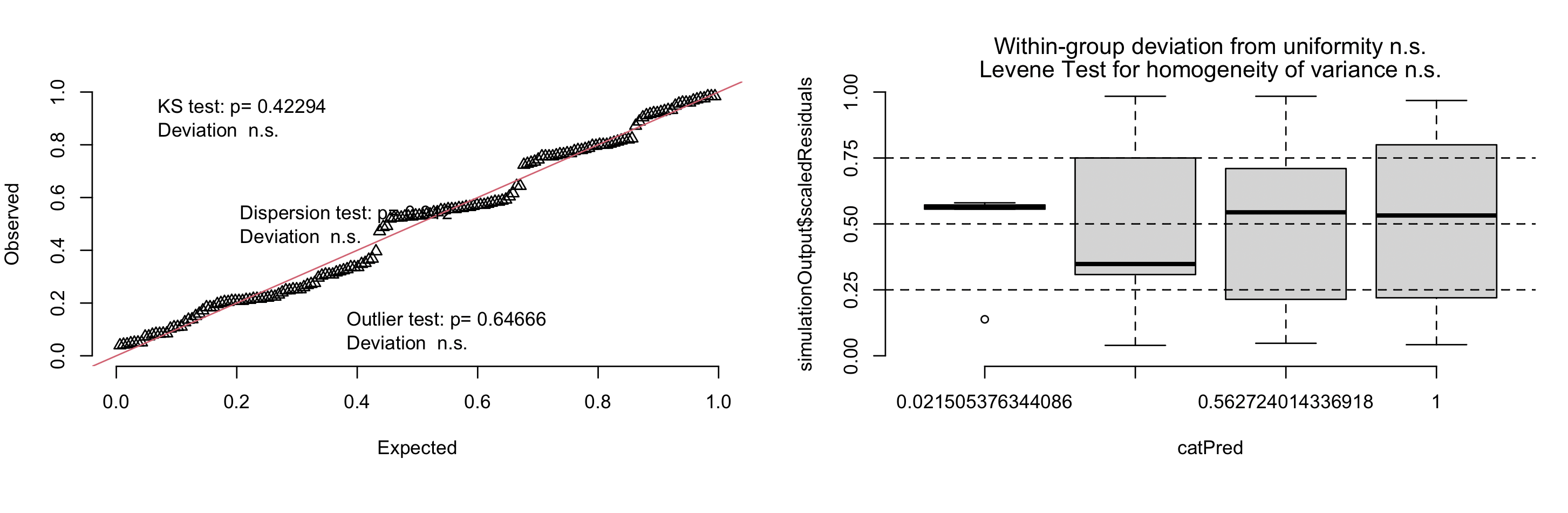

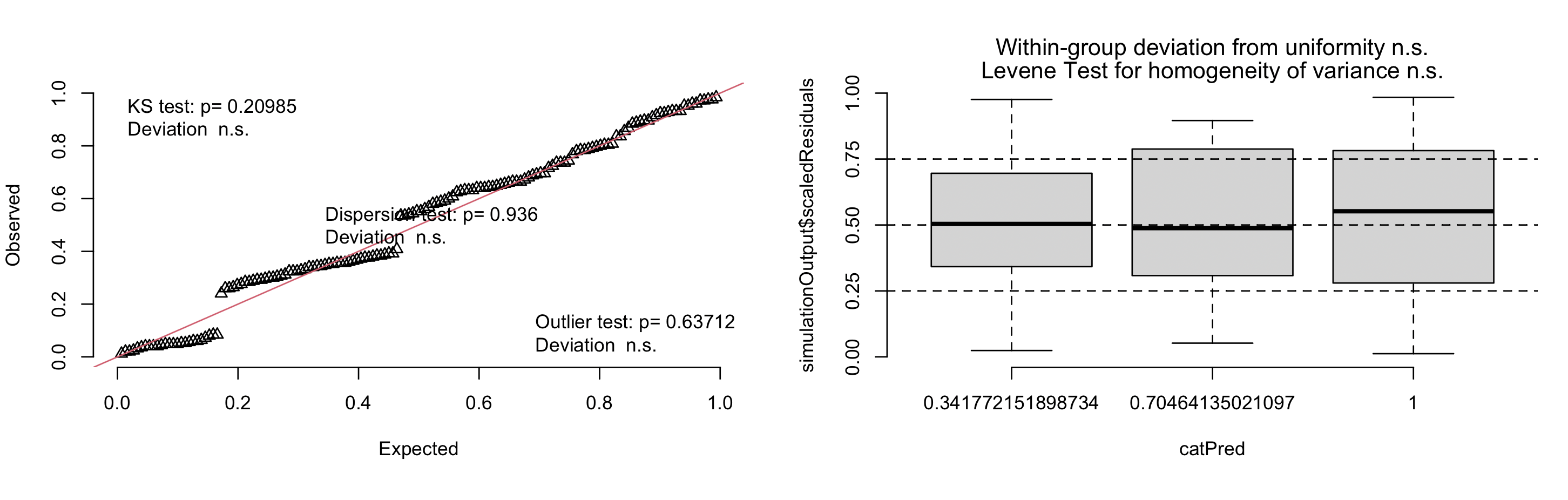

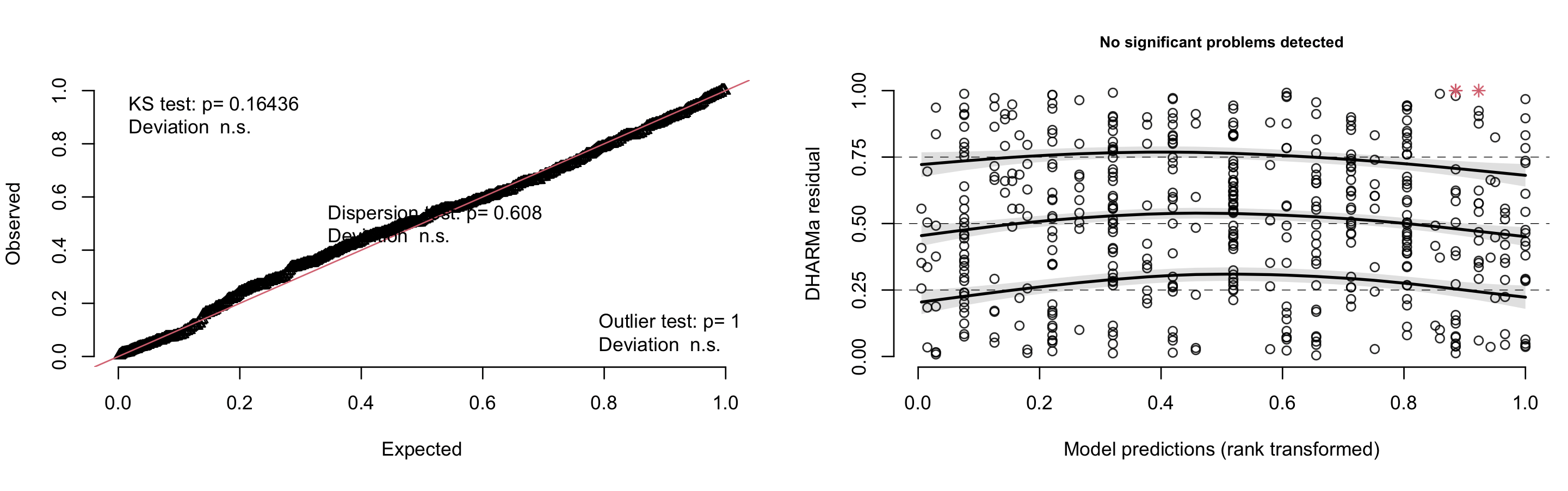

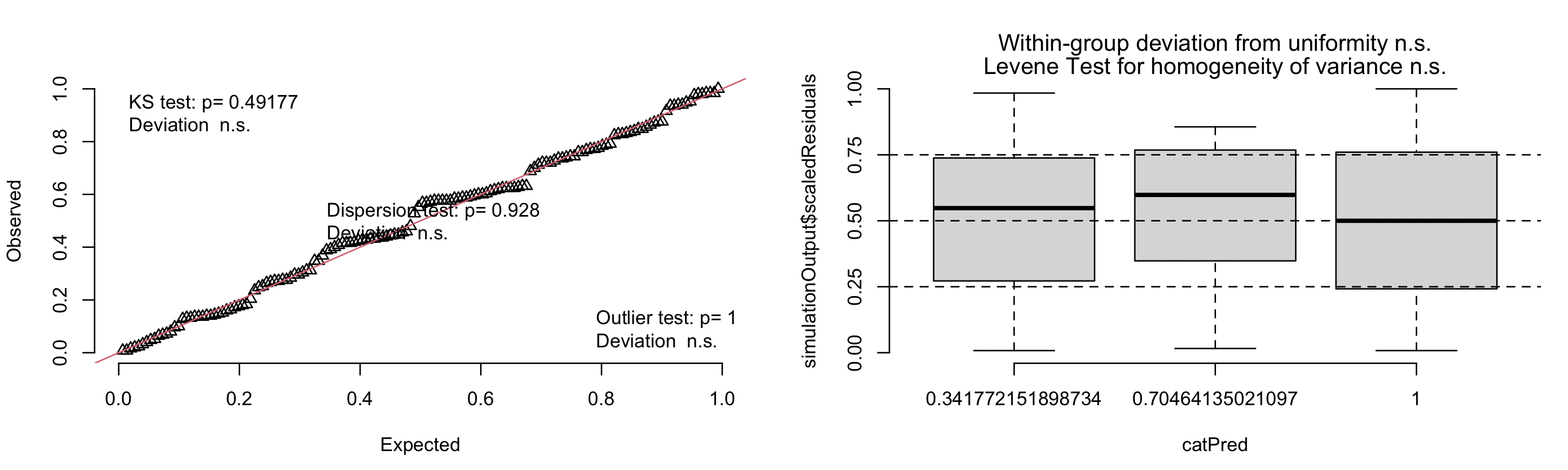

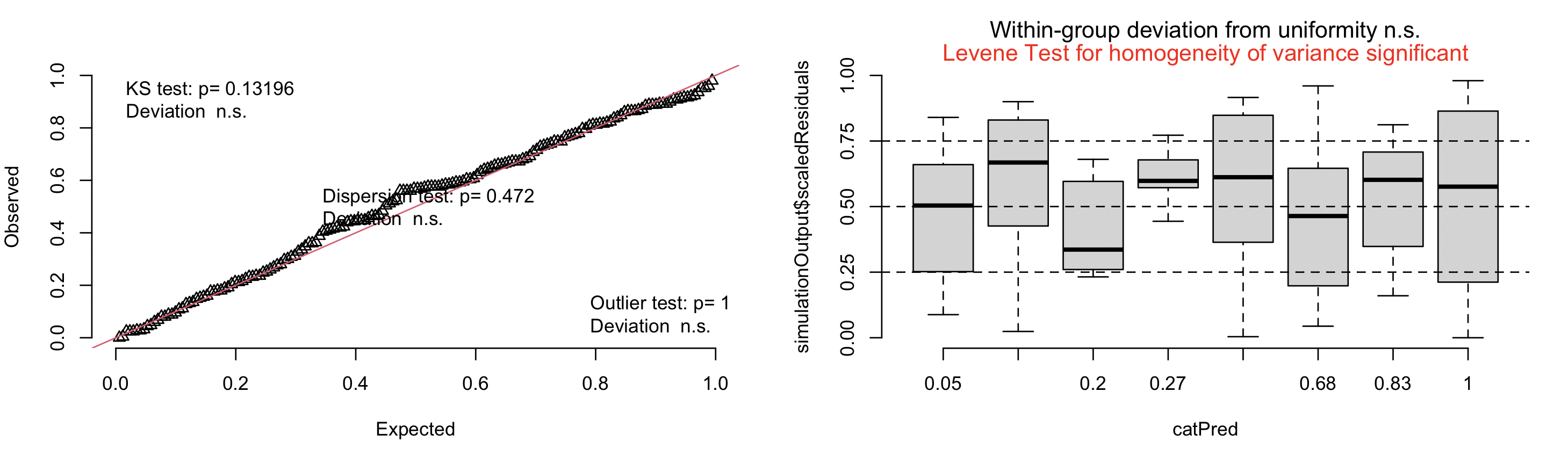

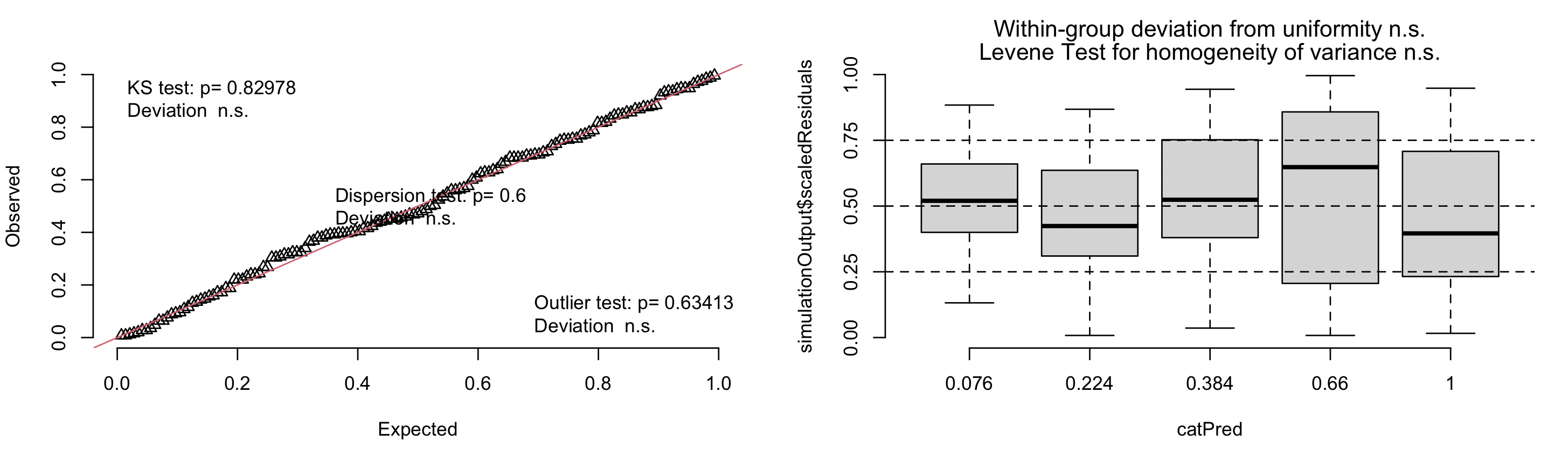


**(a)**

**(b)**

**(c)**

**(d)**

**(e)**

**(f)**

**(g)**

**(h)**

**(i)**

**(j)**

**Figure S3:** DHARMa residual diagnostics for generalized linear mixed models (GLMMs) assessing bite rate (a, b), sheltering behaviour (c, d), relative lateralisation (e, f), absolute lateralisation (g, h), and flight initiation distance (i, j). Each row corresponds to a different behavioural response variable. The left panels show Q-Q plots of residuals, assessing normality using the **Kolmogorov-Smirnov (KS) test**. The middle panels display residuals vs. fitted plots, used to assess homoscedasticity and potential structure in residuals. The right panels present boxplots of scaled residuals by region, examining variance homogeneity using **Levene’s test**. Additional model diagnostics include the **dispersion test**, assessing whether the variance structure is appropriate; the **outlier test**, detecting extreme residual values; and the **quantile test**, evaluating deviations from expected quantiles. A significant violation of homogeneity of variance was detected for the tropical fish species’ flight initiation distance GLMM (**Levene’s test, p < 0.05**), while all other models in Figure S3 met GLMM assumptions. The left column of DHARMa outputs corresponds to tropical species models, while the right column represents temperate species models.

**Fleeing Conspecifics**

**Chasing Conspecifics**


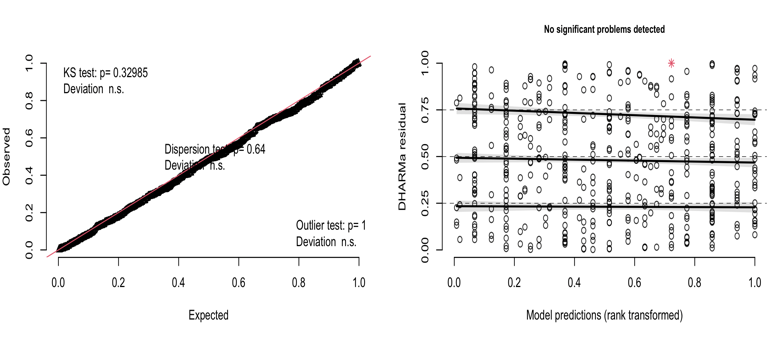

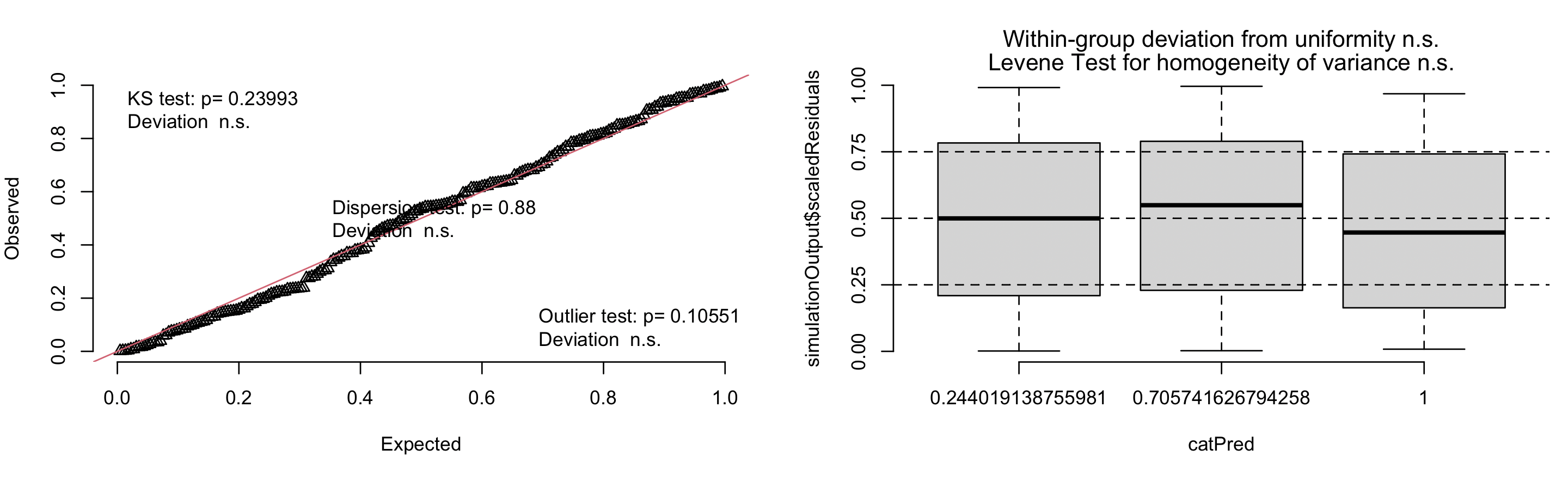


**Chasing Heterospecific Tropical Fishes**


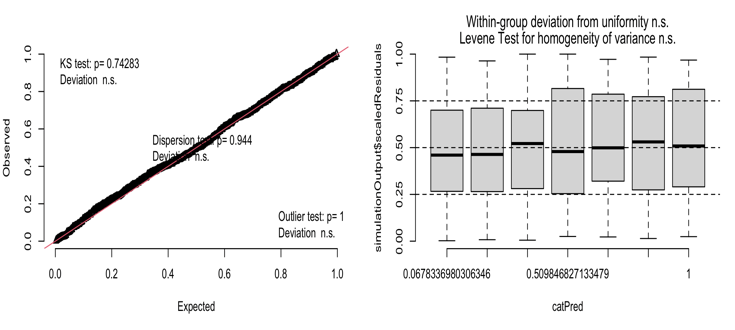


**Chasing Heterospecific Temperate Fishes**


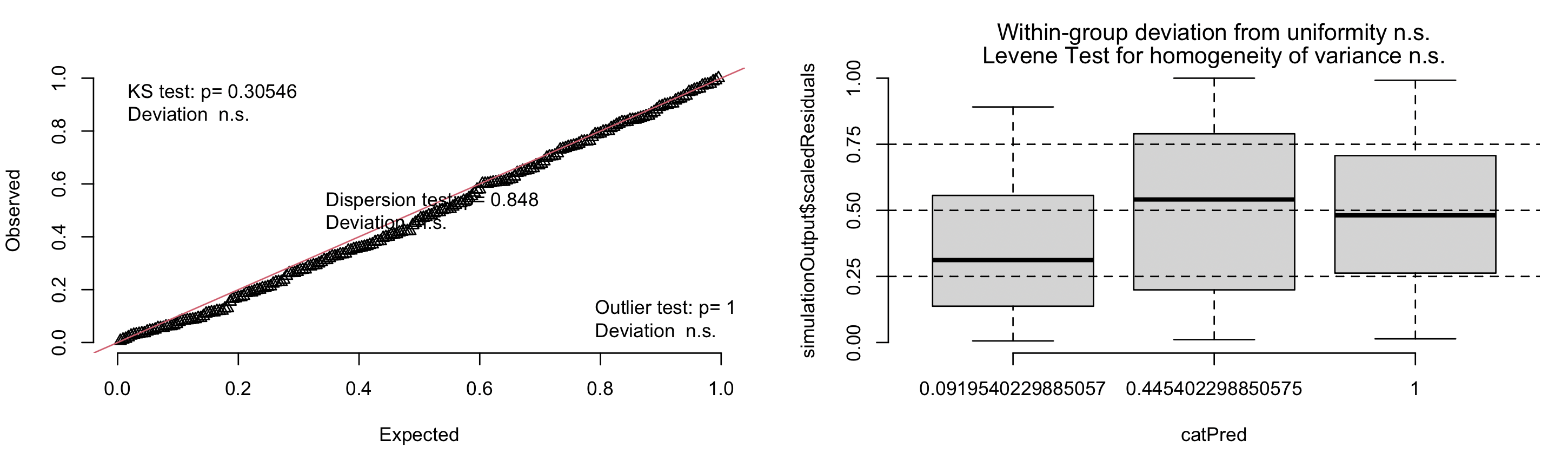

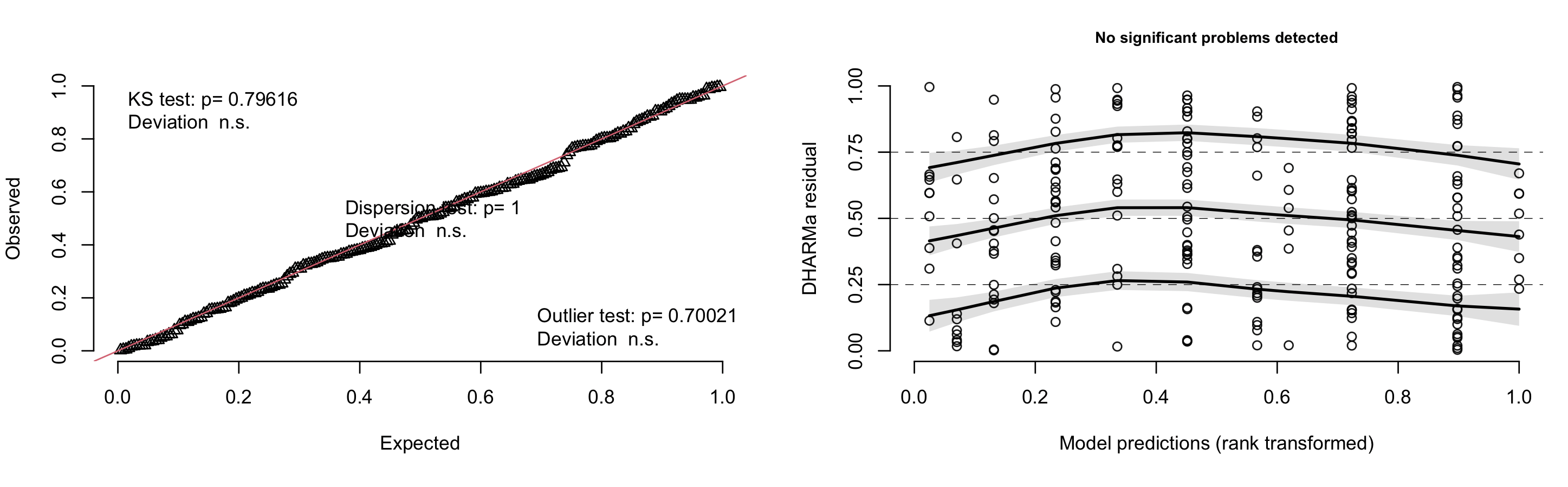


**Fleeing Tropical Heterospecific Fishes**


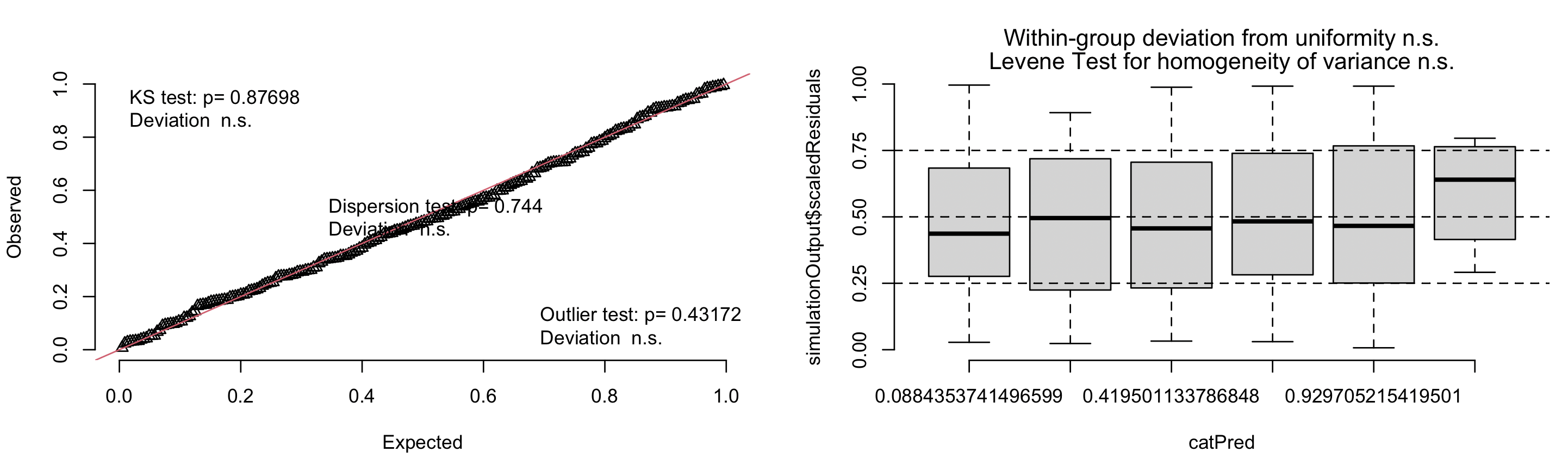


**Fleeing Temperate Heterospecific Fishes**


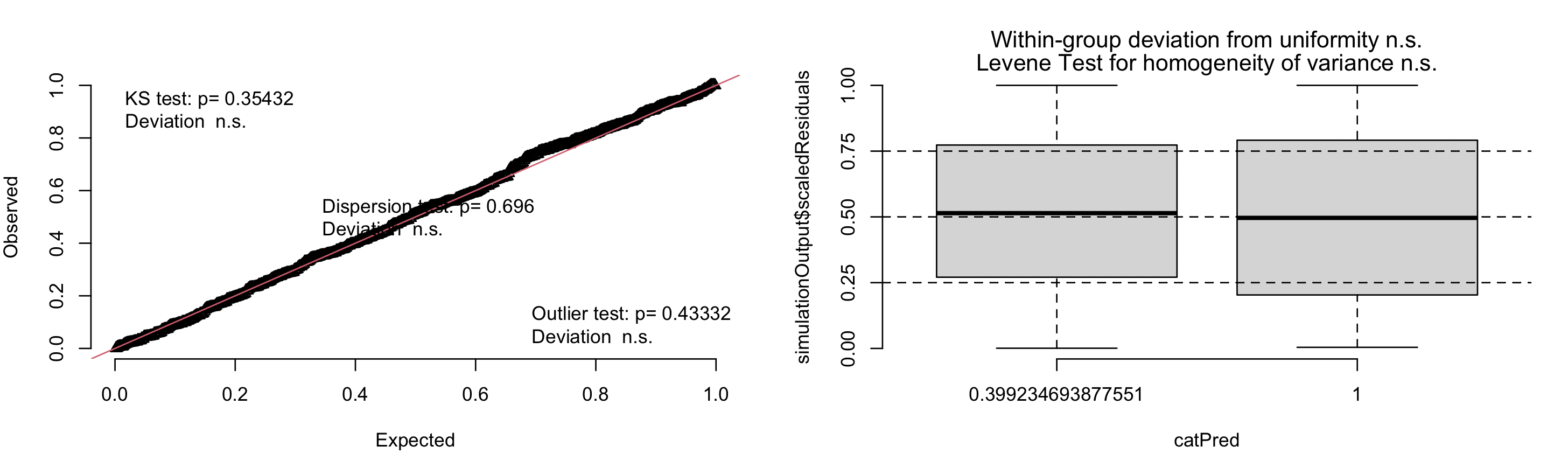

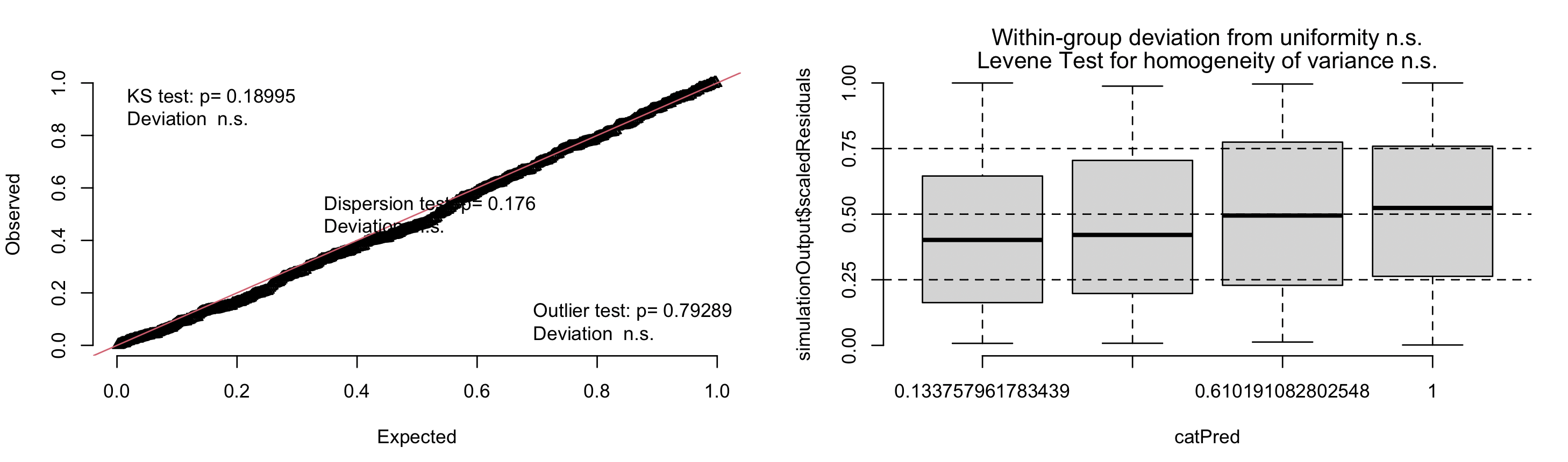

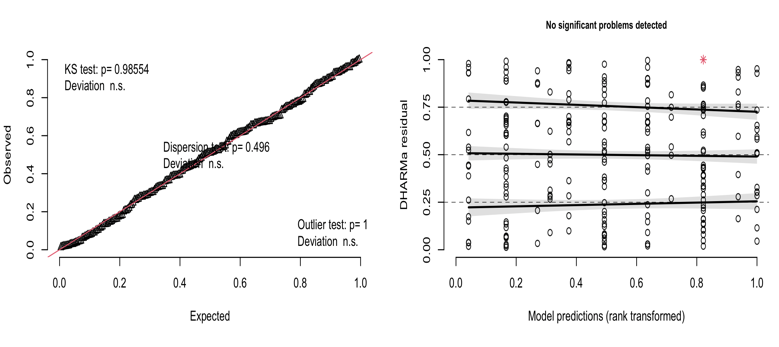


**(a)**

**(b)**

**(c)**

**(d)**

**(e)**

**(f)**

**(g)**

**(h)**

**(i)**

**(j)**


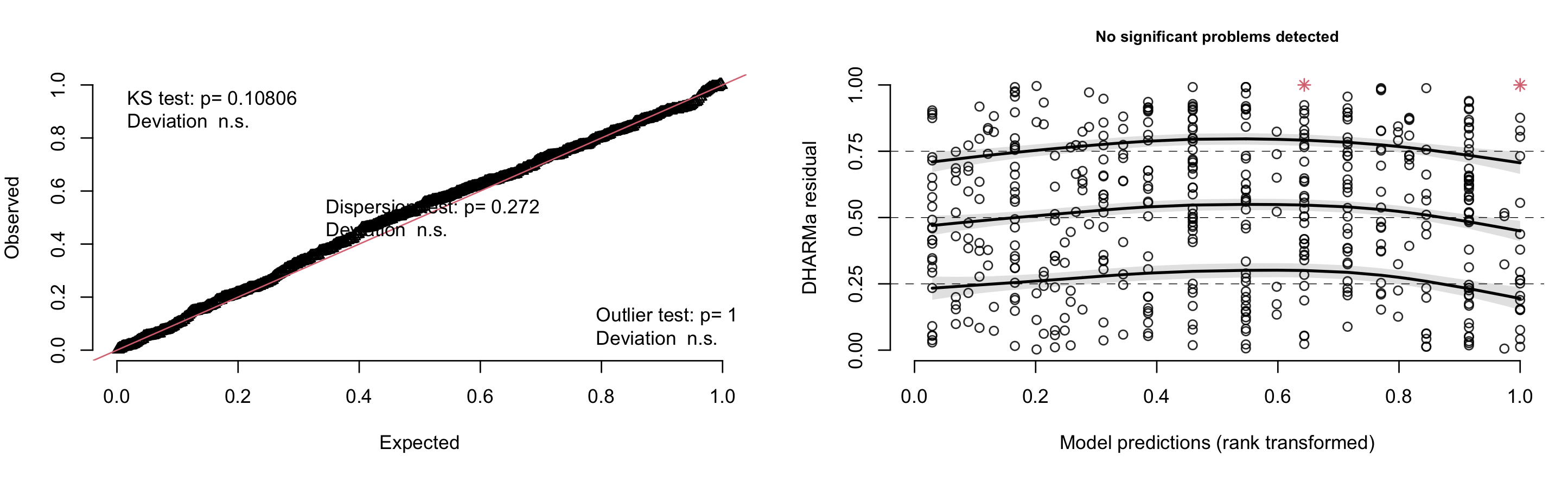


**Figure S4:** *DHARMa* residual diagnostics for generalized linear mixed models (GLMMs) assessing chasing heterospecific tropical fishes (a, b), chasing heterospecific temperate fishes (c, d), chasing conspecifics (e, f), fleeing from heterospecific tropical fishes (g; only assessed for the temperate species), fleeing from heterospecific temperate fishes (h; only assessed for the tropical species), and fleeing from conspecifics (i, j). Each row corresponds to a different behavioural response variable. The left panels show Q-Q plots of residuals, assessing normality using the Kolmogorov-Smirnov (KS) test. The middle panels display residuals vs. fitted plots, used to assess homoscedasticity and potential structure in residuals. The right panels present boxplots of scaled residuals by region, examining variance homogeneity using Levene’s test. Additional model diagnostics include the dispersion test, assessing whether the variance structure is appropriate; the outlier test, detecting extreme residual values; and the quantile test, evaluating deviations from expected quantiles. For the interaction models assessing tropical fish fleeing heterospecific tropical fishes and temperate fish fleeing heterospecific tropical fishes, the null model was retained as the best-fit model based on AIC rankings, and no *DHARMa* residual diagnostics were computed for the null model. The left column of *DHARMa* outputs corresponds to tropical species models, while the right column represents temperate species models.


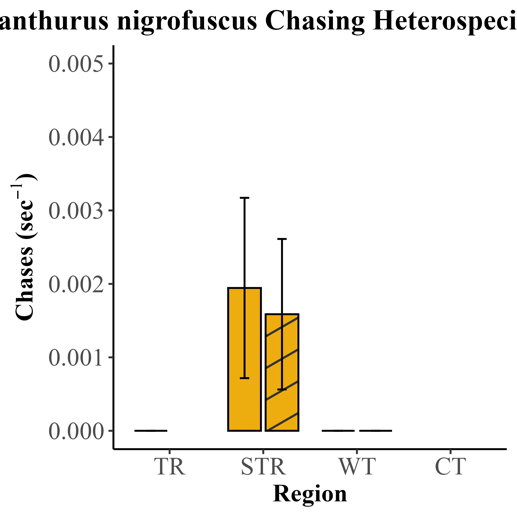

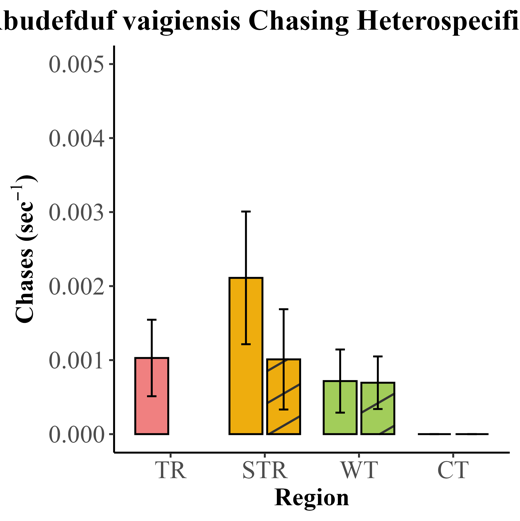

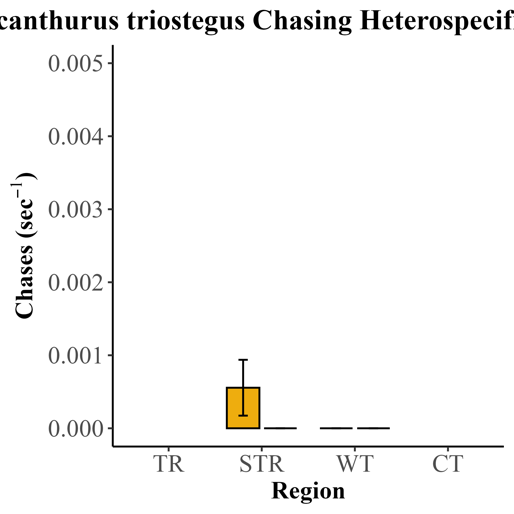

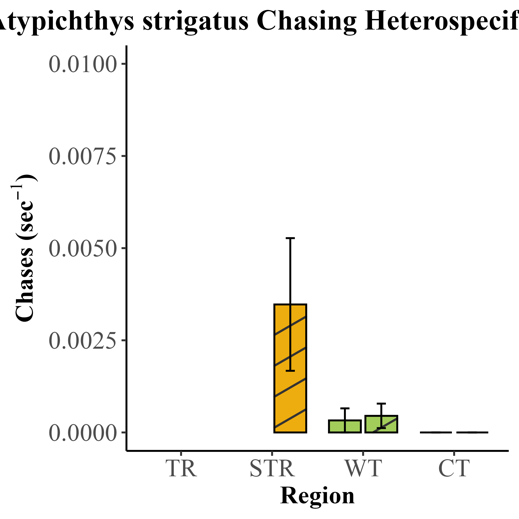

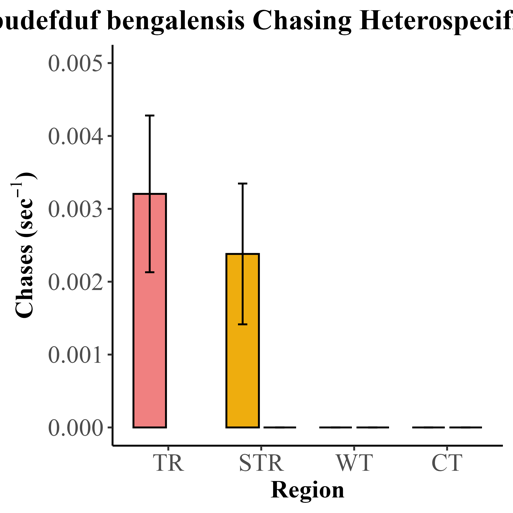

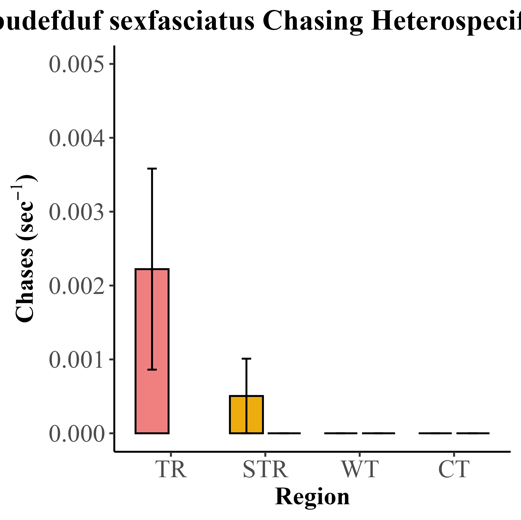

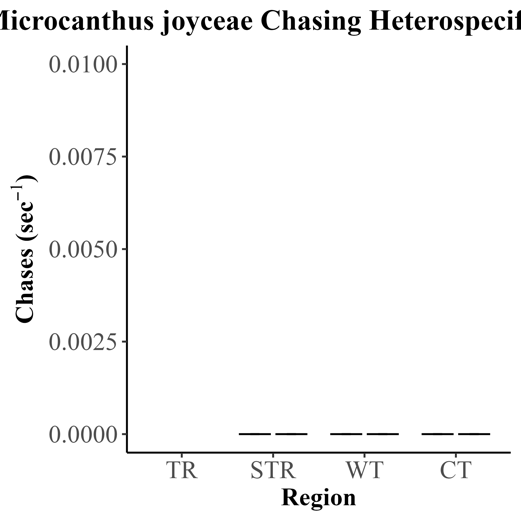

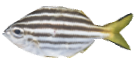

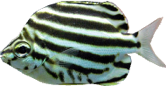

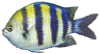

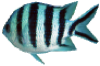

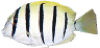

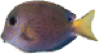


**(A)**

**(B)**

**(C)**

**(D)**

**(E)**

**(F)**

**(G)**


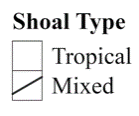


**Region**


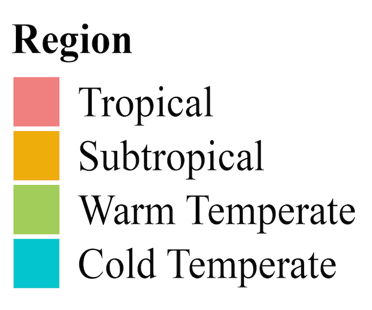


**Chasing Heterospecific Tropical Fish**


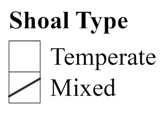


**N/A**

**N/A**

**N/A**

**N/A**

**N/A**

**N/A**

**N/A**

**N/A**

**N/A**

**N/A**

**0**

**0**

**0**

**0**

**0**

**0**

**0**

**0**

**0**

**0**

**0**

**0**

**0**

**0**

**0**

**0**

**0**

**0**

**0**

**0**

**0**

**0**

**0**

**0**

**0**


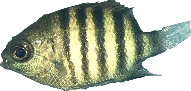


**NS**

**NS**

**Figure S5:** Chasing heterospecific tropical species fish behaviour (chases.sec^-1^) of the focal tropical fish species (A) *Abudefduf bengalensis,* (B) *Abudefduf sexfasciatus,* (C) *Abudefduf vaigiensis,* (D) *Acanthurus nigrofuscus* and (E) *Acanthurus triostegus*  in tropical-only shoals (solid bars) and in mixed-species shoals (hatched bars), and chasing heterospecific tropical species shoal mate behaviour (chases.sec^-1^) of the focal temperate fish species (F) *Microcanthus joyceae* and (G) *Atypichthys strigatus*  in temperate-only shoals (solid bars) and in mixed-species shoals (hatched) across regions (TR = Tropical; STR = Subtropical; WT = Warm Temperate, CT = Cold Temperate). NS = not significant (P > 0.05). N/A denotes that fish species was not sampled in specified shoal type within a region or that focal species was not sampled within specific region. Corresponding statistical outputs are in Tables S9 and S21.

**Region**

**0**

**Temperate Fish Species**

**Tropical Fish Species**


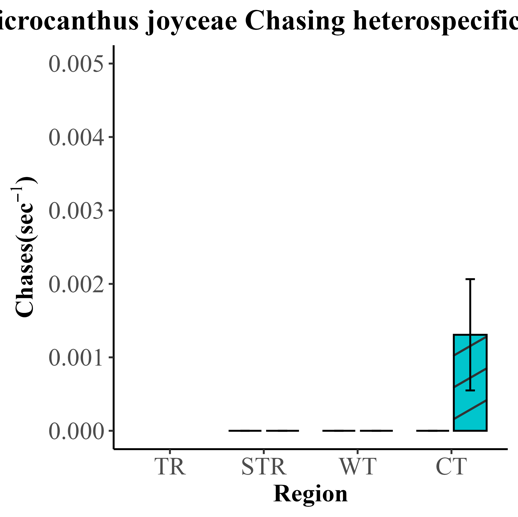

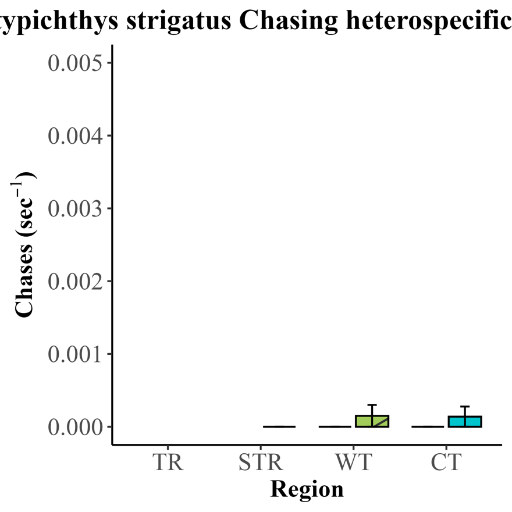

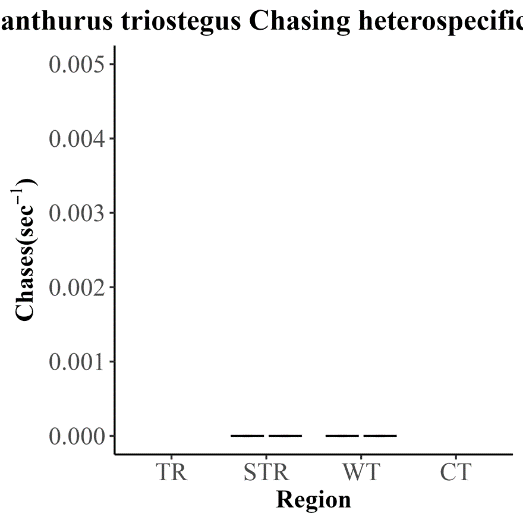

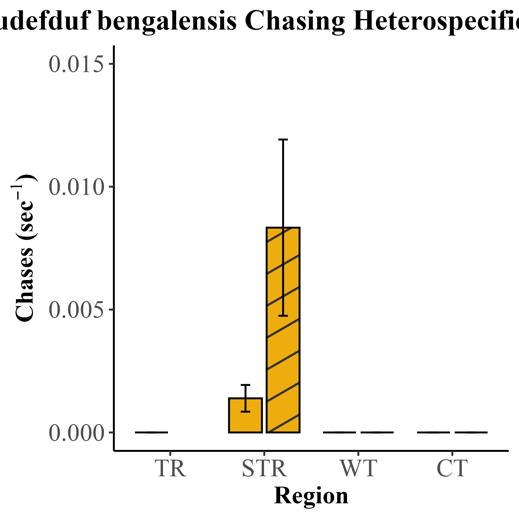

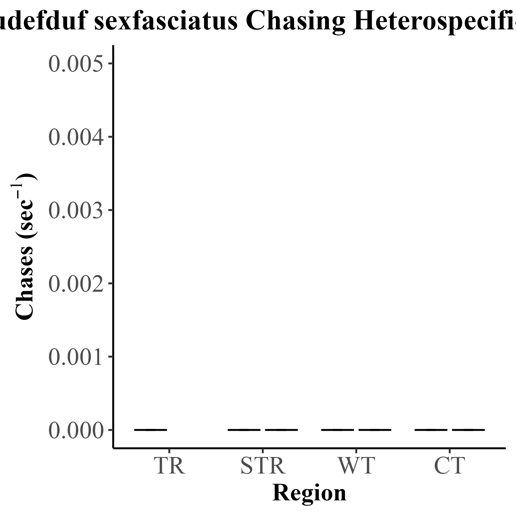

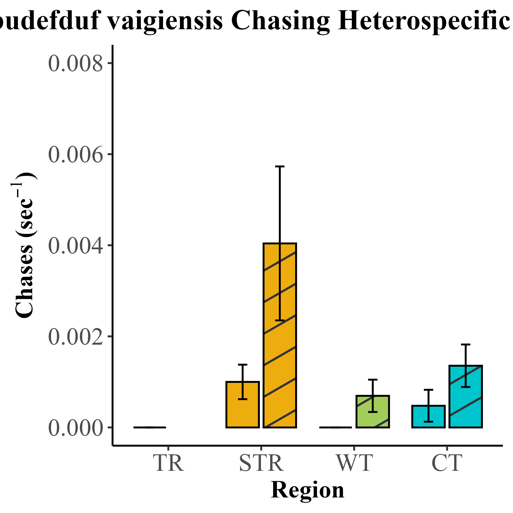

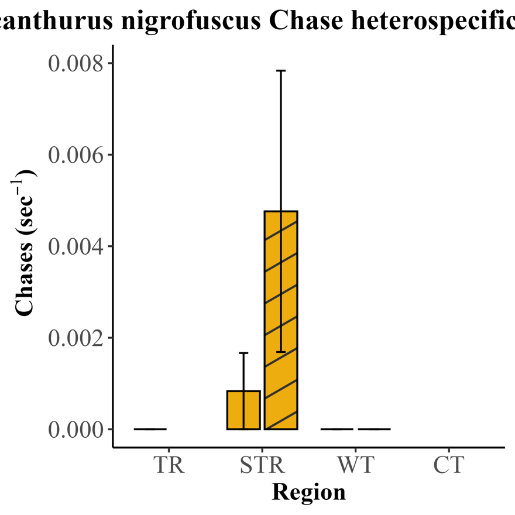


**Region**


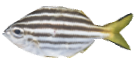

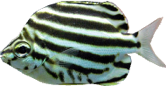

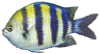

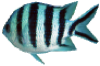

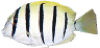

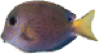


**(A)**

**(B)**

**(C)**

**(D)**

**(E)**

**(F)**

**(G)**


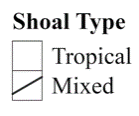


**Temperate Fish Species**

**Tropical Fish Species**


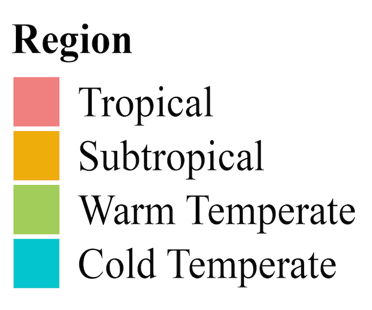


**Chasing Heterospecific Temperate Fish**

**Region**


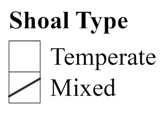


**N/A**

**N/A**

**N/A**

**N/A**

**N/A**

**N/A**

**N/A**

**N/A**

**N/A**

**N/A**

**0**

**0**

**0**

**0**

**0**

**0**

**0**

**0**

**0**

**0**

**0**

**0**

**0**

**0**

**0**

**0**

**0**

**0**

**0**

**0**

**0**

**0**

**0**

**0**

**0**

**0**

**0**

**0**

**0**

**Figure S6:** Chasing heterospecific temperate species fish behaviour (chases.sec^-1^) of the focal tropical fish species (A) *Abudefduf bengalensis,* (B) *Abudefduf sexfasciatus,* (C) *Abudefduf vaigiensis,* (D) *Acanthurus nigrofuscus* and (E) *Acanthurus triostegus*  in tropical-only shoals (solid bars) and in mixed-species shoals (hatched bars), and chasing heterospecific temperate species shoal mate behaviour (chases.sec^-1^) of the focal temperate fish species (F) *Microcanthus joyceae* and (G) *Atypichthys strigatus*  in temperate-only shoals (solid bars) and in mixed-species shoals (hatched) across regions (TR = Tropical; STR = Subtropical; WT = Warm Temperate, CT = Cold Temperate). NS = not significant (P > 0.05). N/A denotes that fish species was not sampled in specified shoal type within a region or that focal species was not sampled within specific region. Corresponding statistical outputs are in Tables S5 and S22.


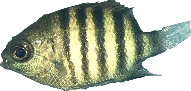


**NS**

**Region: P = 0.004
Shoal Type: P < 0.001**

**a**

**a**

**a**

**a**

**ab**

**ab**

**ab**

**ab**

**ab**

**b**

**b**


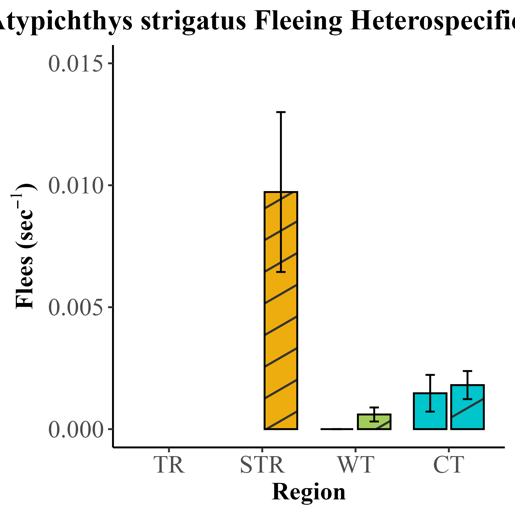

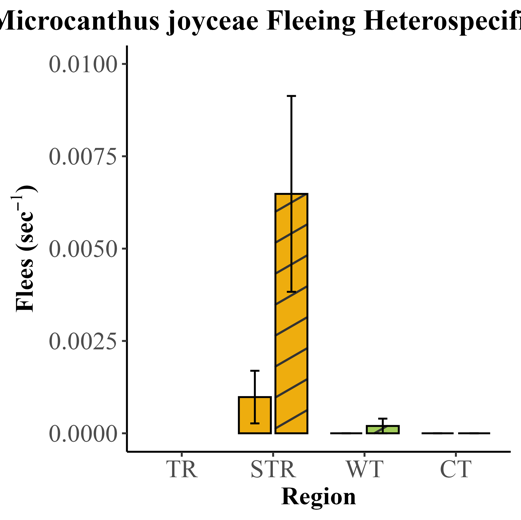

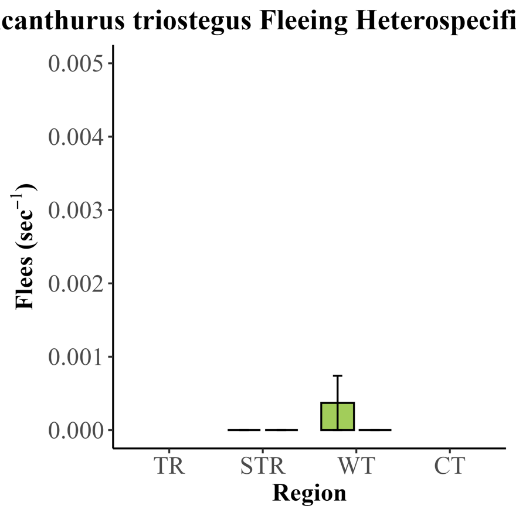

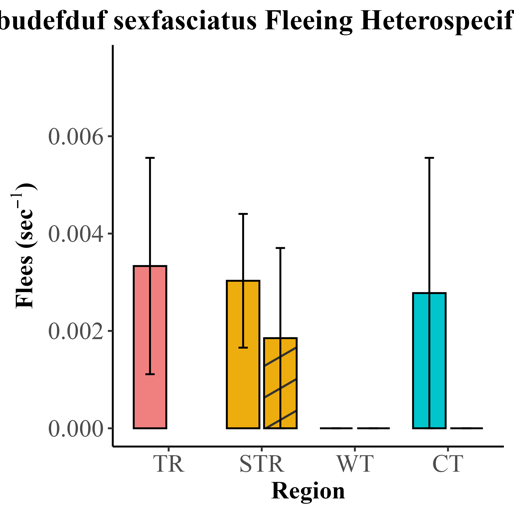

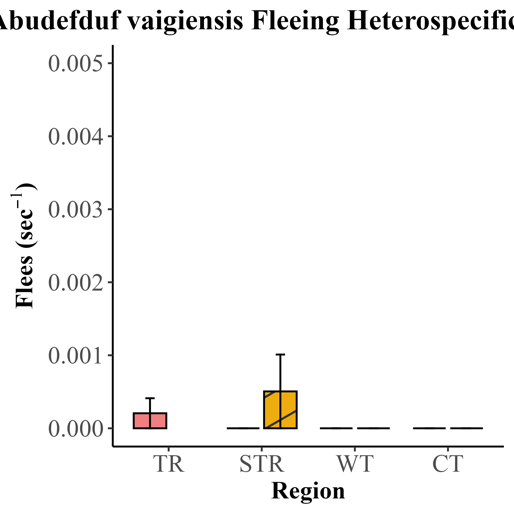

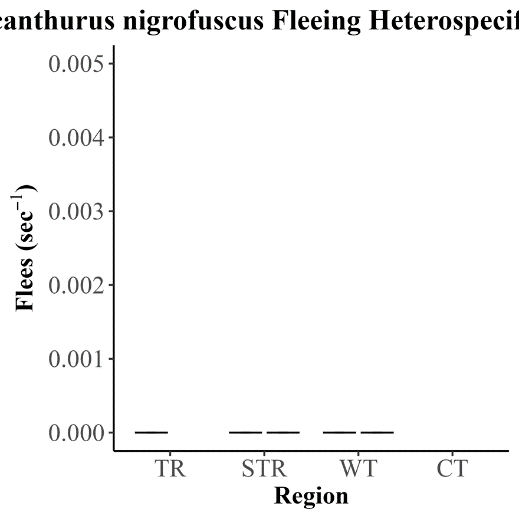

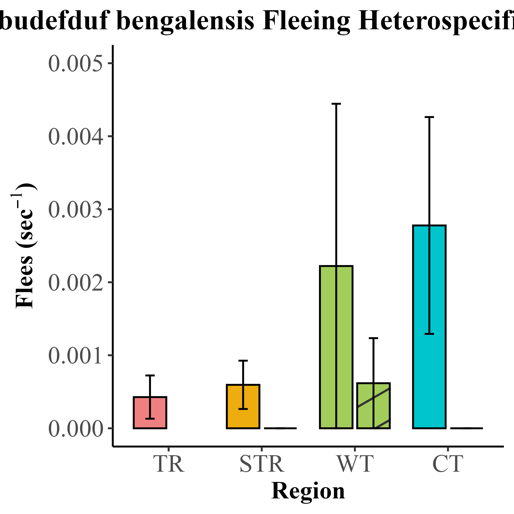


**Region**


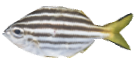

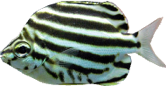

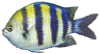

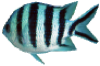

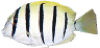

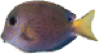


**(A)**

**(B)**

**(C)**

**(D)**

**(E)**

**(F)**

**(G)**


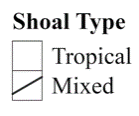


**Temperate Fish Species**

**Tropical Fish Species**


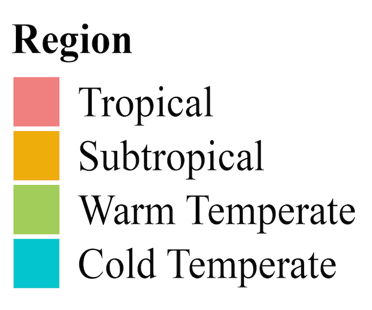


**Fleeing Heterospecific Tropical Fish**

**Region**


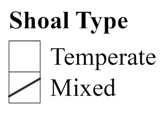


**0**

**0**

**0**

**0**

**0**

**0**

**0**

**0**

**0**

**0**

**0**

**0**

**0**

**0**

**0**

**0**

**0**

**0**

**0**

**0**

**0**

**0**

**N/A**

**N/A**

**N/A**

**N/A**

**N/A**

**N/A**

**N/A**

**N/A**

**N/A**

**N/A**

**Figure S7:** Fleeing from heterospecific tropical species fish behaviour (flees.sec^-1^) of the focal tropical fish species (A) *Abudefduf bengalensis,* (B) *Abudefduf sexfasciatus,* (C) *Abudefduf vaigiensis,* (D) *Acanthurus nigrofuscus* and (E) *Acanthurus triostegus*  in tropical-only shoals (solid bars) and in mixed-species shoals (hatched bars), and fleeing from heterospecific tropical species shoal mate behaviour (flees.sec^-1^) of the focal temperate fish species (F) *Microcanthus joyceae* and (G) *Atypichthys strigatus*  in temperate-only shoals (solid bars) and in mixed-species shoals (hatched) across regions (TR = Tropical; STR = Subtropical; WT = Warm Temperate, CT = Cold Temperate). Different letters above bars indicate significant differences among regions P < 0.05; Table S11, S14). Error bars represent standard errors. Null = null model retained. N/A denotes that fish species was not sampled in specified shoal type within a region or that focal species was not sampled within specific region.


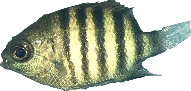


**Null**

**Region: P < 0.001
Species: P = 0.001**

**a**

**a**

**ab**

**ab**


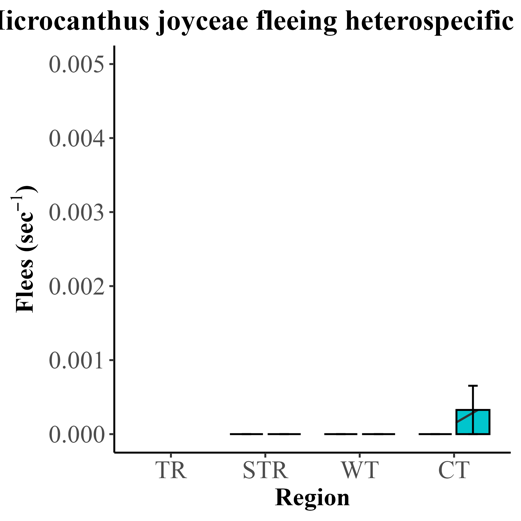

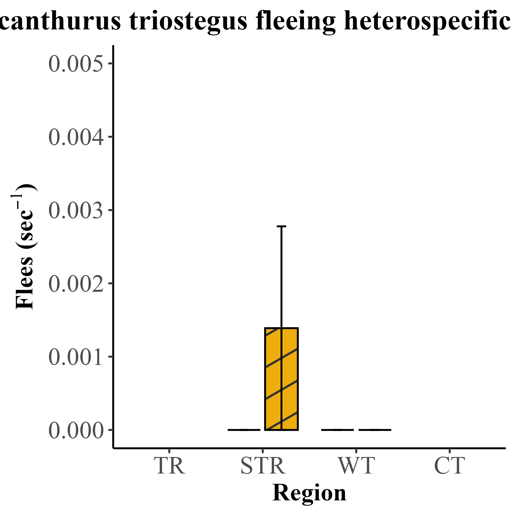

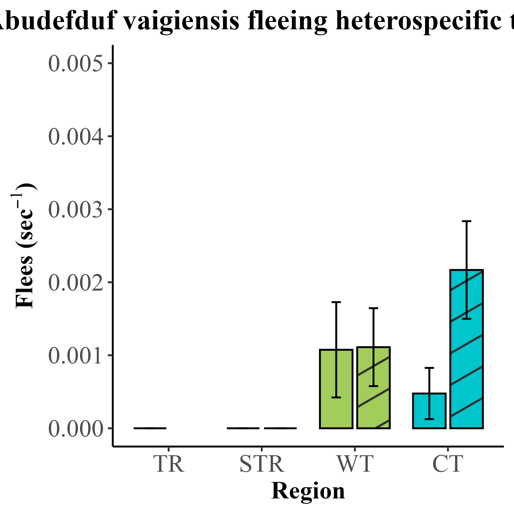

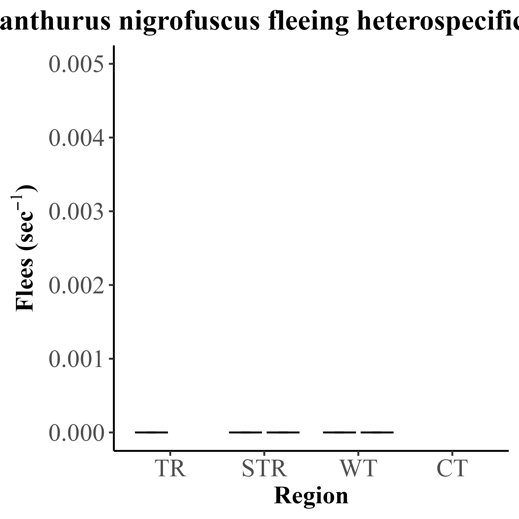

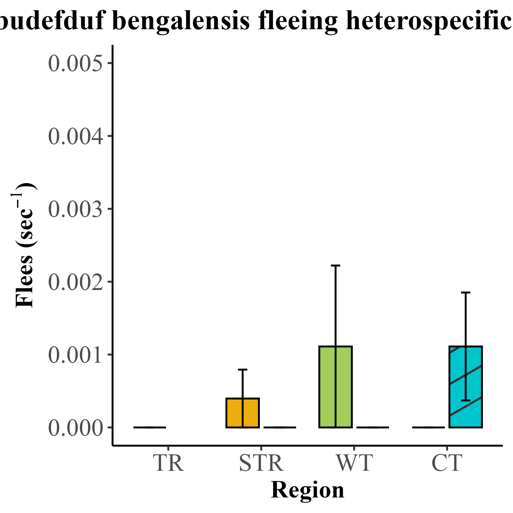

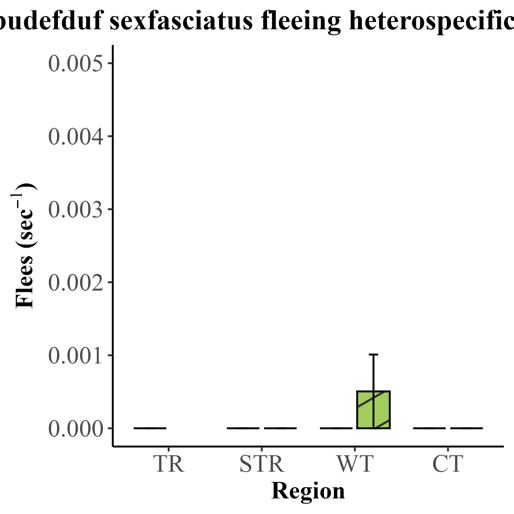


**Region**


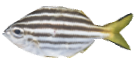

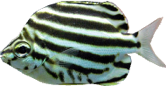

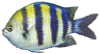

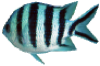

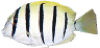

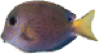


**(A)**

**(B)**

**(C)**

**(D)**

**(E)**

**(F)**


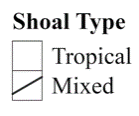


**Temperate Fish Species**

**Tropical Fish Species**


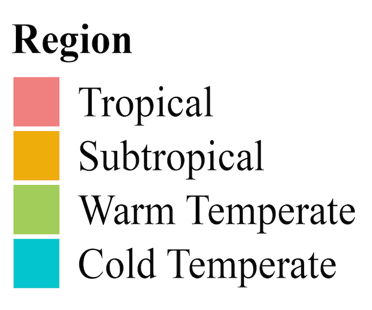


**Fleeing Heterospecific Temperate Fish**

**Region**

**0**

**0**

**0**

**0**

**0**

**0**

**0**

**0**

**0**

**0**

**0**

**0**

**0**

**0**

**0**

**0**

**0**

**0**

**0**

**0**

**0**

**0**

**0**

**0**

**0**

**0**

**0**

**N/A**

**N/A**

**N/A**

**N/A**

**N/A**

**N/A**

**N/A**

**Figure S8:** Fleeing from heterospecific temperate species fish behaviour (flees.sec^-1^) of the focal tropical fish species (A) *Abudefduf bengalensis,* (B) *Abudefduf sexfasciatus,* (C) *Abudefduf vaigiensis,* (D) *Acanthurus nigrofuscus* and (E) *Acanthurus triostegus*  in tropical-only shoals (solid bars) and in mixed-species shoals (hatched bars), and fleeing from heterospecific temperate species shoal mate behaviour (flees.sec^-1^) of the focal temperate fish species (F) *Microcanthus joyceae* and (G) *Atypichthys strigatus*  in temperate-only shoals (solid bars) and in mixed-species shoals (hatched) across regions (TR = Tropical; STR = Subtropical; WT = Warm Temperate, CT = Cold Temperate). Tables S12 and S23 correspond to statistical outputs visualised in Fig. S8. Error bars represent standard errors. NS = not significant (P > 0.05). Null = null model retained for analysis. N/A denotes that fish species was not sampled in specified shoal type within a region or that focal species was not sampled within specific region.

**N/A**


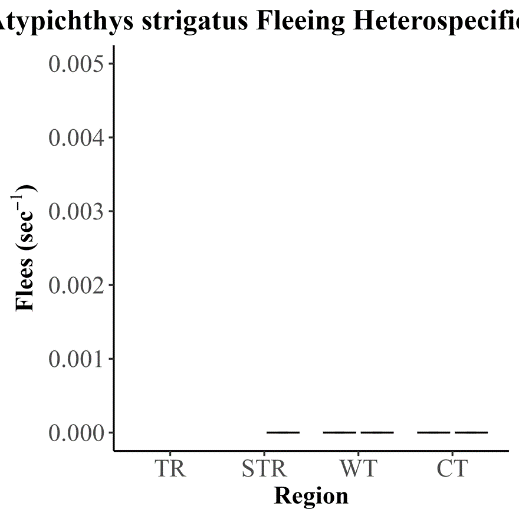


**(G)**

**0**

**0**

**0**

**N/A**

**N/A**


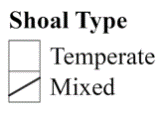


**0**

**0**


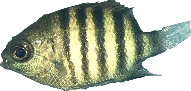


**Shoal Type: P = 0.017**

**Null**

**Chasing Conspecifics**


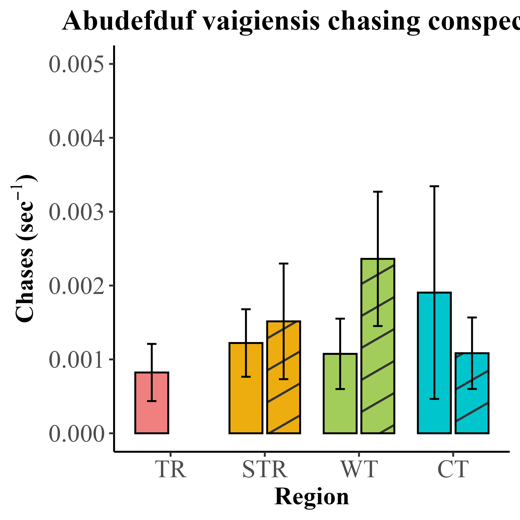

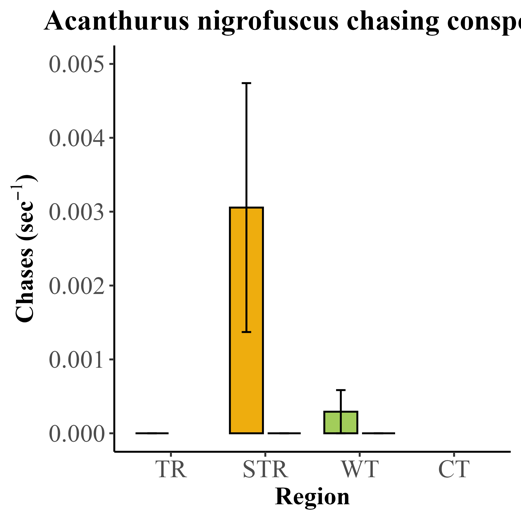

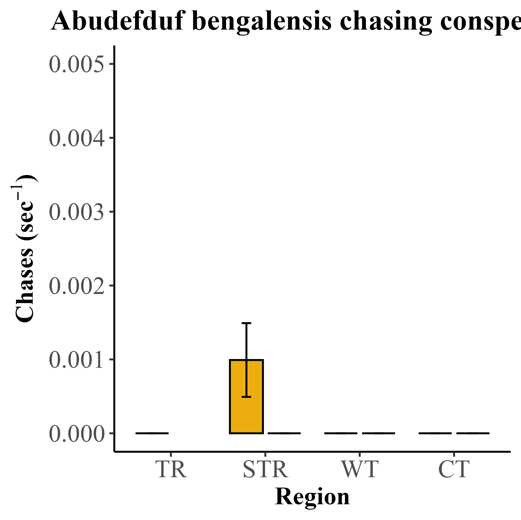

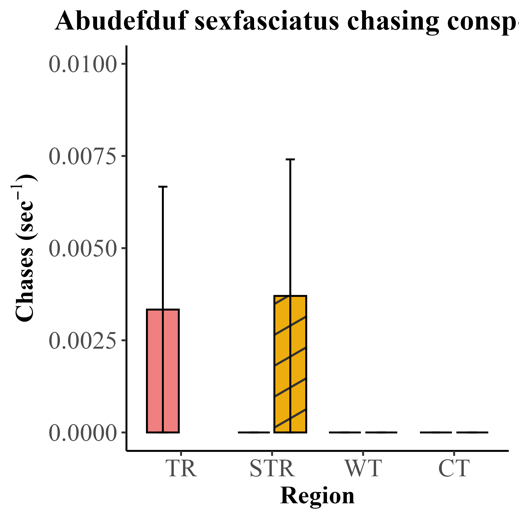

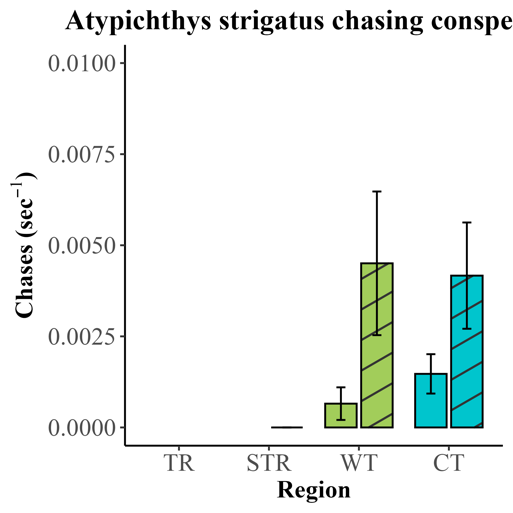

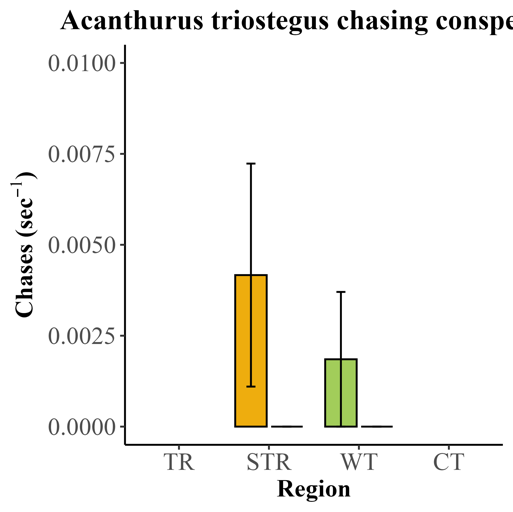

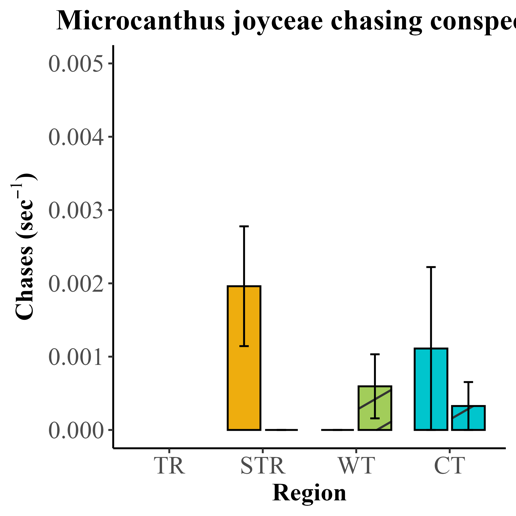

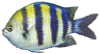

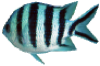

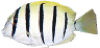


**(A)**

**(B)**

**(C)**

**(D)**

**(E)**

**(F)**

**(G)**

**Temperate Fish Species**

**Tropical Fish Species**

**Region**

**Region**

**N/A**

**N/A**

**N/A**

**N/A**

**N/A**

**N/A**

**N/A**

**N/A**

**N/A**

**N/A**

**0**

**0**

**0**

**0**

**0**

**0**

**0**

**0**

**0**

**0**

**0**

**0**

**0**

**0**

**0**

**0**

**0**

**0**

**0**

**Figure S9:** Antagonistic chasing conspecific shoal fish behaviour (chase.sec^-1^) of the focal tropical fish species (A) *Abudefduf bengalensis,* (B) *Abudefduf sexfasciatus,* (C) *Abudefduf vaigiensis,* (D) *Acanthurus nigrofuscus* and (E) *Acanthurus triostegus*  in tropical-only shoals (solid bars) and in mixed-species shoals (hatched bars), and antagonistic chasing conspecific shoal mate behaviour (chase.sec^-1^) of the focal temperate fish species (F) *Microcanthus joyceae* and (G) *Atypichthys strigatus*  in temperate-only shoals (solid bars) and in mixed-species shoals (hatched) across regions (TR = Tropical; STR = Subtropical; WT = Warm Temperate, CT = Cold Temperate). Error bars represent standard errors. NS = not significant (P > 0.05). N/A denotes that fish species was not sampled in specified shoal type within a region or that focal species was not sampled within specific region. Corresponding statistical outputs are located in Tables S10 and S16.

**NS**

**Species: P = 0.002
Shoal Type: P = 0.023**

**Region**

**(A)**

**(B)**

**(C)**

**(D)**

**(E)**

**(F)**

**(G)**

**Temperate Fish Species**

**Tropical Fish Species**

**Region**

**Fleeing Conspecifics**

**0**

**0**

**0**

**0**

**0**

**0**

**0**

**0**

**0**

**0**

**0**

**0**

**0**

**0**

**0**

**0**

**0**

**0**

**0**

**0**

**0**

**0**

**0**

**0**

**N/A**

**N/A**

**N/A**

**N/A**

**N/A**

**N/A**

**N/A**

**N/A**

**N/A**

**Figure S10:** Fleeing from conspecific fish behaviour (flees.sec^-1^) of the focal tropical fish species (A) *Abudefduf bengalensis,* (B) *Abudefduf sexfasciatus,* (C) *Abudefduf vaigiensis,* (D) *Acanthurus nigrofuscus* and (E) *Acanthurus triostegus*  in tropical-only shoals (solid bars) and in mixed-species shoals (hatched bars), and fleeing from conspecific shoal mate behaviour (flees.sec^-1^) of the focal temperate fish species (F) *Microcanthus joyceae* and (G) *Atypichthys strigatus*  in temperate-only shoals (solid bars) and in mixed-species shoals (hatched) across regions (TR = Tropical; STR = Subtropical; WT = Warm Temperate, CT = Cold Temperate). Tables S13 and S24 show corresponding statistical outputs for Fig. S10. Error bars represent standard errors. NS = not significant (P > 0.05). N/A denotes that fish species was not sampled in specified shoal type within a region or that focal species was not sampled within specific region.

**NS**

**NS**

**N/A**

**Table S3:** GLMM, Type III Wald Chi-Square tests and resulting Tukey post hoc tests of all tropical fish species bite rate. Final model selected are presented below using log (X+1) transformed bite rate data. GLMM estimates for fixed, continuous and random effects. Note: **bold** P-value denotes significant effect for predictor variable and corresponding Type III Wald Chi-Squared test. Model AIC = -553.3. Conditional *R_2_* = 0.121 and Marginal *R_2_* = 0.121. DF = Degrees of freedom.

| Log bite rate ~ Region + Shoal Type * Species + (1 \| Region:Location); family: tweedie; Zero inflation: ~Region + Shoal Type | | | | | | | | | | | | | | |
| --- | --- | --- | --- | --- | --- | --- | --- | --- | --- | --- | --- | --- | --- | --- |
| Variable | | **Estimate** | | | | **Standard Error** | | | | **Z value** | | | **P value** | |
| Intercept | | -1.640 | | | | 0.111 | | | | -14.746 | | | < 0.001 | |
| Region (Subtropical) | | 0.446 | | | | 0.075 | | | | 5.937 | | | <0.001 | |
| Region (Tropical) | | 0.468 | | | | 0.095 | | | | 4.917 | | | <0.001 | |
| Region (Warm temperate) | | 0.216 | | | | 0.072 | | | | 3.015 | | | 0.003 | |
| Shoal Type (Tropical) | | -0.316 | | | | 0.130 | | | | -2.423 | | | 0.015 | |
| Species (*A. sexfasciatus*) | | -0.157 | | | | 0.156 | | | | -1.010 | | | 0.312 | |
| Species (*A. vaigiensis*) | | 0.036 | | | | 0.117 | | | | 0.307 | | | 0.758 | |
| Species (*A. nigrofuscus*) | | -0.338 | | | | 0.190 | | | | -1.778 | | | 0.075 | |
| Species (*A. triostegus*) | | -0.090 | | | | 0.197 | | | | -0.457 | | | 0.648 | |
| Shoal Type (Tropical-only) * Species (*A. sexfasciatus*) | | -0.194 | | | | -0.194 | | | | 0.203 | | | 0.339 | |
| Shoal Type (Tropical-only) * Species (*A. vaigiensis*) | | -0.020 | | | | -0.204 | | | | 0.142 | | | 0.886 | |
| Shoal Type (Tropical-only) * Species (*A. nigrofuscus*) | | 0.603 | | | | 0.603 | | | | 0.218 | | | 0.006 | |
| Shoal Type (Tropical-only) * Species (*A. triostegus*) | | 0.594 | | | | 0.594 | | | | 0.224 | | | 0.008 | |
| Type III Wald Chi-Square Test | | **Response Variable:**  **Log Bite Rate for Tropical Fish Species** | | | | | | | | | | | | |
| Variable | | **Chi squares** | | | | **DF** | | | | **P value** | | |  | |
| Intercept | | 217.452 | | | | 1 | | | | **< 0.001** | | |  | |
| Region | | 40.069 | | | | 3 | | | | **<0.001** | | |  | |
| Shoal Type | | 5.869 | | | | 1 | | | | **0.015** | | |  | |
| Species | | 6.651 | | | | 4 | | | | 0.156 | | |  | |
| Shoal Type * Species | | 21.185 | | | | 4 | | | | **<0.001** | | |  | |
| Tukey Post hoc test for Fixed Factor ‘Region’ | | | | | | | | | | | | | | |
| Contrast | | | **Estimate** | | **Standard Error** | | | **DF** | | | **Z Ratio** | | | **P value** |
| Cold temperate – Subtropical | | | -0.446 | | 0.075 | | | Inf | | | -5.937 | | | **<0.001** |
| Cold temperate – Tropical | | | -0.468 | | 0.095 | | | Inf | | | -4.917 | | | **<0.001** |
| Cold temperate ­– Warm temperate | | | -0.216 | | 0.072 | | | Inf | | | -3.015 | | | **0.014** |
| Subtropical – Tropical | | | -0.022 | | 0.080 | | | Inf | | | -0.279 | | | 0.923 |
| Subtropical – Warm temperate | | | 0.230 | | 0.063 | | | Inf | | | 3.653 | | | **0.002** |
| Tropical – Warm temperate | | | 0.253 | | 0.089 | | | Inf | | | 2.836 | | | **0.024** |
| Tukey Post hoc test for contrasts of levels of fixed factor ‘Species’ within Fixed Factor ‘Shoal Type’ | | | | | | | | | | | | | | |
| Contrast | **Estimate** | | | **Standard Error** | | | **DF** | | **Z Ratio** | | | **P value** | | |
| *A. bengalensis:* Mixed – Tropical-only | 0.316 | | | 0.130 | | | Inf | | 2.423 | | | **0.015** | | |
| *A. sexfasciatus:* Mixed – Tropical-only | 0.093 | | | 0.165 | | | Inf | | 3.083 | | | **0.002** | | |
| *A. vaigiensis* Mixed – Tropical-only | 0.076 | | | 0.074 | | | Inf | | 4.519 | | | **<0.001** | | |
| *A. nigrofuscus:* Mixed – Tropical-only | -0.064 | | | 0.176 | | | Inf | | -1.636 | | | 0.102 | | |
| *A. triostegus:* Mixed – Tropical-only | -0.091 | | | 0.183 | | | 455 | | -1.519 | | | 0.129 | | |
| Tukey Post hoc test for contrasts of level ‘Mixed’ of fixed factor ‘Shoal Type’ within Fixed Factor ‘Species’ | | | | | | | | | | | | | | |
| Contrast | **Estimate** | | | **Standard Error** | | | **DF** | | **Z Ratio** | | | **P value** | | |
| *A. bengalensis* – *A. sexfasciatus* | 0.157 | | | 0.156 | | | Inf | | 1.010 | | | 0.851 | | |
| *A. bengalensis* – *A. vaigiensis* | -0.036 | | | 0.117 | | | Inf | | 0.307 | | | 0.998 | | |
| *A. bengalensis* – *A. nigrofuscus* | 0.338 | | | 0.190 | | | Inf | | 1.778 | | | 0.386 | | |
| *A. bengalensis* – *A. triostegus* | 0.090 | | | 0.197 | | | Inf | | 0.457 | | | 0.991 | | |
| *A. sexfasciatus* – *A. vaigiensis* | -0.193 | | | 0.127 | | | Inf | | -1.526 | | | 0.546 | | |
| *A. sexfasciatus* – *A. nigrofuscus* | 0.181 | | | 0.196 | | | Inf | | 0.924 | | | 0.888 | | |
| *A. sexfasciatus* – *A. triostegus* | -0.67 | | | 0.203 | | | Inf | | -0.331 | | | 0.997 | | |
| *A. vaigiensis* – *A. nigrofuscus* | 0.374 | | | 0.168 | | | Inf | | 2.230 | | | 0.169 | | |
| *A. vaigiensis* – *A. triostegus* | 0.126 | | | 0.175 | | | Inf | | 0.719 | | | 0.952 | | |
| *A. nigrofuscus* – *A. triostegus* | -0.248 | | | 0.227 | | | Inf | | -1.092 | | | 0.811 | | |
| Tukey Post hoc test for contrasts of level ‘Tropical-only’ of fixed factor ‘Shoal Type’ within Fixed Factor ‘Species’ | | | | | | | | | | | | | | |
| Contrast | **Estimate** | | | **Standard Error** | | | **DF** | | **Z Ratio** | | | **P value** | | |
| *A. bengalensis* – *A. sexfasciatus* | 0.351 | | | 0.131 | | | Inf | | 2.689 | | | 0.056 | | |
| *A. bengalensis* – *A. vaigiensis* | -0.016 | | | 0.080 | | | Inf | | -0.194 | | | 0.999 | | |
| *A. bengalensis* – *A. nigrofuscus* | -0.265 | | | 0.108 | | | Inf | | -2.459 | | | 1.000 | | |
| *A. bengalensis* – *A. triostegus* | -0.504 | | | 0.109 | | | Inf | | -4.639 | | | **<0.001** | | |
| *A. sexfasciatus* – *A. vaigiensis* | -0.367 | | | 0.121 | | | Inf | | -3.025 | | | **0.021** | | |
| *A. sexfasciatus* – *A. nigrofuscus* | -0.617 | | | 0.140 | | | Inf | | -4.393 | | | **<0.001** | | |
| *A. sexfasciatus* – *A. triostegus* | -0.855 | | | 0.141 | | | Inf | | -6.079 | | | **<0.001** | | |
| *A. vaigiensis* – *A. nigrofuscus* | -0.250 | | | 0.094 | | | Inf | | -2.667 | | | 0.059 | | |
| *A. vaigiensis* – *A. triostegus* | -0.488 | | | 0.094 | | | Inf | | -5.197 | | | **<0.001** | | |
| *A. nigrofuscus* – *A. triostegus* | -0.239 | | | 0.113 | | | Inf | | -2.106 | | | 0.217 | | |

**Table S4:** GLMM, Type III Wald Chi-Square tests and resulting Tukey post hocs of all tropical fish species’ sheltering behaviour. Final model selected are presented below using log (X+1) transformed sheltering behaviour data. GLMM estimates for fixed, continuous and random effects. Note: **bold** P-value denotes significant effect for predictor variable and corresponding Type III Wald Chi-Squared test. Model AIC = 822.4. Marginal *R_2_* = 0.266 and Conditional *R_2_* = 0.488. DF = Degrees of freedom.

| Final model: Log sheltering ~ Region + Shoal Type + Species + (1 \| Region:Location); family: tweedie | | | | | | | | | |
| --- | --- | --- | --- | --- | --- | --- | --- | --- | --- |
| Variable | **Estimate** | | | **Standard Error** | | **Z value** | | **P value** | |
| Intercept | 1.287 | | | 0.114 | | 11.266 | | **<0.001** | |
| Region (Subtropical) | -0.197 | | | 0.151 | | -1.306 | | 0.192 | |
| Region (Tropical) | -0.599 | | | 0.173 | | -3.444 | | **<0.001** | |
| Region (Warm temperate) | -0.111 | | | 0.126 | | -0.883 | | 0.377 | |
| Shoal Type (Tropical) | 0.160 | | | 0.055 | | 2.899 | | **0.004** | |
| Species (*A. sexfasciatus*) | -0.018 | | | 0.094 | | -0.195 | | 0.845 | |
| Species (*A. vaigiensis*) | -0.138 | | | 0.060 | | -2.310 | | **0.021** | |
| Species (*A. nigrofuscus*) | -0.118 | | | 0.092 | | -1.283 | | 0.200 | |
| Species (*A. triostegus*) | -0.234 | | | 0.094 | | -2.501 | | **0.012** | |
| Type III Wald Chi-Square Test | **Response Variable:**  **Log Sheltering for Tropical Fish Species** | | | | | | | | |
| Variable | **Chi squares** | | | **DF** | | **P value** | |  | |
| Intercept | 126.924 | | | 1 | | **<0.001** | |  | |
| Region | 12.385 | | | 3 | | **0.006** | |  | |
| Shoal Type | 8.404 | | | 1 | | **0.004** | |  | |
| Species | 10.286 | | | 4 | | **0.036** | |  | |
| Tukey Post hoc test for fixed factor ‘Region’ | | | | | | | | | |
| Contrast | | **Estimate** | **Standard Error** | | **DF** | | **Z Ratio** | | **P value** |
| Cold temperate – Subtropical | | 0.197 | 0.151 | | Inf | | 1.306 | | 0.559 |
| Cold temperate – Tropical | | 0.599 | 0.174 | | Inf | | 3.444 | | **0.003** |
| Cold temperate ­– Warm temperate | | 0.111 | 0.111 | | Inf | | 0.883 | | 0.814 |
| Subtropical – Tropical | | 0.402 | 0.183 | | Inf | | 2.198 | | 0.124 |
| Subtropical – Warm temperate | | -0.086 | 0.136 | | Inf | | -0.629 | | 0.922 |
| Tropical – Warm temperate | | -0.488 | 0.166 | | Inf | | -2.942 | | **0.017** |
| Tukey Post hoc test for contrasts of levels of fixed factor ‘Species’ | | | | | | | | | |
| Contrast | | **Estimate** | **Standard Error** | | **DF** | | **Z Ratio** | | **P value** |
| *A. bengalensis* – *A. sexfasciatus* | | 0.018 | 0.094 | | Inf | | 0.195 | | 1.000 |
| *A. bengalensis* – *A. vaigiensis* | | 0.138 | 0.060 | | Inf | | 2.310 | | 0.142 |
| *A. bengalensis* – *A. nigrofuscus* | | 0.118 | 0.092 | | Inf | | 1.283 | | 0.702 |
| *A. bengalensis* – *A. triostegus* | | 0.234 | 0.936 | | Inf | | 2.501 | | 0.090 |
| *A. sexfasciatus* – *A. vaigiensis* | | 0.120 | 0.085 | | Inf | | 1.421 | | 0.614 |
| *A. sexfasciatus* – *A. nigrofuscus* | | 0.010 | 0.105 | | Inf | | 0.951 | | 0.877 |
| *A. sexfasciatus* – *A. triostegus* | | 0.216 | 0.105 | | Inf | | 2.048 | | 0.243 |
| *A. vaigiensis* – *A. nigrofuscus* | | -0.020 | 0.084 | | Inf | | -0.243 | | 0.999 |
| *A. vaigiensis* – *A. triostegus* | | -0.096 | 0.086 | | Inf | | 1.109 | | 0.802 |
| *A. nigrofuscus* – *A. triostegus* | | 0.115 | 0.080 | | Inf | | 1.443 | | 0.600 |

**Table S5:** GLMM, Type III Wald Chi-Square tests and resulting Tukey post hoc tests of all tropical fish species’ chasing behaviour towards heterospecific temperate fish responses. Final model selected model presented below uses log (X+1) transformed tropical fish species’ chasing behaviour towards heterospecific temperate fish data. GLMM estimates for fixed, continuous and random effects. Note: **bold** P-value denotes significant effect for predictor variable and corresponding Type III Wald Chi-Squared test. Model AIC = -411.6. Marginal *R_2_* = 0.963 and Conditional *R_2_* = 0.970. DF = Degrees of freedom.

| Final model: Log Chasing Heterospecific Temperate Fish ~ Region + Shoal Type + (1 \| Region:Location); family: tweedie | | | | | | | | | |
| --- | --- | --- | --- | --- | --- | --- | --- | --- | --- |
| Variable | **Estimate** | | | **Standard Error** | | **Z value** | | **P value** | |
| Intercept | -6.248 | | | 0.588 | | -10.625 | | **<0.001** | |
| Region (Subtropical) | 1.229 | | | 0.647 | | 1.900 | | 0.057 | |
| Region (Tropical) | -18.594 | | | 5938.795 | | -0.003 | | 0.998 | |
| Region (Warm temperate) | -1.342 | | | 0.743 | | -1.806 | | 0.071 | |
| Shoal Type (Tropical) | -1.182 | | | 0.339 | | -3.486 | | **<0.001** | |
| Type III Wald Chi-Square Test | **Response Variable:**  **Log Chasing Heterospecific Tropical Fish for Tropical Fish Species** | | | | | | | | |
| Variable | **Chi squares** | | | **DF** | | **P value** | |  | |
| Intercept | 112.888 | | | 1 | | **<0.001** | |  | |
| Region | 13.523 | | | 3 | | **0.004** | |  | |
| Shoal Type | 12.149 | | | 1 | | **<0.001** | |  | |
| Tukey Post hoc test for fixed factor ‘Region’ | | | | | | | | | |
| Contrast | | **Estimate** | **Standard Error** | | **DF** | | **T Ratio** | | **P value** |
| Cold temperate – Subtropical | | -1.23 | 0.647 | | Inf | | -1.900 | | 0.228 |
| Cold temperate – Tropical | | 18.59 | 5938.795 | | Inf | | 0.003 | | 1.000 |
| Cold temperate ­– Warm temperate | | 1.34 | 0.743 | | Inf | | 1.806 | | 0.271 |
| Subtropical – Tropical | | 19.82 | 5938.795 | | Inf | | 0.003 | | 1.000 |
| Subtropical – Warm temperate | | 2.57 | 0.705 | | Inf | | 3.645 | | **0.015** |
| Tropical – Warm temperate | | -17.25 | 5938.795 | | Inf | | -0.003 | | 1.000 |

**Table S6:** GLMM, Type III Wald Chi-Square tests and resulting Tukey post hoc tests of the focal tropical fish’s (*Abudefduf vaigiensis*) relative lateralization (L_R_) responses. Final model selected are presented below using raw relative lateralization data. GLMM estimates for fixed, continuous and random effects. Note: **bold** P-value denotes significant effect for predictor variable and corresponding Type III Wald Chi-Squared test. Model AIC = 105. Marginal *R_2_* = 0.121 and Conditional *R_2_* = 0.121. DF = Degrees of freedom.

| Final model: L_R_ ~ Region + (1 \| Region:Location); family: gaussian | | | | | | | | | |
| --- | --- | --- | --- | --- | --- | --- | --- | --- | --- |
| Variable | **Estimate** | | | **Standard Error** | | **Z value** | | **P value** | |
| Intercept | 27.206 | | | 3.918 | | 6.945 | | **< 0.001** | |
| Region (Subtropical) | -28.488 | | | 6.489 | | -4.390 | | **<0.001** | |
| Region (Tropical) | -33.873 | | | 14.969 | | -2.263 | | **0.024** | |
| Region (Warm temperate) | -6.527 | | | 5.888 | | -1.108 | | 0.268 | |
| Type III Wald Chi-Square Test | **Response Variable:**  **Log L_R_ for Tropical Fish Species** | | | | | | | | |
| Variable | **Chi squares** | | | **DF** | | **P value** | |  | |
| Intercept | 48.229 | | | 1 | | **< 0.001** | |  | |
| Region | 22.640 | | | 3 | | **<0.001** | |  | |
| Tukey Post hoc test for fixed factor ‘Region’ | | | | | | | | | |
| Contrast | | **Estimate** | **Standard Error** | | **DF** | | **T Ratio** | | **P value** |
| Cold temperate – Subtropical | | 28.49 | 6.490 | | 160 | | 4.390 | | **<0.001** |
| Cold temperate – Tropical | | 33.87 | 15.000 | | 160 | | 2.263 | | 0.111 |
| Cold temperate ­– Warm temperate | | 6.530 | 5.890 | | 160 | | 1.108 | | 0.685 |
| Subtropical – Tropical | | 5.380 | 15.300 | | 160 | | 0.351 | | 0.985 |
| Subtropical – Warm temperate | | -21.96 | 6.79 | | 160 | | -3.235 | | **0.008** |
| Tropical – Warm temperate | | -27.35 | 15.10 | | 160 | | -1.811 | | 0.272 |

**Table S7:** GLMM, Type III Wald Chi-Square tests and resulting Tukey post hoc tests of the focal tropical fish’s (*Abudefduf vaigiensis*) absolute lateralization (L_A_) responses. Final model selected are presented below using log (X+1) transformed absolute lateralization data. GLMM estimates for fixed, continuous and random effects. Note: **bold** P-value denotes significant effect for predictor variable and corresponding Type III Wald Chi-Squared test. Model AIC = 357.0. Marginal *R_2_* = 0.100 and Conditional *R_2_* = 0.100. DF = Degrees of freedom.

| Final model: Log L_A_ ~ Region + (1 \| Region:Location); family: gaussian | | | | | | | | | |
| --- | --- | --- | --- | --- | --- | --- | --- | --- | --- |
| Variable | **Estimate** | | | **Standard Error** | | **Z value** | | **P value** | |
| Intercept | 3.532 | | | 0.066 | | 53.450 | | **< 0.001** | |
| Region (Subtropical) | -0.281 | | | 0.111 | | -2.540 | | **0.011** | |
| Region (Tropical) | -0.660 | | | 0.260 | | -2.540 | | **0.011** | |
| Region (Warm temperate) | 0.052 | | | 0.098 | | 0.53 | | 0.595 | |
| Type III Wald Chi-Square Test | **Response Variable:**  **L_A_ for Tropical Fish Species** | | | | | | | | |
| Variable | **Chi squares** | | | **DF** | | **P value** | |  | |
| Intercept | 2856.58 | | | 1 | | **< 0.001** | |  | |
| Region | 15.12 | | | 3 | | **0.002** | |  | |
| Tukey Post hoc test for fixed factor ‘Region’ | | | | | | | | | |
| Contrast | | **Estimate** | **Standard Error** | | **DF** | | **T Ratio** | | **P value** |
| Cold temperate – Subtropical | | 0.281 | 0.111 | | 159 | | 2.539 | | 0.058 |
| Cold temperate – Tropical | | 19.760 | 0.260 | | 159 | | 2.537 | | 0.058 |
| Cold temperate ­– Warm temperate | | -3.020 | -0.052 | | 159 | | -0.532 | | 0.951 |
| Subtropical – Tropical | | 9.320 | 0.267 | | 159 | | 1.419 | | 0.490 |
| Subtropical – Warm temperate | | -13.460 | 0.114 | | 159 | | -2.914 | | **0.021** |
| Tropical – Warm temperate | | -22.780 | 0.262 | | 159 | | -2.721 | | **0.036** |

**Table S8:** GLMM, Type III Wald Chi-Square tests and resulting Tukey post hocs of all tropical fish species’ flight initiation distance responses. Final model selected presented below using log (X+1) transformed tropical fish species’ flight initiation distance data. GLMM estimates for fixed, continuous and random effects. Note: **bold** P-value denotes significant effect for predictor variable and corresponding Type III Wald Chi-Squared test. Model AIC = 313.2. Marginal *R_2_* = 0.102 and Conditional *R_2_* = 0.102. DF = Degrees of freedom.

| Final model: Log FID ~ Region + Species + (1 \| Region:Location); family: gamma | | | | |
| --- | --- | --- | --- | --- |
| Variable | **Estimate** | **Standard Error** | **Z value** | **P value** |
| Intercept | 0.896 | 0.043 | 20.757 | **< 0.001** |
| Region (Subtropical) | -0.153 | 0.059 | -2.595 | **0.009** |
| Region (Tropical) | -0.068 | 0.098 | -0.698 | 0.485 |
| Region (Warm temperate) | -0.109 | 0.061 | -1.800 | 0.072 |
| Species (*A. nigrofuscus*) | -0.078 | 0.069 | -1.136 | 0.256 |
| Species (*A. triostegus*) | -0.140 | 0.064 | -2.177 | **0.030** |
| Type III Wald Chi-Square Test | **Response Variable:**  **Log FID for Tropical Fish Species** | | | |
| Variable | **Chi squares** | **DF** | **P value** |  |
| Intercept | 430.872 | 1 | **< 0.001** |  |
| Region | 6.805 | 3 | 0.078 |  |
| Species | 5.145 | 2 | 0.076 |  |

**Table S9:** GLMM, Type III Wald Chi-Square tests and resulting Tukey post hoc tests of all tropical fish species’ chasing behaviour towards heterospecific tropical fish responses. Final model selected are presented below using log (X+1) transformed tropical fish species’ chasing behaviour towards heterospecific tropical fish data. GLMM estimates for fixed, continuous and random effects. Note: **bold** P-value denotes significant effect for predictor variable and corresponding Type III Wald Chi-Squared test. Model AIC = -102.6. Marginal *R_2_* = 0.979 and Conditional *R_2_* = 0.983. DF = Degrees of freedom.

| Final model: Log_Chasing_Heterospecific_Tropical_Fish ~ Region + Shoal_Type + Species + (1 \| Region:Location); family: tweedie | | | | |
| --- | --- | --- | --- | --- |
| Variable | **Estimate** | **Standard Error** | **Z value** | **P value** |
| Intercept | -31.383 | 15002.697 | -0.002 | 0.998 |
| Region (Subtropical) | 24.728 | 15002.697 | 0.002 | 0.999 |
| Region (Tropical) | 24.576 | 15002.697 | 0.002 | 0.999 |
| Region (Warm temperate) | 23.007 | 15002.697 | 0.002 | 0.999 |
| Shoal Type (Tropical) | 0.651 | 0.498 | 1.306 | 0.191 |
| Species (*A. sexfasciatus*) | -1.242 | 0.723 | -1.718 | 0.086 |
| Species (*A. vaigiensis*) | -0.256 | 0.326 | -0.783 | 0.433 |
| Species (*A. nigrofuscus*) | -0.502 | 0.503 | -0.998 | 0.318 |
| Species (*A. triostegus*) | -1.766 | 0.867 | -2.037 | **0.042** |
| Type III Wald Chi-Square Test | **Response Variable:**  **Log Chasing Heterospecific Tropical Fish for Tropical Fish Species** | | | |
| Variable | **Chi squares** | **DF** | **P value** |  |
| Intercept | 0.000 | 1 | 0.998 |  |
| Region | 4.603 | 3 | 0.203 |  |
| Shoal Type | 1.707 | 1 | 0.191 |  |
| Species | 6.549 | 4 | 0.162 |  |

**Table S10:** GLMM and Type III Wald Chi-Square tests of all tropical fish species’ chasing behaviour towards conspecific fish responses. Final model selected model presented below uses log (X+1) transformed tropical fish species’ chasing behaviour towards conspecific fish data. GLMM estimates for fixed, continuous and random effects. Note: **bold** P-value denotes significant effect for predictor variable and corresponding Type III Wald Chi-Squared test. Model AIC = -47.5. Marginal *R_2_* = 0.178 and Conditional *R_2_* = 0.241. DF = Degrees of freedom.

| Final model: Log Chasing Conspecific Tropical Fish ~ Region + Shoal Type + Species + (1 \| Region:Location); family: tweedie | | | | |
| --- | --- | --- | --- | --- |
| Variable | **Estimate** | **Standard Error** | **Z value** | **P value** |
| Intercept | -8.606 | 0.840 | -10.245 | 0.998 |
| Region (Subtropical) | 0.519 | 0.628 | 0.826 | 0.409 |
| Region (Tropical) | -0.291 | 0.815 | -0.357 | 0.721 |
| Region (Warm temperate) | 0.010 | 0.600 | 0.017 | 0.986 |
| Shoal Type (Tropical) | 0.227 | 0.386 | 0.586 | 0.558 |
| Species (*A. sexfasciatus*) | 0.833 | 1.869 | 0.959 | 0.338 |
| Species (*A. vaigiensis*) | 1.656 | 0.638 | 2.596 | **0.009** |
| Species (*A. nigrofuscus*) | 1.312 | 0.763 | 1.720 | 0.086 |
| Species (*A. triostegus*) | 2.055 | 0.738 | 2.785 | **0.005** |
| Type III Wald Chi-Square Test | **Response Variable:**  **Log Chasing Conspecific Tropical Fish for Tropical Fish Species** | | | |
| Variable | **Chi squares** | **DF** | **P value** |  |
| Intercept | 104.967 | 1 | **<0.001** |  |
| Region | 1.597 | 3 | 0.661 |  |
| Shoal Type | 0.344 | 1 | 0.558 |  |
| Species | 10.024 | 4 | **0.040** |  |

**Table S11:** GLMM, Type III Wald Chi-Square tests and resulting Tukey post hocs of all tropical fish species’ fleeing behaviour from heterospecific tropical fish. Final model selected model presented below uses log (X+1) transformed tropical fish species’ fleeing behaviour from heterospecific tropical fish data. GLMM estimates for fixed, continuous and random effects. Note: **bold** P-value denotes significant effect for predictor variable and corresponding Type III Wald Chi-Squared test. Model AIC = -17.2. Marginal *R_2_* < 0.001 and Conditional *R_2_* < 0.001. DF = Degrees of freedom. The null model was retained as the best fit model and reported below.

| Final model: Log fleeing from heterospecific tropical fish ~ 1 + (1 \| Region:Location); family: tweedie | | | | |
| --- | --- | --- | --- | --- |
| Variable | **Estimate** | **Standard Error** | **Z value** | **P value** |
| Intercept | -8.056 | 0.227 | -35.48 | **<0.001** |

**Table S12:** GLMM and Type III Wald Chi-Square tests of all tropical fish species’ fleeing behaviour from heterospecific temperate fish. Final model selected model presented below uses log (X+1) transformed tropical fish species’ fleeing behaviour from heterospecific temperate fish data. GLMM estimates for fixed, continuous and random effects. Note: **bold** P-value denotes significant effect for predictor variable and corresponding Type III Wald Chi-Squared test. Model AIC = -27.0. Marginal *R_2_* = 0.079 and Conditional *R_2_* = 0.244. DF = Degrees of freedom.

| Final model: Log fleeing from heterospecific temperate fish ~ Shoal_Type + (1 \| Region:Location) | | | | |
| --- | --- | --- | --- | --- |
| Variable | **Estimate** | **Standard Error** | **Z value** | **P value** |
| Intercept | -7.453 | 0.522 | -14.270 | **<0.001** |
| Region (Subtropical) | -1.162 | 0.486 | -2.392 | **0.017** |
| Type III Wald Chi-Square Test | **Response Variable:**  **Log fleeing heterospecific temperate fish for Tropical Fish Species** | | | |
| Variable | **Chi squares** | **DF** | **P value** |  |
| Intercept | 203.640 | 1 | **<0.001** |  |
| Shoal Type | 5.721 | 1 | **0.017** |  |

**Table S13:** GLMM, Type III Wald Chi-Square tests and resulting Tukey post hocs of all tropical fish species’ fleeing behaviour from conspecific tropical fish. Final model selected model presented below uses log (X+1) transformed tropical fish species’ fleeing behaviour from conspecific tropical fish data. GLMM estimates for fixed, continuous and random effects. Note: **bold** P-value denotes significant effect for predictor variable and corresponding Type III Wald Chi-Squared test. Model AIC = 67.2. Marginal *R_2_* = 0.257 and Conditional *R_2_* = 0.594. DF = Degrees of freedom.

| Final model: Log fleeing from conspecific tropical fish ~ Region + Species + (1 \| Region:Location); family: tweedie | | | | |
| --- | --- | --- | --- | --- |
| Variable | **Estimate** | **Standard Error** | **Z value** | **P value** |
| Intercept | -8.288 | 1.734 | -4.780 | <0.001 |
| Region (Subtropical) | 2.949 | 1.804 | 1.634 | 0.102 |
| Region (Tropical) | 0.732 | 2.131 | 0.343 | 0.731 |
| Region (Warm temperate) | 1.435 | 1.764 | 0.814 | 0.416 |
| Type III Wald Chi-Square Test | **Response Variable:**  **Log fleeing conspecific tropical fish for Tropical Fish Species** | | | |
| Variable | **Chi squares** | **DF** | **P value** |  |
| Intercept | 22.847 | 1 | <0.001 |  |
| Region | 3.361 | 3 | 0.339 |  |

**Table S14:** GLMM, Type III Wald Chi-Square tests and resulting Tukey post hocs of all temperate fish species fleeing behaviour from heterospecific tropical fish. Final model selected model presented below uses log (X+1) transformed temperate fish species fleeing behaviour from heterospecific tropical fish data. GLMM estimates for fixed, continuous and random effects. Note: **bold** P-value denotes significant effect for predictor variable and corresponding Type III Wald Chi-Squared test. Model AIC = -336.8. Marginal *R_2_* = 0.526 and Conditional *R_2_* = 0.556. DF = Degrees of freedom.

| Final Model: Log_fleeing heterospecific tropical fish~ Region + Species + (1 \| Region:Location); family: tweedie | | | | | | | | | |
| --- | --- | --- | --- | --- | --- | --- | --- | --- | --- |
| Variable | **Estimate** | | | **Standard Error** | | **Z value** | | **P value** | |
| Intercept | -5.909 | | | 0.457 | | -12.944 | | **<0.001** | |
| Region (Subtropical) | 1.715 | | | 0.464 | | 3.695 | | **<0.001** | |
| Region (Warm temperate) | -1.229 | | | 0.567 | | -2.167 | | **0.030** | |
| Species | -1.364 | | | 0.421 | | -3.242 | | **0.001** | |
| Type III Wald Chi-Square Test | **Response Variable:**  **Log fleeing from heterospecific tropical fish for Temperate Fish Species** | | | | | | | | |
| Variable | **Chi squares** | | | **DF** | | **P value** | |  | |
| Intercept | 167.544 | | | 1 | | **<0.001** | |  | |
| Region | 27.729 | | | 2 | | **<0.001** | |  | |
| Species | 10.510 | | | 1 | | **0.001** | |  | |
| Tukey post hoc test for fixed factor ‘Region’ | | | | | | | | | |
| Contrast | | **Estimate** | **Standard Error** | | **DF** | | **T Ratio** | | **P value** |
| Cold temperate – Subtropical | | -1.710 | 0.464 | | Inf | | -3.695 | | **<0.001** |
| Cold temperate – Warm temperate | | 1.230 | 0.567 | | Inf | | 2.167 | | 0.077 |
| Subtropical ­– Warm temperate | | 2.940 | 0.589 | | Inf | | 4.996 | | **<0.001** |

**Table S15:** GLMM, Type III Wald Chi-Square tests and resulting Tukey post hocs of all temperate fish species bite rate. Final model selected are presented below using log (X+1) transformed bite rate data. GLMM estimates for fixed, continuous and random effects. Note: **bold** P-value denotes significant effect for predictor variable and corresponding Type III Wald Chi-Squared test. Model AIC = -335.9. Marginal *R_2_* = 0.276 and Conditional *R_2_* = 0.283. DF = Degrees of freedom.

| Final model: Log bite rate ~ Region + Shoal Type * Species + (1 \| Region:Location), family: gaussian | | | | | | | | | |
| --- | --- | --- | --- | --- | --- | --- | --- | --- | --- |
| Variable | | **Estimate** | | **Standard Error** | | | **Z value** | | **P value** |
| Intercept | | 0.216 | | 0.017 | | | 12.991 | | **< 0.001** |
| Region (Subtropical) | | -0.028 | | 0.027 | | | -1.030 | | 0.303 |
| Region (Warm temperate) | | -0.005 | | 0.018 | | | -0.260 | | 0.795 |
| Shoal Type (Temperate) | | 0.062 | | 0.020 | | | 3.152 | | **0.002** |
| Species | | -0.098 | | 0.020 | | | -4.916 | | **<0.001** |
| Shoal Type (Temperate) * Species (*M. joyceae*) | | -0.067 | | 0.0325 | | | -2.047 | | **0.041** |
| Type III Wald Chi-Square Test | | **Response Variable:**  **Log Bite Rate for Temperate Fish Species** | | | | | | | |
| Variable | | **Chi squares** | | **DF** | | | **P value** | |  |
| Intercept | | 168.765 | | 1 | | | **< 0.001** | |  |
| Region | | 1.084 | | 2 | | | 0.582 | |  |
| Shoal Type | | 9.933 | | 1 | | | **0.001** | |  |
| Species | | 24.165 | | 1 | | | **<0.001** | |  |
| Shoal Type * Species | | 4.191 | | 1 | | | **0.041** | |  |
| Tukey Post hoc test for contrasts of levels of fixed factor ‘Species’ within fixed factor ‘Shoal Type’ | | | | | | | | | |
| Contrast | **Estimate** | | **Standard Error** | | **DF** | **T Ratio** | | **P value** | |
| *A. strigatus:* Mixed – Temperate-only | -0.062 | | 0.020 | | 216 | -3.152 | | **0.002** | |
| *M. joyceae:* Mixed – Temperate-only | 0.004 | | 0.025 | | 216 | 0.166 | | 0.869 | |
| Tukey Post hoc test for contrasts of level ‘Mixed’ of fixed factor ‘Shoal Type’ within fixed factor ‘Species’ | | | | | | | | | |
| Contrast | **Estimate** | | **Standard Error** | | **DF** | **T Ratio** | | **P value** | |
| *A. strigatus – M. joyceae* | 0.098 | | 0.020 | | 216 | 4.916 | | **<0.001** | |
| Tukey Post hoc test for contrasts of level ‘Temperate-only’ of fixed factor ‘Shoal Type’ within fixed factor ‘Species’ | | | | | | | | | |
| Contrast | **Estimate** | | **Standard Error** | | **DF** | **T Ratio** | | **P value** | |
| *A. strigatus – M. joyceae* | 0.045 | | 0.027 | | 216 | 6.182 | | **<0.001** | |

**Table S16:** GLMM and Type III Wald Chi-Square tests of all temperate fish species’ chasing behaviour towards conspecific fish responses. Final model selected model presented below uses log (X+1) transformed temperate fish species’ chasing behaviour towards conspecific fish data. GLMM estimates for fixed, continuous and random effects. Note: **bold** P-value denotes significant effect for predictor variable and corresponding Type III Wald Chi-Squared test. Model AIC = -261.8. Marginal *R_2_* = 0.520 and Conditional *R_2_* = 0.643. DF = Degrees of freedom.

| Final model: Log Chasing Conspecific Temperate Fish ~ Region + Shoal Type + Species + (1 \| Region:Location); family: tweedie | | | | | | | |
| --- | --- | --- | --- | --- | --- | --- | --- |
| Variable | | **Estimate** | | **Standard Error** | | **Z value** | **P value** |
| Intercept | | -4.565 | | 0.403 | | -11.316 | **<0.001** |
| Region (Subtropical) | | 1.536 | | 0.996 | | 1.542 | 0.123 |
| Region (Warm temperate) | | 0.130 | | 0.512 | | 0.254 | 0.799 |
| Shoal Type (Temperate) | | -0.920 | | 0.404 | | -2.277 | **0.023** |
| Species (*M. joyceae*) | | -1.688 | | 0.547 | | -3.086 | **0.002** |
| Type III Wald Chi-Square Test | | **Response Variable:**  **Log Chasing Conspecific Temperate Fish for Temperate Fish Species** | | | | | |
| Variable | **Chi squares** | | **DF** | | **P value** | | |
| Intercept | 128.055 | | 1 | | **<0.001** | | |
| Region | 2.484 | | 2 | | 0.290 | | |
| Shoal Type | 5.184 | | 1 | | **0.023** | | |
| Species | 9.523 | | 1 | | **0.002** | | |

**Table S17:** GLMM, Type III Wald Chi-Square tests and resulting Tukey post hocs of all temperate fish species sheltering behaviour. Final model selected are presented below using log (X+1) transformed sheltering behaviour data. GLMM estimates for fixed, continuous and random effects. Note: **bold** P-value denotes significant effect for predictor variable and corresponding Type III Wald Chi-Squared test. Model AIC = 515.9. Marginal *R_2_* = 0.631 and Conditional *R_2_* = 0.777. DF = Degrees of freedom.

| Final Model: Log sheltering ~ Region + Shoal_Type + Species + (1 \| Region:Location); family: tweedie | | | | |
| --- | --- | --- | --- | --- |
| Variable | **Estimate** | **Standard Error** | **Z value** | **P value** |
| Intercept | -1.880 | 0.932 | -2.016 | **0.044** |
| Region (Subtropical) | -1.330 | 1.123 | -1.185 | 0.236 |
| Region (Warm temperate) | -0.219 | 0.853 | -0.257 | 0.797 |
| Shoal Type (Temperate) | 0.679 | 0.378 | 1.798 | 0.072 |
| Species (*M. strigatus*) | 4.447 | 0.651 | 0.651 | **<0.001** |
| Type III Wald Chi-Square Test | **Response Variable:**  **Log Bite Rate for Temperate Fish Species** | | | |
| Variable | **Chi squares** | **DF** | **P value** |  |
| Intercept | 4.064 | 1 | **0.044** |  |
| Region | 1.519 | 2 | 0.467 |  |
| Shoal Type | 3.234 | 1 | 0.072 |  |
| Species | 46.646 | 1 | **<0.001** |  |

**Table S18:** GLMM, Type III Wald Chi-Square tests and resulting Tukey post hocs of all temperate fish species’ flight initiation distance responses. Final model selected presented below using log (X+1) transformed flight initiation distance data. GLMM estimates for fixed, continuous and random effects. Note: **bold** P-value denotes significant effect for predictor variable and corresponding Type III Wald Chi-Squared test. Model AIC = 224.5. Marginal *R_2_* = 0.249 and Conditional *R_2_* = 0.454. DF = Degrees of freedom.

| Final model: Log FID ~ Region + Species + (1 \| Region:Location) | | | | |
| --- | --- | --- | --- | --- |
| Variable | **Estimate** | **Standard Error** | **Z value** | **P value** |
| Intercept | 2.385 | 0.220 | 10.847 | **< 0.001** |
| Region (Subtropical) | -0.074 | 0.392 | 0.236 | 0.851 |
| Region (Warm temperate) | 0.008 | 0.272 | 0.117 | 0.976 |
| Species (*M. joyceae*) | -0.637 | 0.105 | -0.387 | **<0.001** |
| Type III Wald Chi-Square Test | **Response Variable:**  **Log FID for Temperate Fish Species** | | | |
| Variable | **Chi squares** | **DF** | **P value** |  |
| Intercept | 117.65 | 1 | **< 0.001** |  |
| Region | 0.052 | 2 | 0.974 |  |
| Species | 36.480 | 1 | **<0.001** |  |

**Table S19:** GLMM and Type III Wald Chi-Square tests of the focal temperate fish’s (*Atypichthys strigatus*) relative lateralization (L_R_) responses. Final model selected are presented below using log (X+1) transformed relative lateralization (L_R_) data. GLMM estimates for fixed, continuous and random effects. Note: **bold** P-value denotes significant effect for predictor variable and corresponding Type III Wald Chi-Squared test. Model AIC = 1529.0. Marginal *R_2_* = <0.001 and Conditional *R_2_* = <0.001. DF = Degrees of freedom.

| Final model: Log_L_R_ ~ Region + (1 \| Region:Location); family: gaussian | | | | |
| --- | --- | --- | --- | --- |
| Variable | **Estimate** | **Standard Error** | **Z value** | **P value** |
| Intercept | 6.186 | 4.473 | 1.383 | 0.167 |
| Region (Subtropical) | -2.147 | 16.306 | 0.132 | 0.895 |
| Region (Warm temperate) | 2.356 | 6.619 | 0.356 | 0.722 |
| Type III Wald Chi-Square Test | **Response Variable:**  **Log L_R_ for Temperate Fish Species** | | | |
| Variable | **Chi squares** | **DF** | **P value** |  |
| Intercept | 1.913 | 1 | 0.167 |  |
| Region | 0.131 | 2 | 0.936 |  |

**Table S20:** GLMM, and Type III Wald Chi-Square tests of the focal temperate fish’s (*Atypichthys strigatus*) absolute lateralization (L_A_) responses. Final model selected are presented below using Box-Cox transformed absolute lateralization. GLMM estimates for fixed, continuous and random effects. Note: **bold** P-value denotes significant effect for predictor variable and corresponding Type III Wald Chi-Squared test. Model AIC = 719.1. Marginal *R_2_* = 0.011 and Conditional *R_2_* = 0.012. DF = Degrees of freedom.

| Final model: Box-Cox L_A_ ~ Region + (1 \| Region:Location); family: gaussian | | | | |
| --- | --- | --- | --- | --- |
| Variable | **Estimate** | **Standard Error** | **Z value** | **P value** |
| Intercept | 7.301 | 0.298 | 24.515 | **< 0.001** |
| Region (Subtropical) | 0.223 | 1.094 | 0.204 | 0.838 |
| Region (Warm temperate) | 0.550 | 0.461 | 1.193 | 0.233 |
| Type III Wald Chi-Square Test | **Response Variable:**  **L_A_ for Temperate Fish Species** | | | |
| Variable | **Chi squares** | **DF** | **P value** |  |
| Intercept | 601.004 | 1 | **< 0.001** |  |
| Region | 1.422 | 2 | 0.491 |  |

**Table S21:** GLMM and Type III Wald Chi-Square tests of all temperate fish species’ chasing behaviour towards heterospecific tropical fish responses. Final model selected model presented below uses log (X+1) transformed temperate fish species’ chasing behaviour towards heterospecific tropical fish. GLMM estimates for fixed, continuous and random effects. Model AIC = 3.7. Marginal *R_2_* = 0.982 and Conditional *R_2_* = 0.985. DF = Degrees of freedom.

| Final model: Log_Chasing_Heterospecific_Tropical Fish ~ Region + (1 \| Region:Location); family: tweedie | | | | |
| --- | --- | --- | --- | --- |
| Variable | **Estimate** | **Standard Error** | **Z value** | **P value** |
| Intercept | -34.05 | 59521.63 | -0.001 | 1.000 |
| Region (Subtropical) | 0.001 | 59521.63 | 0.000 | 1.000 |
| Region (Warm temperate) | <0.001 | 59521.63 | 0.000 | 1.000 |
| Type III Wald Chi-Square Test | **Response Variable:**  **Log Chasing Heterospecific Tropical Fish for Temperate Fish Species** | | | |
| Variable | **Chi squares** | **DF** | **P value** |  |
| Intercept | 0.000 | 1 | 1.000 |  |
| Region | 1.742 | 2 | 0.419 |  |

**Table S22:** GLMM, Type III Wald Chi-Square tests and resulting Tukey post hoc tests of all temperate fish species’ chasing behaviour towards heterospecific temperate fish responses. Final model selected model presented below uses log (X+1) transformed temperate fish species’ chasing behaviour towards heterospecific temperate fish data. GLMM estimates for fixed, continuous and random effects. Note: **bold** P-value denotes significant effect for predictor variable and corresponding Type III Wald Chi-Squared test. Model AIC = -39.2. Marginal *R_2_* = 0.750 and Conditional *R_2_* = 0.783. DF = Degrees of freedom.

| Final model: Log Chasing Heterospecific Temperate Fish ~ Region + (1 \| Region:Location); family: tweedie | | | | |
| --- | --- | --- | --- | --- |
| Variable | **Estimate** | **Standard Error** | **Z value** | **P value** |
| Intercept | -6.545 | 1.379 | -4.745 | **<0.001** |
| Region (Subtropical) | -10.607 | 155.730 | -0.068 | 0.946 |
| Region (Warm temperate) | -1.565 | 1.311 | -1.194 | 0.233 |
| Type III Wald Chi-Square Test | **Response Variable:**  **Log Chasing Heterospecific Temperate Fish for Temperate Fish Species** | | | |
| Variable | **Chi squares** | **DF** | **P value** |  |
| Intercept | 22.514 | 1 | **<0.001** |  |
| Region | 1.430 | 2 | 0.489 |  |

**Table S23:** GLMM and Type III Wald Chi-Square tests of all temperate fish species fleeing behaviour from heterospecific temperate fish. Final model selected model presented below uses log (X+1) transformed temperate fish species fleeing behaviour from heterospecific temperate fish data. GLMM estimates for fixed, continuous and random effects. Note: **bold** P-value denotes significant effect for predictor variable and corresponding Type III Wald Chi-Squared test. Model AIC = 8.1. DF = Degrees of freedom. The null model was retained as the best fit model and reported below.

| Final Model: Log_fleeing heterospecific temperate fish~ 1 + (1 \| Region:Location); family: tweedie | | | | |
| --- | --- | --- | --- | --- |
| Variable | **Estimate** | **Standard Error** | **Z value** | **P value** |
| Intercept | NaN | NaN | NaN | NaN |

**Table S24:** GLMM and Type III Wald Chi-Square tests of all temperate fish species fleeing behaviour from conspecific temperate fish. Final model selected model presented below uses log (X+1) transformed temperate fish species fleeing behaviour from conspecific temperate fish data. GLMM estimates for fixed, continuous and random effects. Note: **bold** P-value denotes significant effect for predictor variable and corresponding Type III Wald Chi-Squared test. Model AIC = 24.5. Marginal *R_2_* = 0.158 and Conditional *R_2_* = 0214. DF = Degrees of freedom.

| Final Model: Log_fleeing conspecific temperate fish~ Region + Shoal_Type + Species + (1 \| Region:Location); family: tweedie | | | | |
| --- | --- | --- | --- | --- |
| Variable | **Estimate** | **Standard Error** | **Z value** | **P value** |
| Intercept | -6.716 | 0.626 | -10.738 | **<0.001** |
| Region (Subtropical) | -1.290 | 1.179 | -1.094 | 0.274 |
| Region (Warm temperate) | -1.338 | 0.828 | -1.616 | 0.106 |
| Shoal Type (Temperate) | -0.688 | 0.736 | -0.934 | 0.350 |
| Species | 1.039 | 0.725 | -1.433 | 0.152 |
| Type III Wald Chi-Square Test | **Response Variable:**  **Log fleeing from conspecific temperate for Temperate Fish Species** | | | |
| Variable | **Chi squares** | **DF** | **P value** |  |
| Intercept | 115.298 | 1 | **<0.001** |  |
| Region | 3.142 | 2 | 0.208 |  |
| Shoal Type | 0.873 | 1 | 0.350 |  |
| Species | 2.054 | 1 | 0.152 |  |
